# Supplementary figures and images for: Mapping maternal and infant health in Morocco: A global scoping review of themes, gaps, and the "unseen" in the published health research literature, 2000–2022
Source: PLOS Glob Public Health. 2024 Jul 18;4(7):e0003488. doi: 10.1371/journal.pgph.0003488 (PMC11257357; doi:10.1371/journal.pgph.0003488)

Figure S1. Counts of articles by year and population focus (maternal, infant or both)
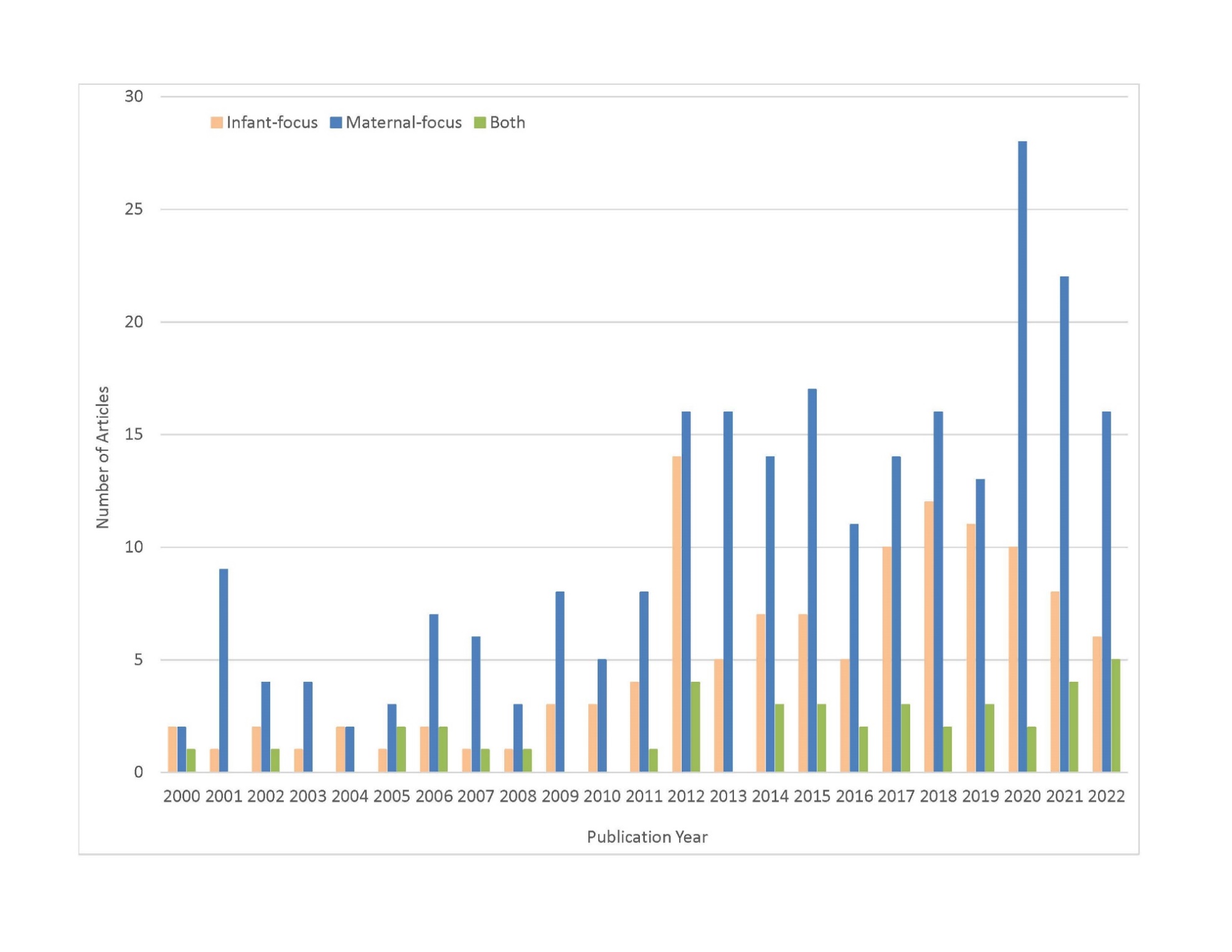

Supplement: S1 Fig — (DOCX) [file pgph.0003488.s001.docx]

Figure S3 Regional distribution of study locations by article count and time period


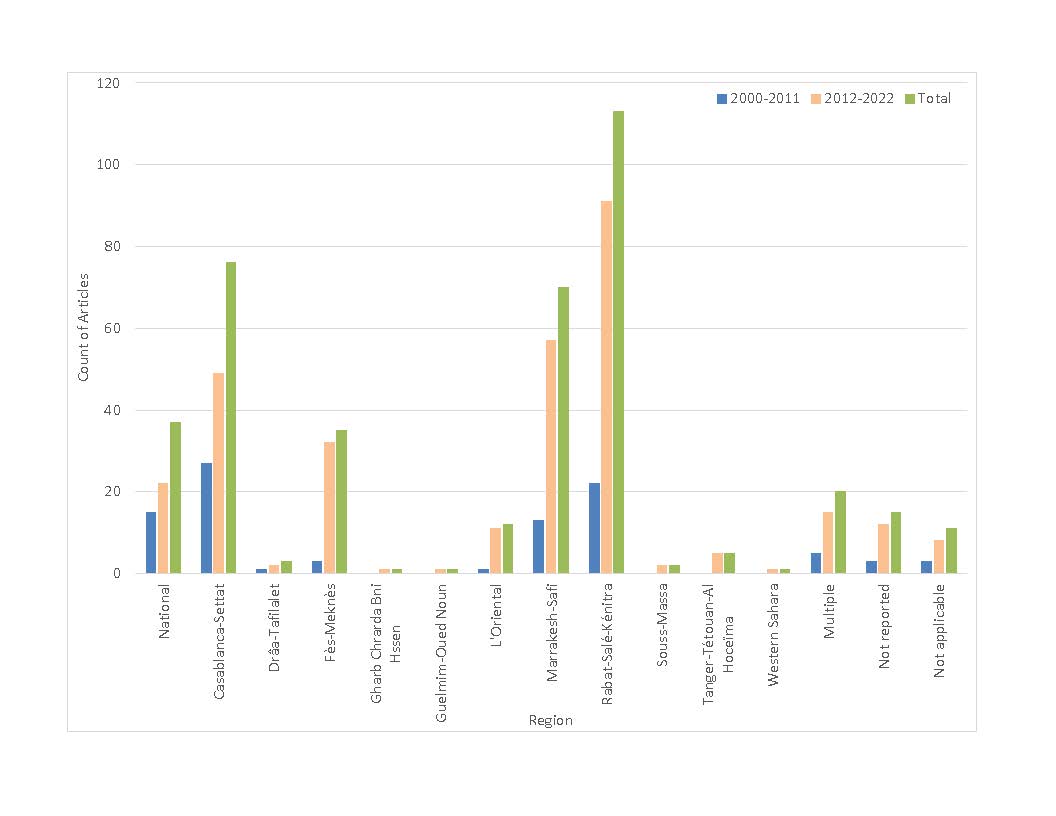

Supplement: S3 Fig — (DOCX) [file pgph.0003488.s003.docx]

Figure S5. Count of articles by theme


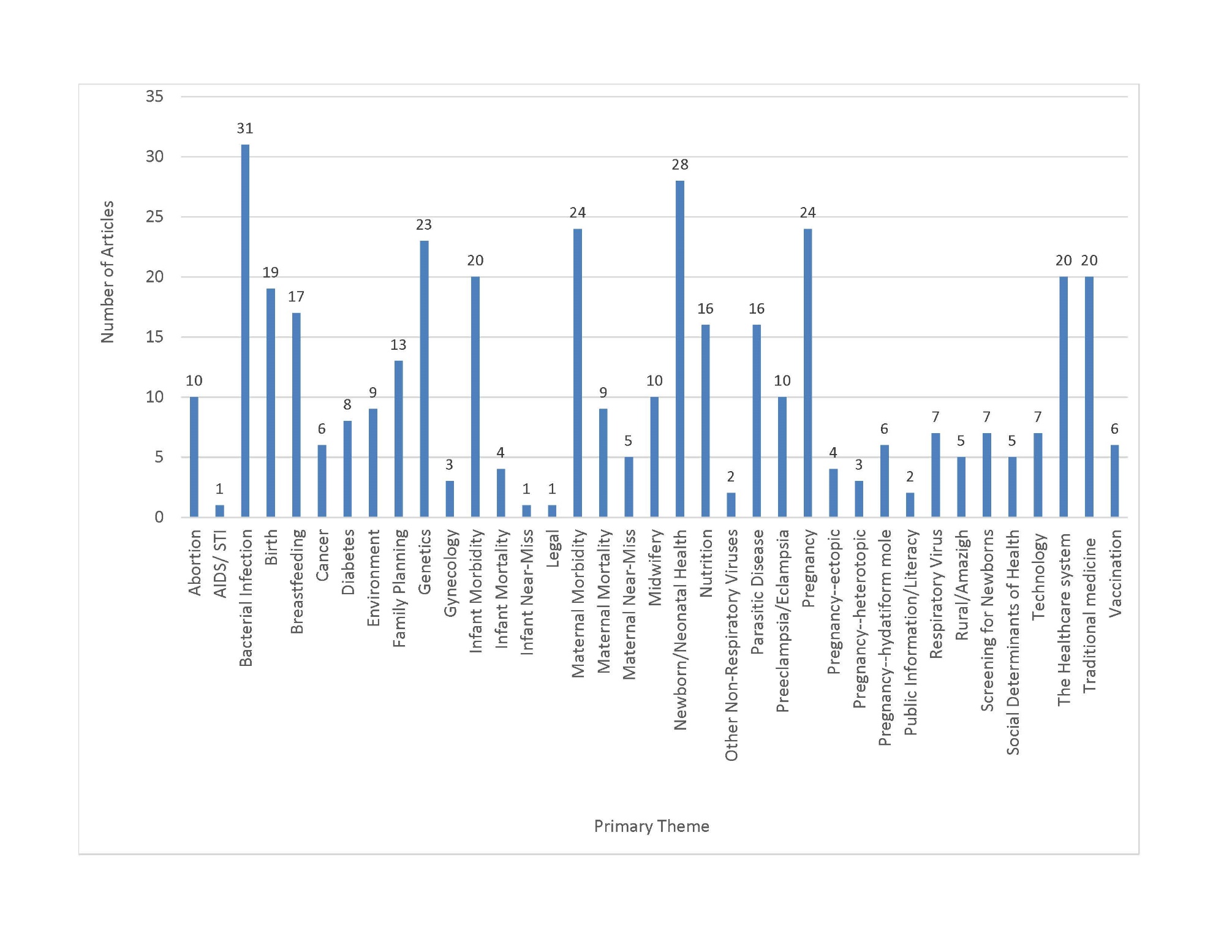

Supplement: S5 Fig — (DOCX) [file pgph.0003488.s005.docx]

Figure S6. Count of articles by population focus (maternal, infant, or both)


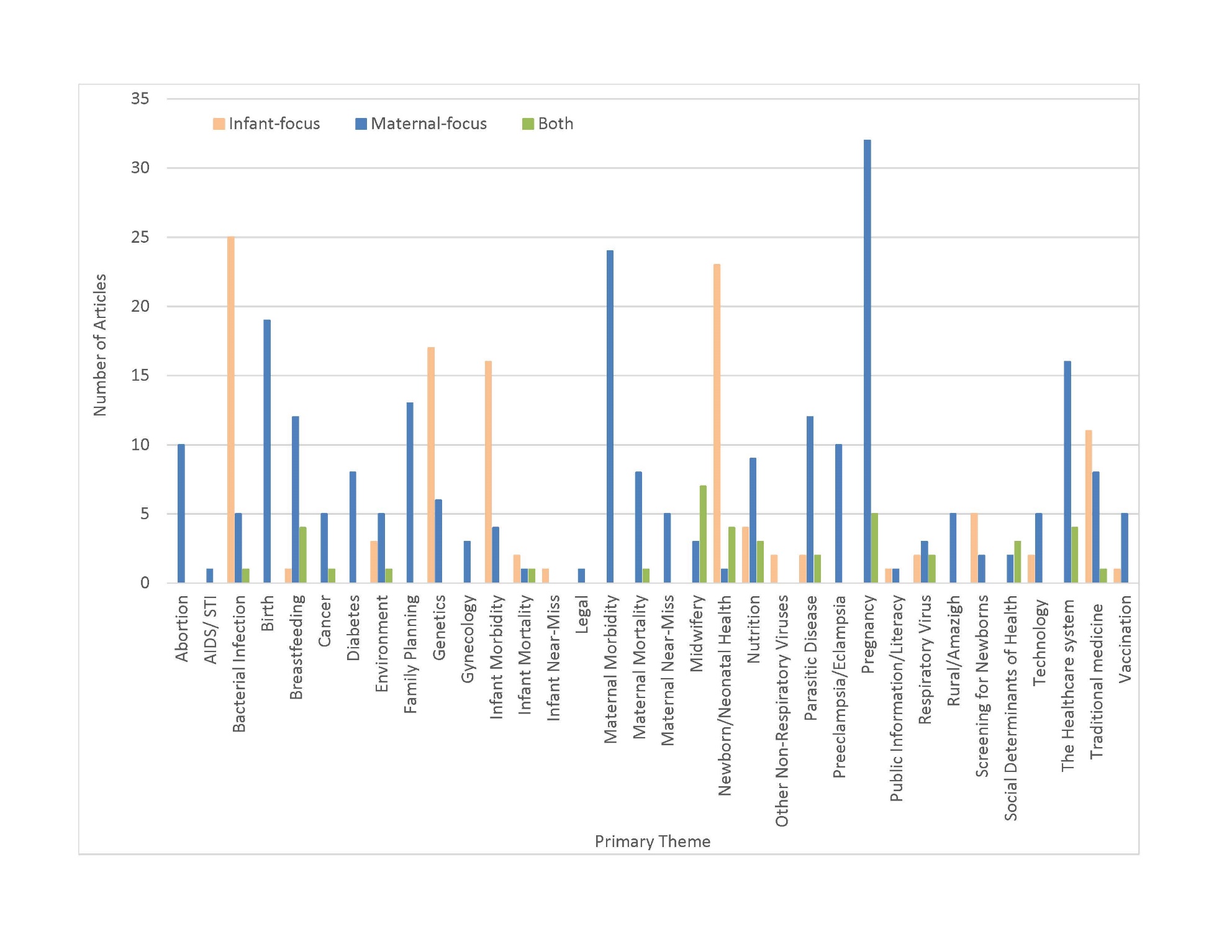

Supplement: S6 Fig — (DOCX) [file pgph.0003488.s006.docx]

Figure S7. Distribution of subgroup MIH articles by primary theme and time period


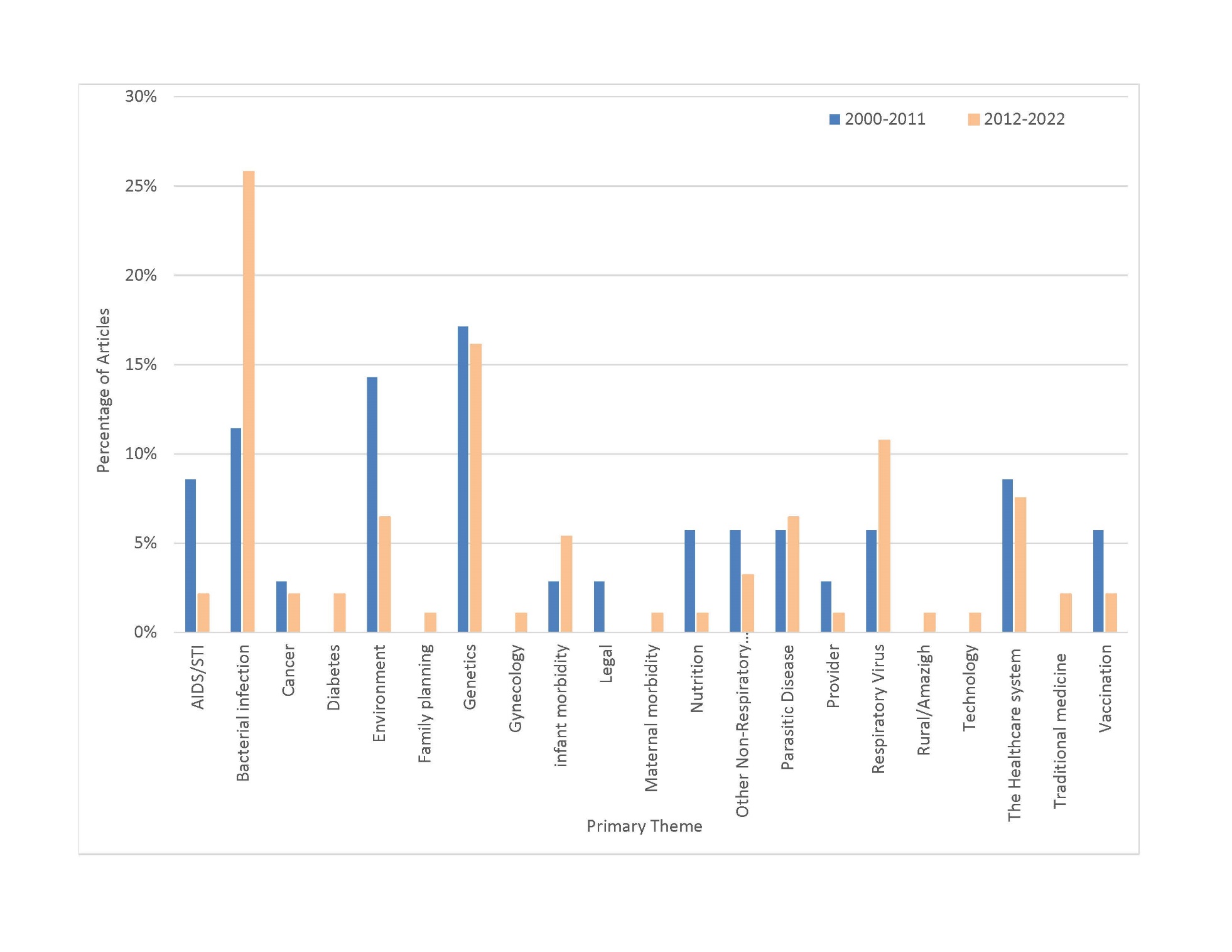

Supplement: S7 Fig — (DOCX) [file pgph.0003488.s007.docx]
