# Supplementary material for: Mapping maternal and infant health in Morocco: A global scoping review of themes, gaps, and the "unseen" in the published health research literature, 2000–2022
Source: PLOS Glob Public Health. 2024 Jul 18;4(7):e0003488. doi: 10.1371/journal.pgph.0003488 (PMC11257357; doi:10.1371/journal.pgph.0003488)
Supplement: S2 Fig — *Method of reporting age varied by study; therefore, age value was approximated using reported means, medians, and mid-points of categorical age ranges. (DOCX) [file pgph.0003488.s002.docx]

Figure S2. Distribution of infant age (days) reported in articles


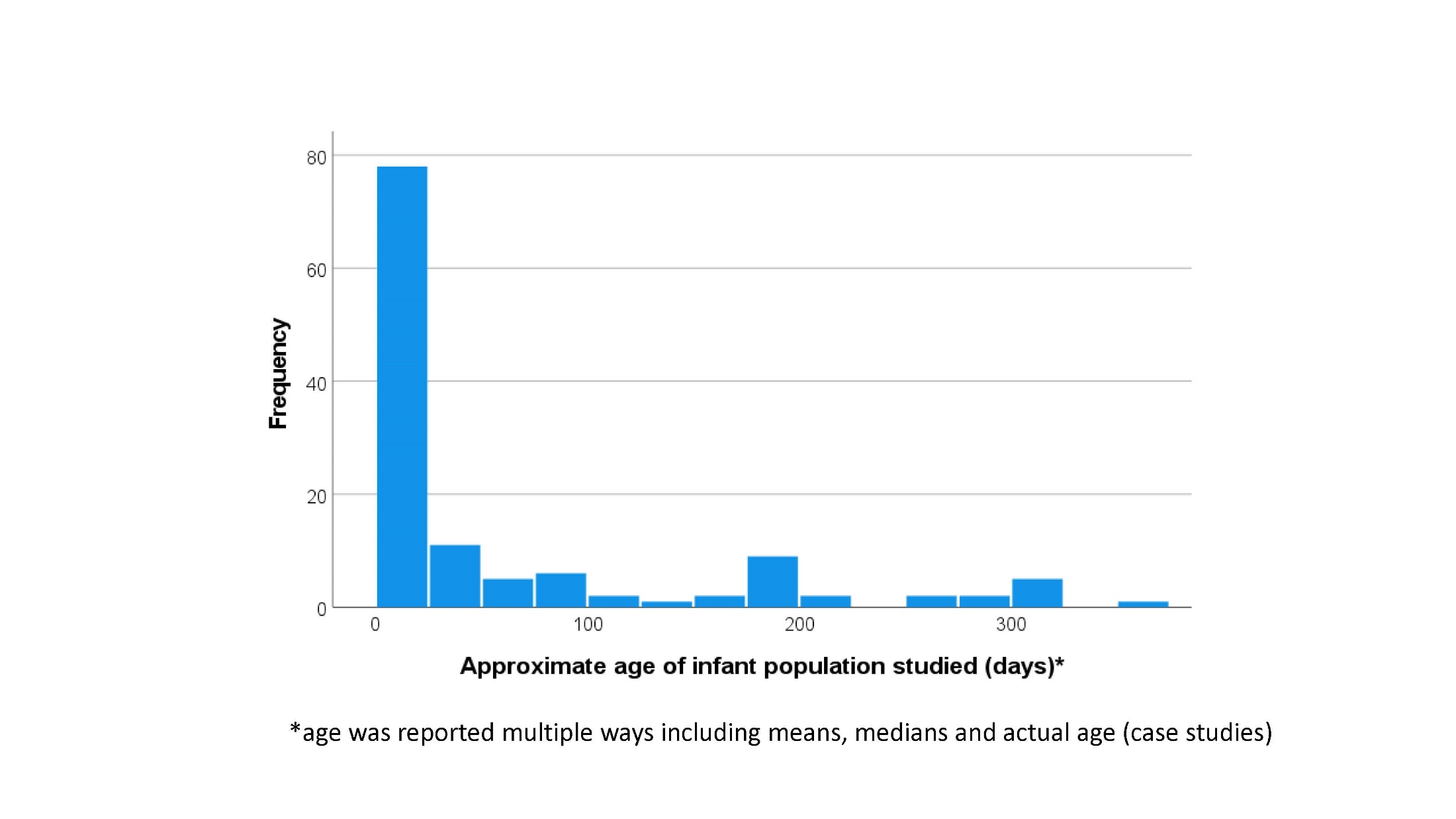


*Method of reporting age varied by study; therefore, age value was approximated using reported means, medians, and mid-points of categorical age ranges
