# Supplementary material for: Mapping maternal and infant health in Morocco: A global scoping review of themes, gaps, and the "unseen" in the published health research literature, 2000–2022
Source: PLOS Glob Public Health. 2024 Jul 18;4(7):e0003488. doi: 10.1371/journal.pgph.0003488 (PMC11257357; doi:10.1371/journal.pgph.0003488)
Supplement: S4 Fig — (DOCX) [file pgph.0003488.s004.docx]

Figure S4. Heat map showing distribution of major health facilities in Morocco by region


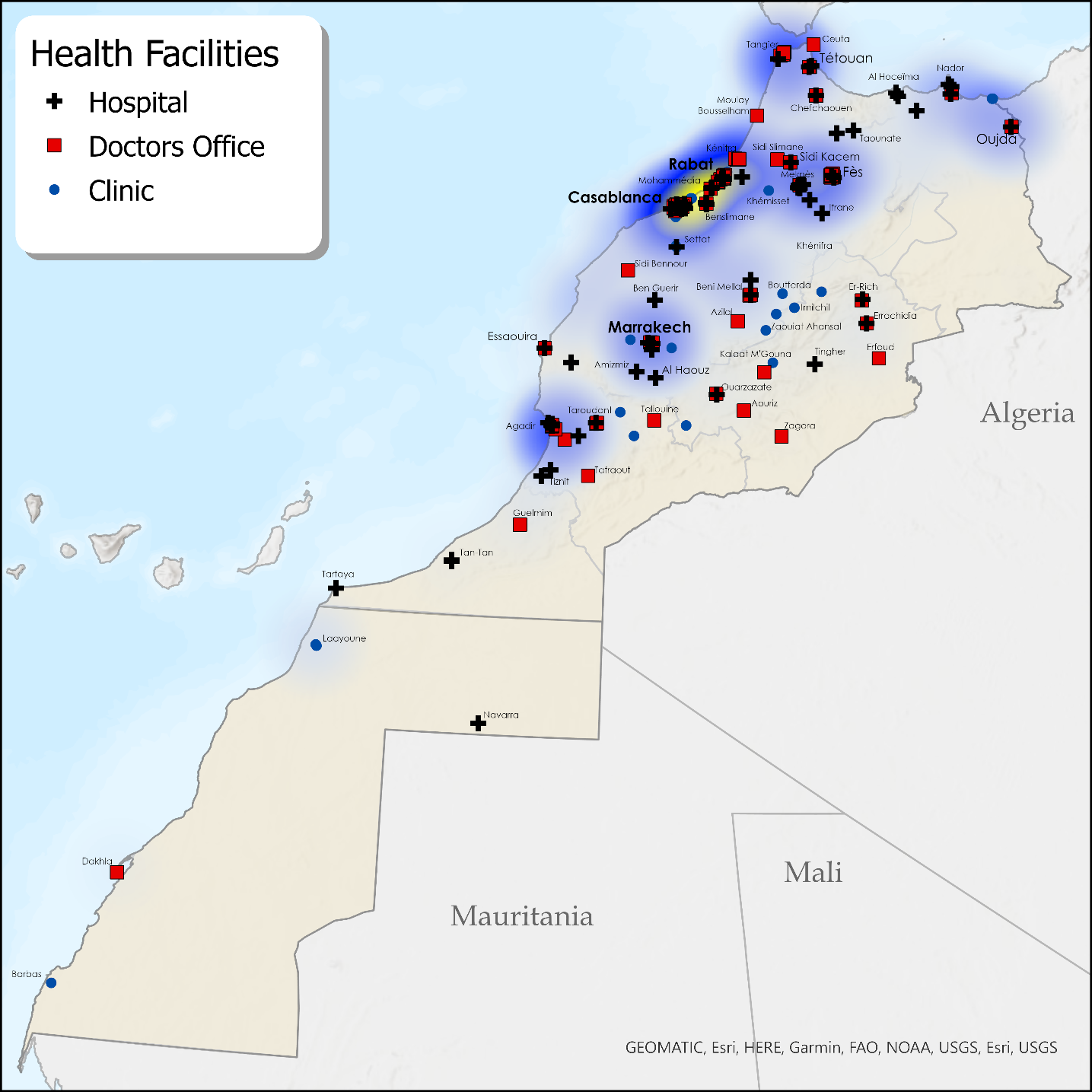


Esri, GEOMATIC, HERE, Garmin, FAO, NOAA, USGS, OpenStreetMap contributors. Maps were created using ArcGIS® software by Esri. ArcGIS® and ArcMap™ are the intellectual property of Esri and are used herein under license. Copyright Esri. All rights reserved. For more information about Esri® software, please visit [www.esri.com](http://www.esri.com/).

United Nations Office for the Coordination of Humanitarian Affairs (OCHA) – Humanitarian Data Exchange, <https://data.humdata.org/dataset/cod-ab-mar>; <https://data.humdata.org/dataset/cod-ab-esh> ; <https://data.humdata.org/dataset/hotosm_mar_populated_places> ; <https://data.humdata.org/dataset/hotosm_mar_health_facilities>, CC BY 4.0
