# Supplementary material for: Mapping maternal and infant health in Morocco: A global scoping review of themes, gaps, and the "unseen" in the published health research literature, 2000–2022
Source: PLOS Glob Public Health. 2024 Jul 18;4(7):e0003488. doi: 10.1371/journal.pgph.0003488 (PMC11257357; doi:10.1371/journal.pgph.0003488)

Figure S8. Approximate percentage of study population composed of mothers or infants in sub-group MIH articles.


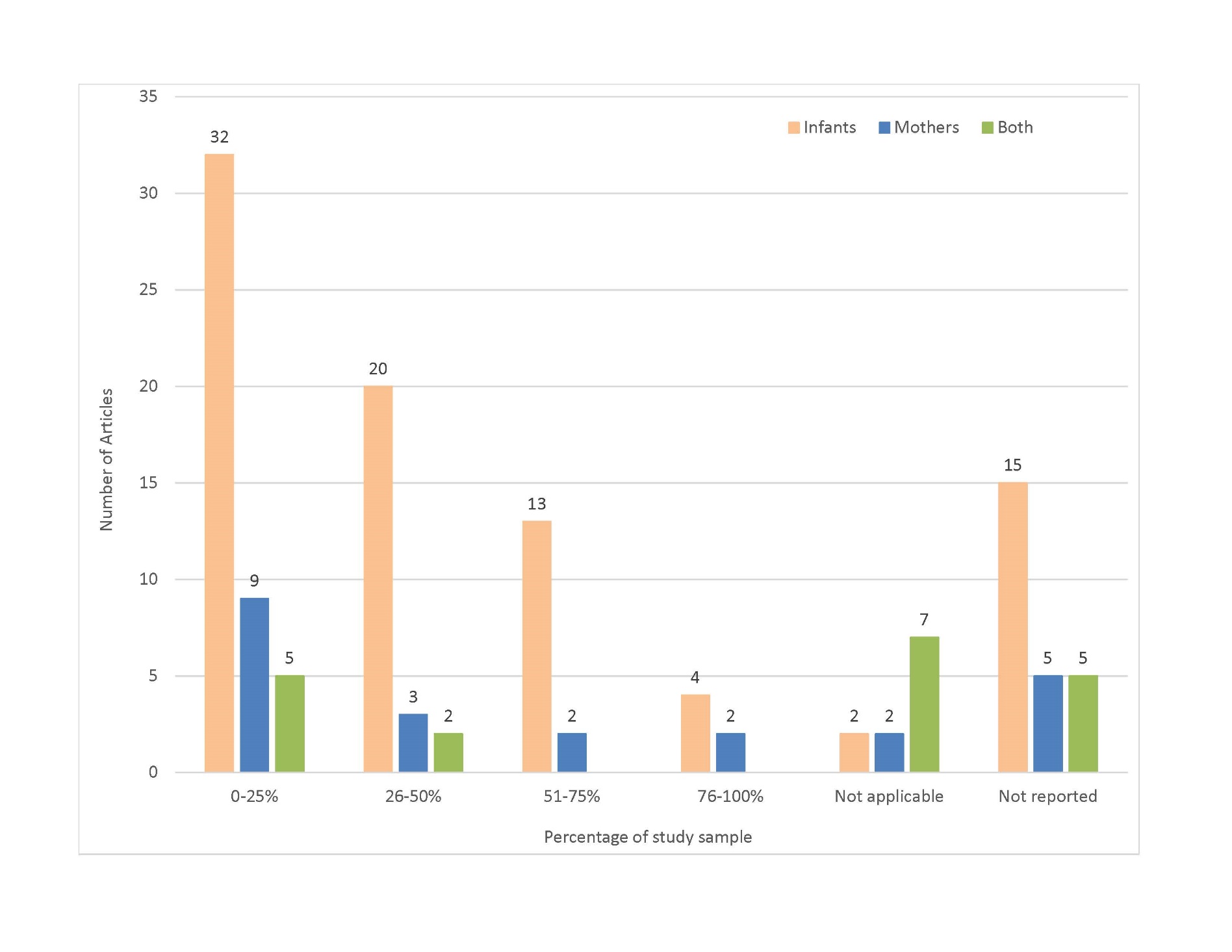

Supplement: S8 Fig — (DOCX) [file pgph.0003488.s008.docx]
