# Supplementary material for: Mapping maternal and infant health in Morocco: A global scoping review of themes, gaps, and the "unseen" in the published health research literature, 2000–2022
Source: PLOS Glob Public Health. 2024 Jul 18;4(7):e0003488. doi: 10.1371/journal.pgph.0003488 (PMC11257357; doi:10.1371/journal.pgph.0003488)
Supplement: S1 Table — (DOCX) [file pgph.0003488.s009.docx]

Table S1: Indicators of Moroccan Maternal and Infant Health across both study time periods (2000-2010 and 2011-2022)

|  | Period 1:  Within 2000-2010 | | Period 2:  Within 2011-2022 | |
| --- | --- | --- | --- | --- |
|  | Year | Value | Year | Value |
| Fertility rate (per woman)^*‖^ | 2000-2004 | 2.8 | 2021 | 2.3 |
| Probability of dying per 1000 live births under 5 years (under-5 mortality rate)^*†^ | 2003 | 39.0 | 2021 | 18.0 |
| Neonatal Mortality Rate (per 1000 live births)^*†^ | 2000 | 21.0 | 2021 | 11.0 |
| Maternal mortality ratio (per 100,000 live births)^*†^ | 2000 | 220.0 | 2020 | 72.0 |
| Low birth weight, prevalence (%)^‡^ | 2000 | 18.3 | 2020 | 14.8 |
| Births attended by skilled health personnel (%)^*†^ | 2003-2004 | 62.6 | 2013-2022 | 87.0 |
| Early initiation of breastfeeding within 1hr of birth (%)^‡^ | 2003 | 48.0 | 2017 | 42.6 |
| Exclusive breastfeeding under 6 months (%)^‡^ | 2003 | 31.0 | 2017 | 35.0 |
| Number of nurses and midwives (per 10,000)^*†^ | 2002 | 9.0 | 2013-2021 | 13.9 |
| *Note:* The data presented in this table are taken from the following sources:    ^*^World Health Organization. World Health Statistics 2005. WHO Press, Geneva Switzerland (available at: <https://iris.who.int/bitstream/handle/10665/43241/9241593261.pdf?sequence=1>). | | | | |
| ^†^World Health Organization. World Health Statistics 2023. WHO Press, Geneva Switzerland (available at: <https://www.who.int/data/gho/publications/world-health-statistics>). | | | | |
| ^‡^World Health Organization. NLiS Data Search: Integrated WHO Nutrition Global Dataset Advanced Search. Copyright 2024 by the WHO (available at:  <https://www.who.int/data/nutrition/nlis/data-search>) | | | | |
| ^‖^The World Bank. Fertility rate, total (births per woman) – Morocco. Copyright 2024 by the World Bank (available at [https://data.worldbank.org/indicator/SP.DYN.TFRT.IN?locations=MA)](https://data.worldbank.org/indicator/SP.DYN.TFRT.IN?locations=MA)%E2%80%AF) | | | | |
