# Supplementary material for: Mapping maternal and infant health in Morocco: A global scoping review of themes, gaps, and the "unseen" in the published health research literature, 2000–2022
Source: PLOS Glob Public Health. 2024 Jul 18;4(7):e0003488. doi: 10.1371/journal.pgph.0003488 (PMC11257357; doi:10.1371/journal.pgph.0003488)
Supplement: S2 Table — (DOCX) [file pgph.0003488.s010.docx]

Table S2. Search strategy

|  | EBSCO: CINAHL |
| --- | --- |
| 1 | TX (“Moroc*” OR “Maroc*”) OR MH “Morocco”+) OR TX (“Western Sahara” OR “High Atlas”) OR TX(“Tanger-Teouan-Al” OR “Tangier” OR “LORiental” OR “Oujda” OR “Fes” OR “Rabat” OR “Beni Mellal*” OR “Casablanca*” OR “Marrakech*” OR “Draa-Tafilalet” OR “Errachidia” OR “Souss-Massa” OR “Agadir” OR “Guelmim*” OR “Laayoune-Sakia El Hamra” OR “El Aaiun” OR “Dakhla*”) OR TX(“berber” or “berbere”) |
| 2 | (MH “PREGNANCY”+) OR (MH pregnant women+ ) OR TX (“matern*” OR “mother” OR “pregnant” OR “pregnancy” OR “pregnancies” OR “gestation”) OR TX (“prepregnancy” OR “pre-pregnancy” OR “pre pregnancy” OR “preconception*” OR “pre conception” OR “pre-conception*” OR “pre conceptionally” OR “periconceptional*”) OR TX (“antenatal*” OR “prenatal*” OR “puerper*” OR “postnatal*” OR “postpartum” OR “peripartum”) |
| 3 | MH “Infant”+ OR TX (“fetal” OR “foetal” OR “fetus” OR “foetus”) OR TX (“baby” OR “babies” OR “neonate” OR “neonatal” OR “preterm” OR “premature”) |
| 4 | (MH “maternal health services”+ OR MH “reproductive health services”+ OR MH “women's health services” OR MH “Maternal-Child Health Centers”) OR ( MH “neonatology” OR MH “perinatology” OR MH “gynecology” OR MH “obstetrics”) OR **(**MH “maternal-child nursing”+ OR MH “midwifery” OR  **MH “**Nurse Midwives” OR MH “obstetric nursing” OR MH “pediatric nursing”+) OR (**MH “**maternal health” OR MH “maternal welfare” OR MH “reproductive health”+ OR MH “sexual health”+ OR MH “reproductive rights”+) OR ( MH “Pregnancy Complications”+ OR MH “Pregnancy Outcome”) OR ( OR MH “infant health”+ OR MH “infant welfare” OR MH “infant mortality”+ OR MH “fetal mortality”) OR ( **MH** “labor, obstetric”+ OR MH “parturition”+ OR MH “contraception”+ OR OR MH “Postnatal Care”+ OR MH “Postpartum Period”+ OR MH “Breast Feeding”+) OR (**MH “**maternal health” OR MH “maternal welfare” OR MH “reproductive health”+ OR MH “sexual health”+ OR MH “reproductive rights”+ OR MH “maternal mortality”+) OR ( MH “Pregnancy Complications”+ OR MH “Pregnancy Outcome” OR **MH** “labor, obstetric”+ OR MH “parturition”+ OR MH “contraception”+ OR MH “Postnatal Care”+ OR MH “Postpartum Period”+ OR MH “Breast Feeding”+) OR ( MH “infant health”+ OR MH “infant welfare” OR MH “infant mortality”+ OR MH “fetal mortality”) |
| 5 | 2 OR 3 OR 4 |
| 6 | 1 AND 5 |

|  | OVID: Embase |
| --- | --- |
| 1 | (Moroc*.mp. or Maroc*.mp. or exp Morocco/) or (Western Sahara or High Atlas).tw. or (Tanger-Teouan-Al or Tangier or LOriental or Oujda or Fes or Rabat or Beni Mellal* or Casablanca* or Marrakech* or Draa-Tafilalet or Errachidia or Souss-Massa or Agadir or Guelmim* or Laayoune-Sakia El Hamra or El Aaiun or Dakhla*).tw. or (berber or berbere).mp. |
| 2 | (exp PREGNANCY/) OR (exp pregnant women/) OR (matern* OR mother OR pregnant OR pregnancy OR pregnancies OR gestation).mp. OR (prepregnancy OR pre-pregnancy OR pre pregnancy OR preconception* OR pre conception OR pre-conception* OR pre conceptionally OR periconceptional*).mp. OR (antenatal* OR prenatal* OR puerper* OR postnatal* OR postpartum OR peripartum).mp. |
| 3 | Exp Infant/ OR (fetal OR foetal OR fetus OR foetus).mp. OR (baby OR babies OR neonate OR neonatal OR preterm OR premature).mp. |
| 4 | (exp maternal health services/ OR reproductive health services.mp. OR women's health services.mp. OR exp Maternal Child Health Care/) OR (exp neonatology/ OR exp perinatology/ OR exp gynecology/ OR exp obstetrics/) OR **(**maternal-child nursing.mp. OR exp midwife/ OR exp Nurse Midwife/ OR exp obstetrical nursing/ OR exp pediatric nursing/ ) OR maternal health/ OR exp maternal welfare/ OR exp reproductive health/ OR exp sexual health/ OR exp reproductive rights/ OR exp maternal mortality/ OR exp maternal morbidity/ or exp perinatal morbidity/ OR ( exp child health/ OR infant welfare/ OR exp infant mortality/ OR exp fetus mortality/ OR exp newborn morbidity/) OR ( exp labor/ OR exp birth/ OR exp contraception/ OR exp Pregnancy Complications/ OR exp Pregnancy Outcome/ OR exp Pregnancy Complications/ OR exp Pregnancy Outcome/) |
| 5 | 2 OR 3 OR 4 |
| 6 | 1 and 5 |
| **7** | Women's Health/ OR maternal health/ OR exp maternal welfare/ OR exp reproductive health/ OR exp sexual health/ OR exp reproductive rights/ OR women's rights/ OR exp maternal mortality/ OR exp maternal morbidity/ or exp perinatal morbidity/ or exp newborn morbidity/ OR exp Pregnancy Complications/ OR exp Pregnancy Outcome/ OR  **exp** Labor Pain/ OR exp child health/ OR infant welfare/ OR exp infant mortality/ OR exp fetus mortality/ OR exp Newborn Diseases/ OR exp birth weight/ OR fetus weight/ OR Apgar Score/ OR exp sex counseling/ OR exp sex education/ OR exp nursing education/ OR exp Obstetric operation/ OR exp labor/ OR exp birth/ OR exp contraception/ OR exp fetal therapy/ OR exp pregnancy diabetes mellitus/ OR exp Postnatal Care/ OR exp puerperium/ OR exp Breast Feeding/ OR exp Human immunodeficiency virus/ OR exp breast tumor/ OR exp uterus cancer/ OR exp Alphapapillomavirus/ OR Acquired Immunodeficiency Syndrome/ OR exp Attitude to Health/ OR exp Protozoal Infection/ OR exp communicable diseases/ OR water borne diseases/ OR exp parasitosis/ OR "Social Determinants of Health"/ OR exp health care quality/ OR Clinical Competence/ OR transcultural care/ OR Healthcare Disparity/ OR Awareness/ OR Self Care/ OR doctor patient relationship/ OR rural health nursing/ OR exp community health nursing/ OR family nursing/ OR cultural nursing/ OR exp non profit organization/ OR exp telemedicine/ OR (paramedicine OR community networks OR volunteers OR foundation).mp. OR global health/ OR exp rural health care/ |
| **8** | **6 and 7** |

|  | OVID:Psychinfo & MEDLINE |
| --- | --- |
| 1 | (Moroc*.mp. or Maroc*.mp.) or (Western Sahara or High Atlas).tw. or (Tanger-Teouan-Al or Tangier or LOriental or Oujda or Fes or Rabat or Beni Mellal* or Casablanca* or Marrakech* or Draa-Tafilalet or Errachidia or Souss-Massa or Agadir or Guelmim* or Laayoune-Sakia El Hamra or El Aaiun or Dakhla*).tw. or (berber or berbere).mp. |
| 2 | (exp PREGNANCY/ OR exp Mothers/) OR (matern* OR mother OR pregnant OR pregnancy OR pregnancies OR gestation).mp. OR (prepregnancy OR pre-pregnancy OR pre pregnancy OR preconception* OR pre conception OR pre-conception* OR pre conceptionally OR periconceptional*).mp. OR (antenatal* OR prenatal* OR puerper* OR postnatal* OR postpartum OR peripartum).mp. |
| 3 | Infant.mp. OR (exp Fetus/) or (fetal OR foetal OR fetus OR foetus).mp. OR (baby OR babies OR neonate OR neonatal OR preterm OR premature).mp. OR exp Premature Birth/ OR exp Neonatal Period/ |
| 4 | (maternal health services.mp. OR reproductive health services.mp. OR women's health services.mp. OR Maternal-Child Health Centers.mp.) OR (exp Neonatal Intensive Care/ ) OR (neonatology.mp. OR perinatology.mp. OR exp Gynecology/ OR exp Obstetrics/) OR **(**maternal-child nursing.mp. OR exp Midwifery/ OR Nurse Midwives.mp. OR obstetric nursing.mp. OR pediatric nursing.mp. ) OR ( maternal health.mp. OR maternal welfare.mp. OR exp reproductive health/ OR exp sexual health/ OR reproductive rights.mp.) OR ( maternal mortality.mp. OR exp Obstetrical Complications/ or exp Pregnancy Outcomes/ OR exp "Labor (Childbirth)"/ or exp Birth/ OR parturition.mp. OR exp Birth Control/ OR exp Prenatal Exposure/ OR exp Prenatal Care/ OR Postnatal Care/ OR exp Postnatal Period/ or exp Perinatal Period/ or Postpartum.mp. OR exp Postpartum Depression/ OR exp Breast Feeding/ ) OR (infant health.mp. OR infant welfare.mp. OR infant mortality.mp. OR fetal mortality.mp.) |
| 5 | 2 OR 3 OR 4 |
| 6 | 1 and 5 |
