# Supplementary material for: Mapping maternal and infant health in Morocco: A global scoping review of themes, gaps, and the "unseen" in the published health research literature, 2000–2022
Source: PLOS Glob Public Health. 2024 Jul 18;4(7):e0003488. doi: 10.1371/journal.pgph.0003488 (PMC11257357; doi:10.1371/journal.pgph.0003488)
Supplement: S3 Table — (DOCX) [file pgph.0003488.s011.docx]

Table S3 Extraction variables

| CATEGORY | VARIABLE | VARIABLE DESCRIPTION | SUBGROUP EXTRACTION |
| --- | --- | --- | --- |
| Article | ID | Unique ID from Covidence | X |
|  | Title | Title of article | X |
|  | Year | Year of publication | X |
|  | Journal | Journal title | X |
|  | Language | Language of publication | X |
|  | Type | Type of article |  |
| General study Info | Open Access | Article published open access |  |
|  | First author |  |  |
|  | Author country | Country where primary author located |  |
|  | Study Type | Study design as described by author |  |
|  | Longitudinal | Was the data collected longitudinal? |  |
|  | Sample | Data collected from hospital/clinic or community | X |
|  | Objective |  |  |
|  | MIH primary topic | Was the topic related primarily to MIH? | X |
| Moroccan Study Location | % Moroccan population | Percentage of study sample from Morocco |  |
|  | Rural/Urban | Was the study conducted in an urban or rural area? | X |
|  | Region | List region(s) where study conducted | X |
|  | City | List cities where study conducted |  |
|  | Multi-site Study | Did the study occur at more than one hospital/site? |  |
|  | Hospital or Institute Name | List hospital(s) or institution(s) where the study was conducted | X |
|  | Type of hospital | Type of hospital | X |
|  | % Sample from hospital | Percentage of the sample was recruited from a hospital/clinic |  |
| Study population (Morocco) | Mother/infant pair | Did the study examine mother-infant pairs? | X |
|  | N | Total sample size |  |
|  | Subgroup N | Sample size for mother or infant sub-group | X |
|  | Condition | List condition/disease described in the article | X |
|  | Procedure | List medical procedures described in the article |  |
|  | Age (median) | Median age of MIH group |  |
|  | Age (mean) | Mean age of MIH group |  |
|  | Age(SD) | Standard deviation of age for MIH group |  |
|  | Age (min) | Minimum age of MIH group |  |
|  | Age (max) | Maximum age of MIH group |  |
|  | Gender | Percentage of females included in the study |  |
|  | Rural/Urban | Where does study population reside? |  |
|  | % Urban | Percentage of the study sample that is Urban |  |
|  | % Amazigh | Percentage of the study sample reported as Amazigh |  |
| Outcome | Death | Was the outcome of the case study death? |  |
| THEMES | Level | Geographic level of the study | X |
|  | stage | Stage of MIH researched (e.g. preterm, prenatal, pregnancy)? | X |
|  | Focus | What group was the focus of the study (i.e. Patient, provider) |  |
|  | Policy | Did this study examine or policy or make recommendation(s)? |  |
|  | Perspective | Data perspective (e.g. patient, provider) |  |
|  | Primary data source | Primary source of data (eg, patient questionnaire) |  |
|  | Secondary data source | Secondary source of data (if any) |  |
|  | Primary theme | Primary theme addressed by the article | X |
|  | Secondary theme | Secondary theme addressed by the article | X |
|  | Other theme(s) | Additional theme(s) addressed | X |
|  | Notes | Additional notes or comments | X |
