# Supplementary material for: Mapping maternal and infant health in Morocco: A global scoping review of themes, gaps, and the "unseen" in the published health research literature, 2000–2022
Source: PLOS Glob Public Health. 2024 Jul 18;4(7):e0003488. doi: 10.1371/journal.pgph.0003488 (PMC11257357; doi:10.1371/journal.pgph.0003488)
Supplement: S4 Table — (DOCX) [file pgph.0003488.s012.docx]

Table S4 Relevant data for full-text articles

| **Author** | **Year** | **Language** | **Type** | **Focus** | **Primary theme** | **Reference Supplement S1** |
| --- | --- | --- | --- | --- | --- | --- |
| Hessini et al | 2007 | English | Perspective | maternal | Abortion | 1 |
| Abidi et al | 2008 | English | Case study | maternal | Abortion | 2 |
| Belhouss et al | 2011 | French | Letter | maternal | Abortion | 3 |
| Berdai et al | 2014 | English | Case study | maternal | Abortion | 4 |
| Chraibi et al | 2014 | French | Letter | maternal | Abortion | 5 |
| Foster et al | 2014 | English | Research | maternal | Abortion | 6 |
| Laghzaoui et al | 2016 | French | Research | maternal | Abortion | 7 |
| Capeeli et al | 2019 | English | Research | maternal | Abortion | 8 |
| Ghizlane et al | 2021 | English | Case study | maternal | Abortion | 9 |
| Mourchid et al | 2022 | French | Research | maternal | Abortion | 10 |
| Chakib et al | 2001 | French | Research | maternal | AIDS/STI | 11 |
| Aitmhand et al | 2000 | English | Brief/ short communication/ report | infants | Bacterial Infection | 12 |
| Benomar et al | 2000 | French | Letter | infants | Bacterial Infection | 13 |
| Nejjari et al | 2002 | English | Research | infants | Bacterial Infection | 14 |
| World health organization et al | 2002 | English | Brief/ short communication/ report | infants | Bacterial Infection | 15 |
| Nejjari et al | 2003 | French | Case study | infants | Bacterial Infection | 16 |
| Ailal et al | 2004 | French | Research | infants | Bacterial Infection | 17 |
| Balaka et al | 2004 | French | Research | infants | Bacterial Infection | 18 |
| Oulahiane et al | 2005 | French | Research | infants | Bacterial Infection | 19 |
| Benchellal et al | 2009 | French | Research | maternal | Bacterial Infection | 20 |
| Rosenthal et al | 2011 | English | Research | infants | Bacterial Infection | 21 |
| Chemsi et al | 2012 | French | Research | infants | Bacterial Infection | 22 |
| Rosenthal et al | 2012 | English | Research | infants | Bacterial Infection | 23 |
| Aseri et al | 2014 | French | Research | infants | Bacterial Infection | 24 |
| Maoulainine et al | 2014 | French | Research | infants | Bacterial Infection | 25 |
| Ballen et al | 2015 | English | Letter | maternal | Bacterial Infection | 26 |
| Chemsi et al | 2015 | French | Research | infants | Bacterial Infection | 27 |
| Elatiqi et al | 2015 | French | Case study | infants | Bacterial Infection | 28 |
| Bassir et al | 2016 | French | Research | maternal | Bacterial Infection | 29 |
| Chabah et al | 2016 | English | Case study | infants | Bacterial Infection | 30 |
| Saez-Lopez et al | 2016 | English | Research | maternal | Bacterial Infection | 31 |
| Arhoune et al | 2017 | English | Research | infants | Bacterial Infection | 32 |
| Chemsi et al | 2018 | French | Research | infants | Bacterial Infection | 33 |
| Daoudi et al | 2018 | English | Case study | infants | Bacterial Infection | 34 |
| Moraleda et al | 2018 | English | Research | Both | Bacterial Infection | 35 |
| Arhoune et al | 2019 | English | Research | infants | Bacterial Infection | 36 |
| Taoufik et al | 2019 | English | Research | infants | Bacterial Infection | 37 |
| Touhami et al | 2019 | French | Case study | infants | Bacterial Infection | 38 |
| Elgarini et al | 2020 | French | Research | maternal | Bacterial Infection | 39 |
| Arhoune et al | 2021 | English | Research | infants | Bacterial Infection | 40 |
| Jbari et al | 2022 | English | Research | infants | Bacterial Infection | 41 |
| Nachate et al | 2022 | English | Research | infants | Bacterial Infection | 42 |
| Obermeyer et al | 2000 | English | Research | maternal | Birth | 43 |
| Obermeyer et al | 2000 | English | Research | maternal | Birth | 44 |
| Sefrioui et al | 2001 | French | Research | maternal | Birth | 45 |
| Sefrioui et al | 2002 | French | Case study | maternal | Birth | 46 |
| Hamada et al | 2004 | French | Research | maternal | Birth | 47 |
| Paxton et al | 2006 | English | Research | maternal | Birth | 48 |
| Leone et al | 2008 | English | Research | maternal | Birth | 49 |
| Khawaja et al | 2009 | English | Research | maternal | Birth | 50 |
| Capelli et al | 2011 | English | Research | maternal | Birth | 51 |
| Kyu et al | 2013 | English | Research | maternal | Birth | 52 |
| Boutayeb et al | 2015 | English | Letter | maternal | Birth | 53 |
| Cresswell et al | 2015 | English | Research | maternal | Birth | 54 |
| Elghanmi et al | 2015 | English | Case study | maternal | Birth | 55 |
| Sabiri et al | 2015 | English | Research | maternal | Birth | 56 |
| Benzouina et al | 2016 | English | Research | maternal | Birth | 57 |
| Benkirane et al | 2017 | French | Research | maternal | Birth | 58 |
| Xu et al | 2017 | English | Research | maternal | Birth | 59 |
| Hua et al | 2019 | English | Research | maternal | Birth | 60 |
| Zaidi et al | 2020 | English | Research | maternal | Birth | 61 |
| Hassani et al | 2005 | French | Research | Both | Breastfeeding | 62 |
| Roida et al | 2010 | French | Research | maternal | Breastfeeding | 63 |
| Thimou Izgua et al | 2012 | French | Research | Both | Breastfeeding | 64 |
| Adarmouch et al | 2013 | French | Research | maternal | Breastfeeding | 65 |
| Bouhouch et al | 2014 | English | Research | maternal | Breastfeeding | 66 |
| Berrani et al | 2015 | French | Research | Both | Breastfeeding | 67 |
| Dold et al | 2017 | English | Research | maternal | Breastfeeding | 68 |
| Hamada et al | 2017 | French | Research | Both | Breastfeeding | 69 |
| Habibi et al | 2018 | English | Research | maternal | Breastfeeding | 70 |
| Jasny et al | 2019 | English | Research | maternal | Breastfeeding | 71 |
| Laamiri et al | 2019 | French | Research | maternal | Breastfeeding | 72 |
| Ennasser et al | 2020 | French | Case study | maternal | Breastfeeding | 73 |
| Hamada et al | 2020 | English | Research | maternal | Breastfeeding | 74 |
| Mekaoui et al | 2020 | English | Letter | maternal | Breastfeeding | 75 |
| Mulol et al | 2020 | English | Research | infants | Breastfeeding | 76 |
| El Moussaoui et al | 2021 | English | Research | maternal | Breastfeeding | 77 |
| Rabi et al | 2021 | English | Research | maternal | Breastfeeding | 78 |
| Soliman et al | 2009 | English | Research | maternal | Cancer | 79 |
| Tissir et al | 2012 | French | Research | Both | Cancer | 80 |
| Boufettal et al | 2014 | French | Research | maternal | Cancer | 81 |
| Affdal et al | 2020 | English | Research | maternal | Cancer | 82 |
| Driouich et al | 2021 | English | Research | maternal | Cancer | 83 |
| Reynaud et al | 2021 | English | Research | maternal | Cancer | 84 |
| Bouhsain et al | 2009 | French | Research | maternal | Diabetes | 85 |
| Bouhsain et al | 2014 | French | Research | maternal | Diabetes | 86 |
| Utz et al | 2016 | English | Research | maternal | Diabetes | 87 |
| Utz et al | 2017 | English | Protocol paper | maternal | Diabetes | 88 |
| Utz et al | 2017 | English | Research | maternal | Diabetes | 89 |
| Utz et al | 2018 | English | Research | maternal | Diabetes | 90 |
| Chamlal et al | 2020 | English | Research | maternal | Diabetes | 91 |
| Utz et al | 2020 | English | Research | maternal | Diabetes | 92 |
| Souad et al | 2006 | English | Research | infants | Environment | 93 |
| Zaida et al | 2007 | English | Research | infants | Environment | 94 |
| El Koraichi et al | 2011 | French | Letter | infants | Environment | 95 |
| Cherkani-Hassani et al | 2020 | English | Research | maternal | Environment | 96 |
| Cherkani-Hassani et al | 2020 | English | Research | maternal | Environment | 97 |
| Cherkani-Hassani et al | 2021 | English | Research | maternal | Environment | 98 |
| Cherkani-Hassani et al | 2021 | English | Research | maternal | Environment | 99 |
| Jeddi et al | 2021 | English | Research | Both | Environment | 100 |
| Cherkani-Hassani et al | 2022 | English | Research | maternal | Environment | 101 |
| Crognier et al | 2001 | English | Research | maternal | Family Planning | 102 |
| Eltigani et al | 2001 | English | Research | maternal | Family Planning | 103 |
| Crognier et al | 2003 | English | Research | maternal | Family Planning | 104 |
| Agha et al | 2006 | English | Research | maternal | Family Planning | 105 |
| Crognier et al | 2006 | English | Research | maternal | Family Planning | 106 |
| Sullivan et al | 2006 | English | Research | maternal | Family Planning | 107 |
| Agha et al | 2008 | English | Research | maternal | Family Planning | 108 |
| Ali et al | 2010 | English | Research | maternal | Family Planning | 109 |
| Hughes et al | 2011 | English | Research | maternal | Family Planning | 110 |
| Ali et al | 2014 | English | Research | maternal | Family Planning | 111 |
| Rinker et al | 2015 | English | Perspective | maternal | Family Planning | 112 |
| Benbella et al | 2018 | English | Research | maternal | Family Planning | 113 |
| Benbella et al | 2018 | English | Research | maternal | Family Planning | 114 |
| Jaouad et al | 2008 | English | Case study | infants | Genetics | 115 |
| Zakaria et al | 2009 | French | Case study | infants | Genetics | 116 |
| Ratbi et al | 2010 | English | Case study | infants | Genetics | 117 |
| Achargui et al | 2011 | French | Research | maternal | Genetics | 118 |
| Sbiti et al | 2011 | French | Research | infants | Genetics | 119 |
| Doubaj et al | 2012 | English | Research | infants | Genetics | 120 |
| Lamzouri et al | 2012 | English | Research | infants | Genetics | 121 |
| Lamzouri et al | 2012 | French | Research | maternal | Genetics | 122 |
| Lyahyai et al | 2012 | English | Research | infants | Genetics | 123 |
| Tajir et al | 2012 | English | Research | infants | Genetics | 124 |
| Doubaj et al | 2015 | English | Case study | infants | Genetics | 125 |
| Es-Seddiki et al | 2015 | French | Case study | infants | Genetics | 126 |
| Zerkaoui et al | 2015 | English | Case study | infants | Genetics | 127 |
| Jouali et al | 2018 | English | Case study | infants | Genetics | 128 |
| Elkarhat et al | 2019 | English | Research | maternal | Genetics | 129 |
| Laghmich et al | 2019 | English | Research | infants | Genetics | 130 |
| Zahir et al | 2019 | French | Research | infants | Genetics | 131 |
| El Moussaoui et al | 2020 | English | Case study | infants | Genetics | 132 |
| Lehlimi et al | 2020 | French | Research | maternal | Genetics | 133 |
| Ouattaleb et al | 2020 | French | Research | maternal | Genetics | 134 |
| Benhsaien et al | 2021 | English | Case study | infants | Genetics | 135 |
| Meiouet et al | 2021 | French | Research | maternal | Genetics | 136 |
| van Wegberg et al | 2021 | English | Research | infants | Genetics | 137 |
| Zouhair et al | 2002 | French | Research | maternal | Gynecology | 138 |
| Kadi et al | 2012 | English | Case study | maternal | Gynecology | 139 |
| Lamrissi et al | 2022 | English | Case study | maternal | Gynecology | 140 |
| Bourrous et al | 2010 | French | Research | infants | Infant Morbidity | 141 |
| Arsalane et al | 2012 | French | Letter | maternal | Infant Morbidity | 142 |
| Dibi et al | 2012 | French | Case study | infants | Infant Morbidity | 143 |
| Rami et al | 2012 | French | Case study | infants | Infant Morbidity | 144 |
| Elghanni et al | 2013 | English | Research | infants | Infant Morbidity | 145 |
| Elmachtani Idrissi et al | 2013 | French | Case study | infants | Infant Morbidity | 146 |
| Baba et al | 2014 | English | Case study | infants | Infant Morbidity | 147 |
| Maleb et al | 2014 | English | Case study | infants | Infant Morbidity | 148 |
| Radouani et al | 2015 | French | Research | maternal | Infant Morbidity | 149 |
| El Fakiri et al | 2016 | French | Research | infants | Infant Morbidity | 150 |
| El Amraoui et al | 2017 | English | Case study | infants | Infant Morbidity | 151 |
| Es-Seddiki et al | 2017 | French | Research | infants | Infant Morbidity | 152 |
| Indrio et al | 2017 | English | Research | maternal | Infant Morbidity | 153 |
| Ouladsaiad et al | 2017 | English | Case study | infants | Infant Morbidity | 154 |
| Vandenplas et al | 2017 | English | Research | maternal | Infant Morbidity | 155 |
| Lahrichi et al | 2018 | English | Research | infants | Infant Morbidity | 156 |
| Elghanmi et al | 2020 | English | Research | infants | Infant Morbidity | 157 |
| Forci et al | 2020 | English | Research | infants | Infant Morbidity | 158 |
| Forci et al | 2021 | English | Research | infants | Infant Morbidity | 159 |
| Saiad et al | 2021 | English | Research | infants | Infant Morbidity | 160 |
| El Masnaoui et al | 2009 | French | Research | maternal | Infant Mortality | 161 |
| Sabiri et al | 2012 | English | Brief/ short communication/ report | infants | Infant Mortality | 162 |
| Ouahid et al | 2019 | French | Research | Both | Infant Mortality | 163 |
| Nya et al | 2021 | English | Case study | infants | Infant Mortality | 164 |
| Ronsmans et al | 2016 | English | Research | infants | Infant Near-Miss | 165 |
| Newman et al | 2019 | English | Perspective | maternal | Legal | 166 |
| Hachim et al | 2001 | French | Research | maternal | Maternal Morbidity | 167 |
| Fellat et al | 2003 | French | Research | maternal | Maternal Morbidity | 168 |
| Agoub et al | 2005 | English | Research | maternal | Maternal Morbidity | 169 |
| Alami et al | 2006 | English | Research | maternal | Maternal Morbidity | 170 |
| El Bouazzaoui et al | 2010 | English | Case study | maternal | Maternal Morbidity | 171 |
| Bentata et al | 2011 | English | Brief/ short communication/ report | maternal | Maternal Morbidity | 172 |
| Miguil et al | 2011 | French | Research | maternal | Maternal Morbidity | 173 |
| Bentata et al | 2012 | English | Brief/ short communication/ report | maternal | Maternal Morbidity | 174 |
| Bentata et al | 2012 | English | Research | maternal | Maternal Morbidity | 175 |
| Arrayhani et al | 2013 | English | Research | maternal | Maternal Morbidity | 176 |
| Assarag et al | 2013 | English | Research | maternal | Maternal Morbidity | 177 |
| Sasbou et al | 2013 | French | Case study | maternal | Maternal Morbidity | 178 |
| Assarag et al | 2015 | English | Research | maternal | Maternal Morbidity | 179 |
| Elghanmi et al | 2015 | English | Case study | maternal | Maternal Morbidity | 180 |
| Elkhoudri et al | 2015 | English | Research | maternal | Maternal Morbidity | 181 |
| Kabbali et al | 2015 | English | Research | maternal | Maternal Morbidity | 182 |
| Benkirane et al | 2017 | English | Case study | maternal | Maternal Morbidity | 183 |
| Ait Addi et al | 2018 | English | Research | maternal | Maternal Morbidity | 184 |
| Ait Addi et al | 2020 | English | Research | maternal | Maternal Morbidity | 185 |
| Moussaoui et al | 2020 | French | Case study | maternal | Maternal Morbidity | 186 |
| Bouhaddoune et al | 2021 | English | Case study | maternal | Maternal Morbidity | 187 |
| Jabi et al | 2021 | English | Case study | maternal | Maternal Morbidity | 188 |
| Hassine et al | 2022 | French | Case study | maternal | Maternal Morbidity | 189 |
| Manoussi et al | 2022 | French | Research | maternal | Maternal Morbidity | 190 |
| Abouchadi et al | 2013 | English | Research | maternal | Maternal Mortality | 191 |
| Abouchadi et al | 2013 | English | Research | maternal | Maternal Mortality | 192 |
| Abouchadi et al | 2013 | English | Research | maternal | Maternal Mortality | 193 |
| Collaborators GMM | 2016 | English | Research | maternal | Maternal Mortality | 194 |
| Abouchadi et al | 2018 | English | Research | maternal | Maternal Mortality | 195 |
| Nieto-Valvache et al | 2021 | English | Research | maternal | Maternal Mortality | 196 |
| Abouchadi et al | 2022 | English | Research | maternal | Maternal Mortality | 197 |
| Nichols et al | 2022 | English | Research | Both | Maternal Mortality | 198 |
| Okafor et al | 2022 | English | Research | maternal | Maternal Mortality | 199 |
| Sahel et al | 2001 | French | Research | maternal | Maternal Near-Miss | 200 |
| Richard et al | 2003 | French | Research | maternal | Maternal Near-Miss | 201 |
| Filippi et al | 2004 | English | Research | maternal | Maternal Near-Miss | 202 |
| Filippi et al | 2005 | English | Research | maternal | Maternal Near-Miss | 203 |
| Assarag et al | 2015 | English | Research | maternal | Maternal Near-Miss | 204 |
| Temmar et al | 2005 | English | Perspective | Both | Midwifery | 205 |
| Temmar et al | 2006 | English | Perspective | Both | Midwifery | 206 |
| Van Lerberghe et al | 2014 | English | Research | Both | Midwifery | 207 |
| Abou Malham et al | 2015 | English | Research | maternal | Midwifery | 208 |
| Abou-Malham et al | 2015 | English | Research | Both | Midwifery | 209 |
| Martínez-Linares et al | 2019 | English | Perspective | Both | Midwifery | 210 |
| Cadée et al | 2021 | English | Research | Both | Midwifery | 211 |
| Ghafili et al | 2022 | English | Research | maternal | Midwifery | 212 |
| Louazi et al | 2022 | English | Research | maternal | Midwifery | 213 |
| Rouahi et al | 2022 | English | Research | Both | Midwifery | 214 |
| Khadir et al | 2009 | French | Letter | infants | Newborn/Neonatal Health | 215 |
| Lahmiti et al | 2009 | English | Case study | infants | Newborn/Neonatal Health | 216 |
| Kabiri et al | 2010 | English | Case study | infants | Newborn/Neonatal Health | 217 |
| Maoulainine et al | 2012 | French | Research | infants | Newborn/Neonatal Health | 218 |
| Mekkaoui et al | 2012 | English | Research | infants | Newborn/Neonatal Health | 219 |
| Touhami Elouazzani et al | 2012 | French | Research | infants | Newborn/Neonatal Health | 220 |
| Younous et al | 2012 | English | Case study | infants | Newborn/Neonatal Health | 221 |
| Chemsi et al | 2013 | French | Research | infants | Newborn/Neonatal Health | 222 |
| Elguazzar et al | 2013 | French | Research | infants | Newborn/Neonatal Health | 223 |
| Oulmaati et al | 2013 | French | Research | infants | Newborn/Neonatal Health | 224 |
| Chahid et al | 2014 | French | Research | Both | Newborn/Neonatal Health | 225 |
| Fadil et al | 2014 | French | Case study | infants | Newborn/Neonatal Health | 226 |
| Ratbi et al | 2014 | English | Case study | infants | Newborn/Neonatal Health | 227 |
| Noureddine et al | 2015 | English | Letter | Both | Newborn/Neonatal Health | 228 |
| Hassoune et al | 2015 | French | Letter | maternal | Newborn/Neonatal Health | 229 |
| Hassoune et al | 2015 | French | Research | infants | Newborn/Neonatal Health | 230 |
| Boubkraoui et al | 2016 | French | Research | Both | Newborn/Neonatal Health | 231 |
| El Hasbaoui et al | 2017 | English | Case study | infants | Newborn/Neonatal Health | 232 |
| Maoulainine et al | 2017 | English | Research | infants | Newborn/Neonatal Health | 233 |
| Saiad et al | 2017 | English | Letter | infants | Newborn/Neonatal Health | 234 |
| Radouani et al | 2017 | English | Research | infants | Newborn/Neonatal Health | 235 |
| Abdellaoui et al | 2018 | English | Case study | infants | Newborn/Neonatal Health | 236 |
| Barkat et al | 2019 | French | Research | infants | Newborn/Neonatal Health | 237 |
| El Qadiry et al | 2021 | English | Case study | infants | Newborn/Neonatal Health | 238 |
| Elfane et al | 2021 | English | Research | Both | Newborn/Neonatal Health | 239 |
| Elaabsi et al | 2022 | English | Research | infants | Newborn/Neonatal Health | 240 |
| Elfane et al | 2022 | English | Research | infants | Newborn/Neonatal Health | 241 |
| Imad et al | 2022 | English | Case study | infants | Newborn/Neonatal Health | 242 |
| Belgnaoui et al | 2006 | English | Research | maternal | Nutrition | 243 |
| Zaida et al | 2006 | French | Research | Both | Nutrition | 244 |
| Zaida et al | 2006 | English | Research | infants | Nutrition | 245 |
| El Hamdouchi et al | 2013 | English | Research | maternal | Nutrition | 246 |
| Mochhoury et al | 2013 | English | Research | maternal | Nutrition | 247 |
| Karamanos et al | 2014 | English | Research | maternal | Nutrition | 248 |
| Coullin et al | 2015 | English | Research | maternal | Nutrition | 249 |
| Loudyi et al | 2016 | English | Research | infants | Nutrition | 250 |
| Stinca et al | 2017 | English | Research | Both | Nutrition | 251 |
| Lifschitz et al | 2018 | English | Research | infants | Nutrition | 252 |
| Ouzennou et al | 2018 | French | Research | maternal | Nutrition | 253 |
| Ouzennou et al | 2019 | English | Research | maternal | Nutrition | 254 |
| Atalhi et al | 2020 | English | Research | maternal | Nutrition | 255 |
| Kancherla et al | 2021 | English | Research | infants | Nutrition | 256 |
| Taoudi et al | 2021 | English | Research | Both | Nutrition | 257 |
| Rami et al | 2022 | English | Research | maternal | Nutrition | 258 |
| Bentama et al | 2012 | French | Research | infants | Other Non-Respiratory Viruses | 259 |
| Mansouri et al | 2019 | English | Case study | infants | Other Non-Respiratory Viruses | 260 |
| Loaghzaoui Boukaidi et al | 2001 | French | Research | maternal | Parasitic Disease | 261 |
| El Mansouri et al | 2007 | French | Research | maternal | Parasitic Disease | 262 |
| Laboudi et al | 2009 | French | Letter | maternal | Parasitic Disease | 263 |
| Rachad et al | 2012 | French | Case study | maternal | Parasitic Disease | 264 |
| Laboudi et al | 2017 | English | Research | maternal | Parasitic Disease | 265 |
| Tlamcani et al | 2017 | English | Research | maternal | Parasitic Disease | 266 |
| Mouttaki et al | 2018 | English | Case study | infants | Parasitic Disease | 267 |
| Ouzennou et al | 2019 | English | Research | maternal | Parasitic Disease | 268 |
| Tazi et al | 2019 | French | Letter | infants | Parasitic Disease | 269 |
| Hoummadi et al | 2020 | English | Research | maternal | Parasitic Disease | 270 |
| Laboudi et al | 2020 | English | Research | maternal | Parasitic Disease | 271 |
| Ait Hamou et al | 2021 | English | Research | maternal | Parasitic Disease | 272 |
| Laboudi et al | 2021 | English | Research | maternal | Parasitic Disease | 273 |
| Felin et al | 2022 | English | Review | Both | Parasitic Disease | 274 |
| Felin et al | 2022 | English | Review | Both | Parasitic Disease | 275 |
| Hattoufi et al | 2022 | English | Research | maternal | Parasitic Disease | 276 |
| El Youssoufi et al | 2007 | French | Case study | maternal | Preeclampsia/Eclampsia | 277 |
| Sabiri et al | 2007 | French | Research | maternal | Preeclampsia/Eclampsia | 278 |
| Araqi-Houssaini et al | 2011 | French | Research | maternal | Preeclampsia/Eclampsia | 279 |
| Mamouni et al | 2012 | French | Case study | maternal | Preeclampsia/Eclampsia | 280 |
| Zidouh et al | 2012 | English | Letter | maternal | Preeclampsia/Eclampsia | 281 |
| Djoubairou et al | 2014 | English | Case study | maternal | Preeclampsia/Eclampsia | 282 |
| Handor et al | 2014 | English | Case study | maternal | Preeclampsia/Eclampsia | 283 |
| Bentata et al | 2015 | English | Brief/ short communication/ report | maternal | Preeclampsia/Eclampsia | 284 |
| Benfateh et al | 2018 | English | Research | maternal | Preeclampsia/Eclampsia | 285 |
| Rebahi et al | 2018 | English | Research | maternal | Preeclampsia/Eclampsia | 286 |
| Soumani et al | 2000 | French | Research | Both | Pregnancy | 287 |
| Berrada et al | 2001 | French | Case study | maternal | Pregnancy | 288 |
| Benchikhi et al | 2002 | French | Research | maternal | Pregnancy | 289 |
| Kadri et al | 2007 | English | Research | maternal | Pregnancy | 290 |
| Ezzouine et al | 2008 | French | Letter | Both | Pregnancy | 291 |
| Boufettal et al | 2012 | French | Research | maternal | Pregnancy | 292 |
| Boufettal et al | 2012 | French | Research | maternal | Pregnancy | 293 |
| Oukkache et al | 2012 | English | Case study | maternal | Pregnancy | 294 |
| El Hamdani et al | 2013 | French | Research | maternal | Pregnancy | 295 |
| Hicham et al | 2013 | English | Case study | maternal | Pregnancy | 296 |
| Mansouri et al | 2013 | English | Case study | maternal | Pregnancy | 297 |
| Slimani et al | 2014 | English | Case study | maternal | Pregnancy | 298 |
| El Ammouri et al | 2015 | French | Research | maternal | Pregnancy | 299 |
| Samali et al | 2017 | English | Case study | maternal | Pregnancy | 300 |
| Yasmina et al | 2017 | French | Research | Both | Pregnancy | 301 |
| Bakrim et al | 2018 | English | Research | maternal | Pregnancy | 302 |
| Hassane et al | 2018 | French | Research | maternal | Pregnancy | 303 |
| El Jaouhari et al | 2019 | English | Case study | maternal | Pregnancy | 304 |
| Guennoun et al | 2019 | English | Case study | maternal | Pregnancy | 305 |
| Slaoui et al | 2019 | English | Case study | maternal | Pregnancy | 306 |
| Zaidi et al | 2019 | French | Case study | Both | Pregnancy | 307 |
| Elkoundi et al | 2020 | English | Case study | maternal | Pregnancy | 308 |
| Soule et al | 2020 | English | Case study | maternal | Pregnancy | 309 |
| Chergaoui et al | 2022 | English | Research | maternal | Pregnancy | 310 |
| Mahi et al | 2002 | French | Case study | Both | Pregnancy--ectopic | 311 |
| Siati et al | 2019 | English | Case study | maternal | Pregnancy--ectopic | 312 |
| Elmiski et al | 2021 | English | Case study | maternal | Pregnancy--ectopic | 313 |
| Bousb et al | 2022 | English | Case study | maternal | Pregnancy--ectopic | 314 |
| Laghzaoui Boukaidi et al | 2002 | French | Case study | maternal | Pregnancy--heterotopic | 315 |
| Guennoun et al | 2017 | French | Case study | maternal | Pregnancy--heterotopic | 316 |
| Ouafidi et al | 2021 | English | Case study | maternal | Pregnancy--heterotopic | 317 |
| Boufettal et al | 2011 | French | Research | maternal | Pregnancy--hydatiform mole | 318 |
| Boufettal et al | 2012 | French | Research | maternal | Pregnancy--hydatiform mole | 319 |
| Bousfiha et al | 2012 | English | Case study | maternal | Pregnancy--hydatiform mole | 320 |
| Zohoun et al | 2013 | French | Research | maternal | Pregnancy--hydatiform mole | 321 |
| El Miski et al | 2021 | English | Case study | maternal | Pregnancy--hydatiform mole | 322 |
| Slaoui et al | 2022 | English | Case study | maternal | Pregnancy--hydatiform mole | 323 |
| Gueddari et al | 2014 | French | Research | infants | Public Information/Literacy | 324 |
| Ouasmani et al | 2018 | English | Research | maternal | Public Information/Literacy | 325 |
| Adnane Berdai et al | 2012 | French | Research | Both | Respiratory Virus | 326 |
| Ait Addi et al | 2020 | English | Brief/ short communication/ report | maternal | Respiratory Virus | 327 |
| Hattoufi et al | 2020 | English | Research | infants | Respiratory Virus | 328 |
| Katfy et al | 2020 | English | Research | infants | Respiratory Virus | 329 |
| Aasfara et al | 2021 | English | Case study | maternal | Respiratory Virus | 330 |
| Benlghazi et al | 2021 | French | Research | maternal | Respiratory Virus | 331 |
| Ouahid et al | 2022 | French | Research | Both | Respiratory Virus | 332 |
| Zouini et al | 2010 | French | Research | maternal | Rural/Amazigh | 333 |
| Sebbani et al | 2016 | French | Research | maternal | Rural/Amazigh | 334 |
| Sebbani et al | 2020 | French | Research | maternal | Rural/Amazigh | 335 |
| Sebbani et al | 2020 | English | Research | maternal | Rural/Amazigh | 336 |
| Baayd et al | 2021 | English | Research | maternal | Rural/Amazigh | 337 |
| Belahcen et al | 2014 | English | Research | maternal | Screening for Newborns | 338 |
| Hamzi et al | 2014 | English | Letter | maternal | Screening for Newborns | 339 |
| Oulmaati et al | 2016 | French | Letter | infants | Screening for Newborns | 340 |
| Maniar et al | 2018 | French | Research | infants | Screening for Newborns | 341 |
| El Idrissi Slitine et al | 2020 | English | Research | infants | Screening for Newborns | 342 |
| Hom et al | 2020 | English | Letter | infants | Screening for Newborns | 343 |
| Therrell et al | 2020 | English | Research | infants | Screening for Newborns | 344 |
| Abdesslam et al | 2011 | English | Research | Both | Social Determinants of Health | 345 |
| Boutayeb et al | 2016 | English | Research | Both | Social Determinants of Health | 346 |
| Akseer et al | 2018 | English | Research | Both | Social Determinants of Health | 347 |
| Ricker C et al | 2020 | English | Research | maternal | Social Determinants of Health | 348 |
| Drioui et al | 2021 | English | Research | maternal | Social Determinants of Health | 349 |
| Chandani et al | 2001 | English | Research | maternal | Technology | 350 |
| Bachiri et al | 2018 | English | Research | maternal | Technology | 351 |
| El Hasbaoui et al | 2018 | English | Letter | infants | Technology | 352 |
| Lykins et al | 2018 | English | Research | maternal | Technology | 353 |
| Ouhenach et al | 2020 | English | Case study | maternal | Technology | 354 |
| Kharbouch et al | 2021 | English | Protocol paper | maternal | Technology | 355 |
| Sabir et al | 2022 | English | Research | infants | Technology | 356 |
| AMDD Working Group on Indicators | 2003 | English | Brief/ short communication/ report | maternal | The Healthcare system | 357 |
| Hotchkiss et al | 2005 | English | Working paper | maternal | The Healthcare system | 358 |
| Fauveau et al | 2006 | English | Research | maternal | The Healthcare system | 359 |
| Couillet et al | 2007 | English | Research | maternal | The Healthcare system | 360 |
| Muffler et al | 2007 | English | Research | Both | The Healthcare system | 361 |
| Fernandez et al | 2009 | English | Research | maternal | The Healthcare system | 362 |
| Bennis et al | 2012 | English | Research | maternal | The Healthcare system | 363 |
| Marchal et al | 2013 | English | Perspective | maternal | The Healthcare system | 364 |
| Bartlett et al | 2014 | English | Research | Both | The Healthcare system | 365 |
| Boukhalfa et al | 2016 | English | Research | maternal | The Healthcare system | 366 |
| Witter et al | 2016 | English | Research | maternal | The Healthcare system | 367 |
| Elkhoudri et al | 2017 | English | Research | maternal | The Healthcare system | 368 |
| Witter et al | 2017 | English | Research | maternal | The Healthcare system | 369 |
| Van der Veken et al | 2018 | English | Research | maternal | The Healthcare system | 370 |
| Ouakhzan et al | 2019 | French | Research | maternal | The Healthcare system | 371 |
| Assarag et al | 2020 | English | Research | maternal | The Healthcare system | 372 |
| Hasan et al | 2020 | English | Research | Both | The Healthcare system | 373 |
| Kabakian-Khasholian et al | 2020 | English | Research | Both | The Healthcare system | 374 |
| Ammerdorffer et al | 2021 | English | Research | maternal | The Healthcare system | 375 |
| Bezad et al | 2022 | English | Research | maternal | The Healthcare system | 376 |
| Lekouch et al | 2001 | English | Research | infants | Traditional medicine | 377 |
| Khalki et al | 2010 | English | Research | maternal | Traditional medicine | 378 |
| Achour et al | 2011 | English | Case study | infants | Traditional medicine | 379 |
| Achour et al | 2012 | French | Research | maternal | Traditional medicine | 380 |
| Achour et al | 2012 | French | Case study | Both | Traditional medicine | 381 |
| Khalki et al | 2012 | English | Research | maternal | Traditional medicine | 382 |
| Said et al | 2015 | English | Research | infants | Traditional medicine | 383 |
| Elkhoudri et al | 2016 | English | Research | maternal | Traditional medicine | 384 |
| Oulmaati et al | 2017 | French | Research | infants | Traditional medicine | 385 |
| Teixidor-Toneu et al | 2017 | English | Research | infants | Traditional medicine | 386 |
| El Kamari et al | 2018 | English | Research | infants | Traditional medicine | 387 |
| El Kamari et al | 2018 | English | Research | infants | Traditional medicine | 388 |
| Laadraoui et al | 2018 | English | Research | maternal | Traditional medicine | 389 |
| Taroq et al | 2018 | English | Research | infants | Traditional medicine | 390 |
| Hoummani et al | 2019 | French | Case study | infants | Traditional medicine | 391 |
| Lakhdar et al | 2019 | French | Case study | infants | Traditional medicine | 392 |
| Taroq et al | 2019 | English | Research | infants | Traditional medicine | 393 |
| Eddouks et al | 2020 | English | Research | maternal | Traditional medicine | 394 |
| Slighoua et al | 2020 | English | Research | maternal | Traditional medicine | 395 |
| Kamel et al | 2022 | English | Research | maternal | Traditional medicine | 396 |
| Caidi et al | 2009 | English | Research | maternal | Vaccination | 397 |
| Belefquih et al | 2013 | English | Brief/ short communication/ report | maternal | Vaccination | 398 |
| Lohiniva et al | 2014 | English | Research | maternal | Vaccination | 399 |
| Sbiti et al | 2016 | French | Research | maternal | Vaccination | 400 |
| Messaoudi et al | 2020 | French | Research | infants | Vaccination | 401 |
| Zahir et al | 2020 | English | Research | maternal | Vaccination | 402 |
