# Supplementary material for: Mapping maternal and infant health in Morocco: A global scoping review of themes, gaps, and the "unseen" in the published health research literature, 2000–2022
Source: PLOS Glob Public Health. 2024 Jul 18;4(7):e0003488. doi: 10.1371/journal.pgph.0003488 (PMC11257357; doi:10.1371/journal.pgph.0003488)
Supplement: S5 Table — (DOCX) [file pgph.0003488.s013.docx]

Table S5. Hospitals, Institutions, and Other Locations where research was conducted by time period

| **Hospital** | **2000-2011** | | **2012-2022** | | **Total** | |
| --- | --- | --- | --- | --- | --- | --- |
|  | **n** | **%** | **n** | **%** | **n** | **%** |
| Al Farabi Regional Hospital | 1 | 1.1% | 2 | 0.6% | 3 | 0.7% |
| Al Ghassani Hospital | 1 | 1.1% | 0 | 0.0% | 1 | 0.2% |
| Benslimane Military Hospital | 0 | 0.0% | 1 | 0.3% | 1 | 0.2% |
| Cheikh Khalifa International University Hospital | 0 | 0.0% | 1 | 0.3% | 1 | 0.2% |
| CHU Caen (France) | 1 | 1.1% | 0 | 0.0% | 1 | 0.2% |
| CHU Hassan II Fes, Maroc | 2 | 2.2% | 27 | 8.7% | 29 | 7.2% |
| CHU Ibn Sina Rabat | 12 | 12.9% | 56 | 18.1% | 68 | 16.9% |
| CHU Ibn-Rochd Casablanca | 22 | 23.7% | 32 | 10.4% | 54 | 13.4% |
| CHU Mohamed VI University Hospital - Marrakesh | 2 | 2.2% | 23 | 7.4% | 25 | 6.2% |
| CHU Mohammed VI Oujda | 0 | 0.0% | 6 | 2.0% | 6 | 1.4% |
| CHU Tangier-Tetouan-Al Hoceima University Hospital | 0 | 0.0% | 1 | 0.3% | 1 | 0.2% |
| Elidrissi Hospital - Kenitra | 0 | 0.0% | 1 | 0.3% | 1 | 0.2% |
| El-Jadida Provincial Hospital | 0 | 0.0% | 2 | 0.6% | 2 | 0.5% |
| Hassan II Hospital - Benslimane | 0 | 0.0% | 1 | 0.3% | 1 | 0.2% |
| Hassan II Hospital - Settat | 0 | 0.0% | 1 | 0.3% | 1 | 0.2% |
| Hopital Militaire Moulay Ismail Meknes | 0 | 0.0% | 1 | 0.3% | 1 | 0.2% |
| Hospital Amizmiz | 1 | 1.1% | 0 | 0.0% | 1 | 0.2% |
| Military Hospital Mohammed V Rabat | 2 | 2.2% | 8 | 2.6% | 10 | 2.4% |
| Moulay Abdellah Provincial Hospital | 0 | 0.0% | 1 | 0.3% | 1 | 0.2% |
| Moulay Ali Cherif Provincial Hospital | 0 | 0.0% | 1 | 0.3% | 1 | 0.2% |
| Pediatric Hospital Al Firdaous | 0 | 0.0% | 1 | 0.3% | 1 | 0.2% |
| Provincial Hospital Center of M'diq | 0 | 0.0% | 1 | 0.3% | 1 | 0.2% |
| Provincial Hospital of Amizmiz | 0 | 0.0% | 1 | 0.3% | 1 | 0.2% |
| Provincial Hospital of Mohammedia | 0 | 0.0% | 2 | 0.6% | 2 | 0.5% |
| Regional Hospital of Oujda | 0 | 0.0% | 1 | 0.3% | 1 | 0.2% |
| Sidi Lahcen | 0 | 0.0% | 1 | 0.3% | 1 | 0.2% |
| Teaching Hospital, Sidi Mohamed Ben Abdellah University | 0 | 0.0% | 2 | 0.6% | 2 | 0.5% |
| Tiznit Provincial Hospital Center | 0 | 0.0% | 1 | 0.3% | 1 | 0.2% |
| Demographic Health Survey | 1 | 1.1% | 1 | 0.3% | 2 | 0.5% |
| Government/Ngo/Academic | 8 | 8.6% | 25 | 8.1% | 33 | 8.2% |
| Private Practice | 0 | 0.0% | 1 | 0.3% | 1 | 0.2% |
| Other Type of Institution | 0 | 0.0% | 4 | 1.3% | 4 | 1.0% |
| Multiple | 4 | 4.3% | 22 | 7.1% | 26 | 6.5% |
| Not Reported | 18 | 19.4% | 47 | 15.2% | 65 | 16.2% |
| Not Applicable | 18 | 19.4% | 34 | 11.0% | 52 | 12.9% |
