# Supplementary material for: Mapping maternal and infant health in Morocco: A global scoping review of themes, gaps, and the "unseen" in the published health research literature, 2000–2022
Source: PLOS Glob Public Health. 2024 Jul 18;4(7):e0003488. doi: 10.1371/journal.pgph.0003488 (PMC11257357; doi:10.1371/journal.pgph.0003488)
Supplement: S6 Table — (DOCX) [file pgph.0003488.s014.docx]

Table S6. Frequency of articles by primary theme, population focus, and time period

| **Theme** | **2000-2011** | | | | **2012-2022** | | | | **Overall** | | | |
| --- | --- | --- | --- | --- | --- | --- | --- | --- | --- | --- | --- | --- |
|  | **Both** | **Infant focus** | **Maternal focus** | **Total** | **Both** | **Infant focus** | **Maternal focus** | **Total** | **Both** | **Infant focus** | **Maternal focus** | **Total** |
| **Abortion** |  |  | 3 | **3** |  |  | 7 | **7** | **0** | **0** | **10** | **10** |
| **AIDS/ STI** |  |  | 1 | **1** |  |  |  |  | **0** | **0** | **1** | **1** |
| **Bacterial Infection** |  | 9 | 1 | **10** | 1 | 16 | 4 | **21** | **1** | **25** | **5** | **31** |
| **Birth** |  |  | 9 | **9** |  |  | 10 | **10** | **0** | **0** | **19** | **19** |
| **Breastfeeding** | 1 |  | 1 | **2** | 3 | 1 | 11 | **15** | **4** | **1** | **12** | **17** |
| **Cancer** |  |  | 1 | **1** | 1 |  | 4 | **5** | **1** | **0** | **5** | **6** |
| **Diabetes** |  |  | 1 | **1** |  |  | 7 | **7** | **0** | **0** | **8** | **8** |
| **Environment** |  | 3 |  | **3** | 1 |  | 5 | **6** | **1** | **3** | **5** | **9** |
| **Family Planning** |  |  | 9 | **9** |  |  | 4 | **4** | **0** | **0** | **13** | **13** |
| **Genetics** |  | 4 | 1 | **5** |  | 13 | 5 | **18** | **0** | **17** | **6** | **23** |
| **Gynecology** |  |  | 1 | **1** |  |  | 2 | **2** | **0** | **0** | **3** | **3** |
| **Infant Morbidity** |  | 1 |  | **1** |  | 15 | 4 | **19** | **0** | **16** | **4** | **20** |
| **Infant Mortality** |  |  | 1 | **1** | 1 | 2 |  | **3** | **1** | **2** | **1** | **4** |
| **Infant Near-Miss** |  |  |  |  |  | 1 |  | **1** | **0** | **1** | **0** | **1** |
| **Legal** |  |  |  |  |  |  | 1 | **1** | **0** | **0** | **1** | **1** |
| **Maternal Morbidity** |  |  | 7 | **7** |  |  | 17 | **17** | **0** | **0** | **24** | **24** |
| **Maternal Mortality** |  |  |  |  | 1 |  | 8 | **9** | **1** | **0** | **8** | **9** |
| **Maternal Near-Miss** |  |  | 4 | **4** |  |  | 1 | **1** | **0** | **0** | **5** | **5** |
| **Midwifery** | 2 |  |  | **2** | 5 |  | 3 | **8** | **7** | **0** | **3** | **10** |
| **Newborn/Neonatal Health** |  | 3 |  | **3** | 4 | 20 | 1 | **25** | **4** | **23** | **1** | **28** |
| **Nutrition** | 1 | 1 | 1 | **3** | 2 | 3 | 8 | **13** | **3** | **4** | **9** | **16** |
| **Other Non-Respiratory Viruses** |  |  |  |  |  | 2 |  | **2** | **0** | **2** | **0** | **2** |
| **Parasitic Disease** |  |  | 3 | **3** | 2 | 2 | 9 | **13** | **2** | **2** | **12** | **16** |
| **Preeclampsia/Eclampsia** |  |  | 3 | **3** |  |  | 7 | **7** | **0** | **0** | **10** | **10** |
| **Pregnancy** | 2 |  | 3 | **5** | 2 |  | 17 | **19** | **4** | **0** | **20** | **24** |
| **Pregnancy--ectopic** | 1 |  |  | **1** |  |  | 3 | **3** | **1** | **0** | **3** | **4** |
| **Pregnancy--heterotopic** |  |  | 1 | **1** |  |  | 2 | **2** | **0** | **0** | **3** | **3** |
| **Pregnancy--hydatiform mole** |  |  | 1 | **1** |  |  | 5 | **5** | **0** | **0** | **6** | **6** |
| **Public Information/Literacy** |  |  |  |  |  | 1 | 1 | **2** | **0** | **1** | **1** | **2** |
| **Respiratory Virus** |  |  |  |  | 2 | 2 | 3 | **7** | **2** | **2** | **3** | **7** |
| **Rural/Amazigh** |  |  | 1 | **1** |  |  | 4 | **4** | **0** | **0** | **5** | **5** |
| **Screening for Newborns** |  |  |  |  |  | 5 | 2 | **7** | **0** | **5** | **2** | **7** |
| **Social Determinants of Health** | 1 |  |  | **1** | 2 |  | 2 | **4** | **3** | **0** | **2** | **5** |
| **Technology** |  |  | 1 | **1** |  | 2 | 4 | **6** | **0** | **2** | **5** | **7** |
| **The Healthcare system** | 1 |  | 5 | **6** | 3 |  | 11 | **14** | **4** | **0** | **16** | **20** |
| **Traditional medicine** |  | 2 | 1 | **3** | 1 | 9 | 7 | **17** | **1** | **11** | **8** | **20** |
| **Vaccination** |  |  | 1 | **1** |  | 1 | 4 | **5** | **0** | **1** | **5** | **6** |
| **Grand Total** | 9 | 23 | 61 | **93** | 31 | 95 | 183 | **309** | **40** | **118** | **244** | **402** |
