# Supplementary material for: Mapping maternal and infant health in Morocco: A global scoping review of themes, gaps, and the "unseen" in the published health research literature, 2000–2022
Source: PLOS Glob Public Health. 2024 Jul 18;4(7):e0003488. doi: 10.1371/journal.pgph.0003488 (PMC11257357; doi:10.1371/journal.pgph.0003488)
Supplement: S7 Table — (DOCX) [file pgph.0003488.s015.docx]

Table S7. Summary statistics of sub-group MIH articles

|  |  | **2000-2011 (n=35)** | | **2012-2022 (n=93)** | | **Total (n=128)** | |
| --- | --- | --- | --- | --- | --- | --- | --- |
|  |  | **n** | **%** | **n** | **%** | **n** | **%** |
| Stage | Both | 5 | 14.3% | 14 | 15.1% | 19 | 14.8% |
|  | Infant theme | 21 | 60.0% | 65 | 69.9% | 86 | 67.2% |
|  | Maternal theme | 9 | 25.7% | 14 | 15.1% | 23 | 18.0% |
| Language | English | 24 | 68.6% | 77 | 82.8% | 101 | 78.9% |
|  | French | 11 | 31.4% | 15 | 16.1% | 26 | 20.3% |
|  | German (English translation available) | 0 | 0.0% | 1 | 1.1% | 1 | 0.8% |
| Hospital vs community-based sample | Both | 0 | 0.0% | 2 | 2.2% | 2 | 1.6% |
|  | Community | 6 | 17.1% | 8 | 8.6% | 14 | 10.9% |
|  | Hospital | 28 | 80.0% | 82 | 88.2% | 110 | 85.9% |
|  | Not applicable | 1 | 2.9% | 1 | 1.1% | 2 | 1.6% |
| Level | Global | 1 | 2.9% | 6 | 6.5% | 7 | 5.5% |
|  | National | 12 | 34.3% | 19 | 20.4% | 31 | 24.2% |
|  | Regional/ Local | 21 | 60.0% | 68 | 73.2% | 89 | 69.6% |
|  | Multiple levels | 1 | 2.9% | 0 | 0.0% | 1 | 0.8% |
| % study population that is comprised of mothers or infants | 0-25 | 11 | 31.4% | 35 | 37.6% | 46 | 35.9% |
|  | 26-50 | 5 | 14.3% | 20 | 21.5% | 25 | 19.5% |
|  | 51-75 | 6 | 17.1% | 9 | 9.7% | 15 | 11.7% |
|  | 76-100 | 1 | 2.9% | 5 | 5.4% | 6 | 4.7% |
|  | Not reported/ Not applicable | 12 | 34.3% | 24 | 25.9% | 36 | 28.1% |
| Primary Theme | AIDS/STD | 3 | 8.6% | 2 | 2.2% | 5 | 3.9% |
|  | Bacterial infection | 4 | 11.4% | 24 | 25.8% | 28 | 21.9% |
|  | Cancer | 1 | 2.9% | 2 | 2.2% | 3 | 2.3% |
|  | Diabetes | 0 | 0.0% | 2 | 2.2% | 2 | 1.6% |
|  | Environment | 5 | 14.3% | 6 | 6.5% | 11 | 8.6% |
|  | Family planning | 0 | 0.0% | 1 | 1.1% | 1 | 0.8% |
|  | Genetics | 6 | 17.1% | 15 | 16.1% | 21 | 16.4% |
|  | Gynecology | 0 | 0.0% | 1 | 1.1% | 1 | 0.8% |
|  | Infant morbidity | 1 | 2.9% | 5 | 5.4% | 6 | 4.7% |
|  | Legal | 1 | 2.9% | 0 | 0.0% | 1 | 0.8% |
|  | Maternal morbidity | 0 | 0.0% | 1 | 1.1% | 1 | 0.8% |
|  | Nutrition | 2 | 5.7% | 1 | 1.1% | 3 | 2.3% |
|  | Other Non-Respiratory Viruses | 2 | 5.7% | 3 | 3.2% | 5 | 3.9% |
|  | Parasitic Disease | 2 | 5.7% | 6 | 6.5% | 8 | 6.3% |
|  | Provider | 1 | 2.9% | 1 | 1.1% | 2 | 1.6% |
|  | Respiratory Virus | 2 | 5.7% | 10 | 10.8% | 12 | 9.4% |
|  | Rural/Amazigh | 0 | 0.0% | 1 | 1.1% | 1 | 0.8% |
|  | Technology | 0 | 0.0% | 1 | 1.1% | 1 | 0.8% |
|  | The Healthcare system | 3 | 8.6% | 7 | 7.5% | 10 | 7.8% |
|  | Traditional medicine | 0 | 0.0% | 2 | 2.2% | 2 | 1.6% |
|  | Vaccination | 2 | 5.7% | 2 | 2.2% | 4 | 3.1% |
