# Supplementary material for: Mapping maternal and infant health in Morocco: A global scoping review of themes, gaps, and the "unseen" in the published health research literature, 2000–2022
Source: PLOS Glob Public Health. 2024 Jul 18;4(7):e0003488. doi: 10.1371/journal.pgph.0003488 (PMC11257357; doi:10.1371/journal.pgph.0003488)
Supplement: S1 Text — (DOCX) [file pgph.0003488.s016.docx]

Text S1. Bibliography of full-text MIH articles organized by primary theme

**Abortion** (10)

1. Hessini L. Abortion and Islam: Policies and practice in the Middle East and North Africa. Reprod Health Matters. 2007;15(29):75-84. 10.1016/S0968-8080(06)29279-6.

2. Abidi K, Himdi B, Cherradi N, Lamalmi N, Alhamany Z, Zeggwagh A, et al. Myocardial lysis in a fetus induced by maternal paraphenylenediamine poisoning following an intentional ingestion to induce abortion. Hum Exp Toxicol. 2008;27(5):435-8. 10.1177/0960327108092288.

3. Belhouss A, Ait Boughima F, Benyaich H, Boufettal H, Samouh N. [The forensic aspects of the abortion in Morocco]. Les aspects médicolégaux de l'avortement au Maroc. Rev de Med Leg. 2011;2(4):170-3. 10.1016/j.medleg.2011.08.001.

4. Berdai M, Labib S, Harandou M. Peganum harmala L. intoxication in a pregnant woman. Case Rep Emerg Med. 2014;2014:783236. 10.1155/2014/783236.

5. Chraibi C. [Unwanted pregnancies kill in Morocco]. Au Maroc, les grossesses non désirées tuent. Gynecol Obstet Fertil. 2014;42(9):559-60. 10.1016/j.gyobfe.2014.07.005.

6. Foster A, Wynn L, Trussell J. Evidence of global demand for medication abortion information: An analysis of www.medicationabortion.com. Contraception. 2014;89(3):174-80. 10.1016/j.contraception.2013.05.005.

7. Laghzaoui O. [Inventory of unsafe abortions: retrospective study of 451 cases treated in Moulay Ismail Military Hospital of instruction, Meknes, Morocco]. Avortements non médicalisés: état des lieux à travers une étude rétrospective de 451 cas traités à l'hôpital militaire d'instruction Moulay Ismail Meknès, Maroc. Pan Afr Med J. 2016;25(24):83. 10.11604/pamj.2016.24.83.8624.

8. Capelli I. Non-marital pregnancies and unmarried women's search for illegal abortion in Morocco. Health Hum Rights. 2019;21(2):33-45.

9. Ghizlane E, Manal M, Ines H, Soufiane D, Moussa L, Houssam B, et al. Fatal poisoning of pregnant women by peganum harmala L.: A case reports. Ann Med Surg (Lond). 2021;68:102649. 10.1016/j.amsu.2021.102649.

10. Mourchid A, Bakass F. [Estimating induced abortion in Morocco. A comparative approach]. Essai d’estimation de la prévalence de l’avortement provoqué au Maroc. Approche comparative. Sante Publique. 2022;34(1):131-40. 10.3917/spub.221.0131.

**AIDS** (1)

11. Chakib A, Laghzaoui B, M, Najib J, Aderdour M, Himmich H. [Pregnancy and AIDS. Report of 9 cases]. Grossesse et SIDA. À propos de 9 cas. Tunis Med. 2001;79(10):530-5.

**Bacterial Infection** (31)

12. Aitmhand R, Moustaoui N, Belabbes H, Elmdaghri N, Benbachir M. Serotypes and antimicrobial susceptibility of group B streptococcus isolated from neonates in Casablanca. Scand J Infect Dis. 2000;32(3):339-40. 10.1080/00365540050166108.

13. Benomar S, Nejjari N, Lahbabi M. [Neonatal listeria infection is very rare in Morocco]. Listériose néonatale: une infection exceptionnelle au Maroc. Arch Pediatr. 2000;7(4):428. 10.1016/S0929-693X%2800%2988844-2.

14. Nejjari N, Allali B, Bouharrou A, Habzi A, Najdi T, Lahbabi S, et al. Neonatal tetanus in Casablanca. Tétanos néonatal à Casablanca. Med Mal Infect. 2002;32(5):223-7. 10.1016/S0399-077X%2802%2900360-8.

15. World Health Organization (WHO). Validation of neonatal tetanus elimination, Morocco, 2002. Wkly Epidemiol Rec. 2002;77(39):325-8.

16. Nejjari N, Zerhouni F, Bouharrou A, Habzi A, Najdi T, Lahbabi M, et al. [Nosocomial infections, caused by acinetobacter neonatology care unit of children's hospital in Casablanca]. Infections nosocomiales à Acinetobacter expérience du service de néonatologie de Casablanca. Tunis Med. 2003;81(2):121-5.

17. Ailal F, Bousfiha A, Jouhadi Z, Adnane F, Abid A. [Forty-one pediatric cases of non-typhoidal salmonellosis]. Les salmonelloses non typhoïdiques chez l'enfant: à propos de 41 cas. Med Mal Infect. 2004;34(5):206-9. 10.1016/j.medmal.2004.02.004.

18. Balaka B, Bonkoungou P, Sqalli M, Bambara M, Millogo A, Agbèrè A. [Comparative study of neonatal bacterial meningitis in Lome, Bobo-Dioulasso, Casablanca and Lyon]. Analyse comparative des méningites bactériennes néonatales à Lomé, Bobo-Dioulasso, Casablanca et Lyon. Bull Soc Pathol Exot. 2004;97(2):131-4.

19. Oulahiane N, Laboudi A, Kabiri M, Ech-Cherif El Kettani S, El Haddoury M, Alaoui I. [Neonatal tetanus: Epidemiological, clinical and therapeutic aspects. Concerning 34 cases]. Le tétanos néonatal : aspects épidémiologiques, cliniques et thérapeutiques. À propos de 34 cas. J de Pediatrie et de Pueric. 2005;18(1):38-43. 10.1016/j.jpp.2004.12.003.

20. Benchellal M, Skalli M, Rouahi N, Biognach H, Rjafallah A, Rhrab B, et al. [Study of the immunity to tetanus in parturient women during the year 2007 in the Maternity of Souissi in Rabat]. Étude de l’immunité antitétanique chez les femmes enceintes durant l’année 2007 au service de maternité Souissi de Rabat. Immuno-anal Biol Spe. 2009;24(1):20-3. 10.1016/j.immbio.2008.07.008.

21. Rosenthal V, Lynch P, Jarvis W, Khader I, Richtmann R, Jaballah N, et al. Socioeconomic impact on device-associated infections in limited-resource neonatal intensive care units: findings of the INICC. Infection. 2011;39(5):439-50. 10.1007/s15010-011-0136-2.

22. Chemsi M, Habzi A, Harrak A, Benomar S. [Performance of procalcitonin diagnosis of maternofetal infection]. Performances de la procalcitonine dans le diagnostic de l’infection maternofœtale. J de Pediatrie et de Pueric. 2012;25(4):185-92. 10.1016/j.jpp.2012.03.009.

23. Rosenthal V, Rodríguez-Calderón M, Rodríguez-Ferrer M, Singhal T, Pawar M, Sobreyra-Oropeza M, et al. Findings of the International Nosocomial Infection Control Consortium (INICC), Part II: Impact of a Multidimensional Strategy to Reduce Ventilator-Associated Pneumonia in Neonatal Intensive Care Units in 10 Developing Countries. Infect Control Hosp Epidemiol. 2012;33(7):704-10. 10.1086/666342.

24. Aseri N, Kabiri M, Razine R, Mrabet M, Kharbach A, El Amrani S, et al. [CRP value in managing the bacterial maternofetal infection]. Intérêt de la C-réactive protéine dans la prise en charge des infections bactériennes maternofœtales. J de Pediatrie et de Pueric. 2014;27(1):9-15. 10.1016/j.jpp.2013.10.008.

25. Maoulainine F, Elidrissi N, Chkil G, Abba F, Soraa N, Chabaa L, et al. [Epidemiology of nosocomial bacterial infection in neonatal intensive care unit in Morocco]. Épidémiologie de l'infection nosocomiale bactérienne dans un service de réanimation néonatal marocain. Arch Pediatr. 2014;21(9):938-43. 10.1016/j.arcped.2014.04.033.

26. Ballén V, Sáez E, Benmessaoud R, Houssain T, Alami H, Barkat A, et al. First report of a Klebsiella pneumoniae ST466 strain causing neonatal sepsis harbouring the blaCTX-M-15 gene in Rabat, Morocco. FEMS Microbiol Lett. 2015;362(1):1-4. 10.1093/femsle/fnu026.

27. Chemsi M, Benomar S. [Early-onset neonatal sepsis]. Infections bactériennes néonatales précoces. J de Pediatrie et de Pueric. 2015;28(1):29-37. 10.1016/j.jpp.2014.10.005.

28. Elatiqi S, Chemsi M, Lehlimi M, Habzi A, Benomar S. [Maternal-fetal infections due to Streptococcus pneumoniae]. Infection maternofœtale à Streptococcus pneumoniae. J de Pediatrie et de Pueric. 2015;28(6):271-5. 10.1016/j.jpp.2015.06.006.

29. Bassir A, Dhibou H, Farah M, Mohamed L, Amal A, Nabila S, et al. [Vaginal colonization by group B streptococcus among pregnant women in the region of Marrakech]. Portage vaginal du streptocoque du groupe B chez la femme enceinte au niveau de la région de Marrakech. Pan Afr Med J. 2016;23:107. 10.11604/pamj.2016.23.107.9047.

30. Chabah M, Chemsi M, Zerouali K, Alloula O, Lehlimi M, Habzi A, et al. Healthcare-associated infections due to carbapenemase-producing Enterobacteriaceae: Bacteriological profile and risk factors. Med Mal Infect. 2016;46(3):157-62. 10.1016/j.medmal.2015.12.015.

31. Sáez-López E, Cossa A, Benmessaoud R, Madrid L, Moraleda C, Villanueva S, et al. Characterization of vaginal Escherichia coli isolated from pregnant women in two different African sites. PLoS One. 2016;11(7):e0158695. 10.1371/journal.pone.0158695.

32. Arhoune B, Oumokhtar B, Hmami F, Barguigua A, Timinouni M, El Fakir S, et al. Rectal carriage of extended-spectrum beta-lactamase- and carbapenemase-producing Enterobacteriaceae among hospitalised neonates in a neonatal intensive care unit in Fez, Morocco. J Glob Antimicrob Resist. 2017;8:90-6. 10.1016/j.jgar.2016.11.004.

33. Chemsi M, Elmasbahi F, Skali Lami A, Lehlimi M, Habzi A, Benomar S. [The decision to perform of lumbar puncture in early-onset neonatal sepsis]. La ponction lombaire dans l’infection néonatale bactérienne précoce : performance et décision. J de Pediatrie et de Pueric. 2018;31(1):27-33. 10.1016/j.jpp.2017.11.002.

34. Daoudi A, Benaoui F, El Idrissi Slitine N, Soraa N, Rabou Maoulainine F. An outbreak of Serratia marcescens in a Moroccan neonatal intensive care unit. Adv Med. 2018;2018:4867134. 10.1155/2018/4867134.

35. Moraleda C, Benmessaoud R, Esteban J, López Y, Alami H, Barkat A, et al. Prevalence, antimicrobial resistance and serotype distribution of group B streptococcus isolated among pregnant women and newborns in Rabat, Morocco. J Med Microbiol. 2018;67(5):652-61. 10.1099/jmm.0.000720.

36. Arhoune B, Oumokhtar B, Hmami F, El Fakir S, Moutaouakkil K, Chami F, et al. Intestinal carriage of antibiotic resistant Acinetobacter baumannii among newborns hospitalized in Moroccan neonatal intensive care unit. PLoS One. 2019;14(1):e0209425. 10.1371/journal.pone.0209425.

37. Taoufik L, Amrani Hanchi A, Fatiha B, Nissrine S, Mrabih Rabou M, Nabila S. Emergence of OXA-48 Carbapenemase Producing Klebsiella pneumoniae in a Neonatal Intensive Care Unit in Marrakech, Morocco. Clin Med Insights Pediatr. 2019(13):1179556519834520. 10.1177/1179556519834524.

38. Touhami K, Sakkali H, Maaloum F, Diawara I, Touhami M, Bezzari M, et al. [Meningitis caused by Streptococcus pneumoniae serotype 7a in an infant vaccinated with two doses of 13-valent pneumococcal conjugate vaccine: A case study]. Méningite à Streptococcus pneumoniae sérotype 7A chez un nourrisson immunisé par deux doses du vaccin pneumococcique conjugué 13-valent: à propos d’un cas. Pan Afr Med J. 2019;32:203. 10.11604/pamj.2019.32.203.18157.

39. Elgarini M, Hammoumi A, Qasmaoui A, Hamamouchi J, Charof R, Mennane Z, et al. [First study of the seroprevalence of pertussis in pregnant women in Morocco]. Première étude de la séroprévalence de la coqueluche chez les femmes enceintes au Maroc. J de Pediatrie et de Pueric. 2020;33(3):146-50. 10.1016/j.jpp.2020.01.003.

40. Arhoune B, El Fakir S, Himri S, Moutaouakkil K, El Hassouni S, Benboubker M, et al. Intense intestinal carriage and subsequent acquisition of multidrug-resistant enterobacteria in neonatal intensive care unit in Morocco. PLoS One. 2021;16(6):e0251810. 10.1371/journal.pone.0251810.

41. Jbari S, Lahmini W, Boussaa S, Bourrous M. Impact of Covid-19 pandemic on pediatric meningitis incidence in central Morocco. Sci Afr. 2022;Jul(16):e01213. 10.1016/j.sciaf.2022.e01213.

42. Nachate S, Rouhi S, Ouassif H, Bennani H, Hachimi A, Mouaffak Y, et al. Multidrug-Resistant Bacteria Isolated from Blood Culture Samples in a Moroccan Tertiary Hospital: True Bacteremia or Contamination?. Infect Drug Resist. 2022;Sep 27(15):5691-704. 10.2147/IDR.S373065.

**Birth** (19)

43. Obermeyer C. Pluralism and pragmatism: knowledge and practice of birth in Morocco. Med Anthropol Q. 2000;14(2):180-201. 10.1525/maq.2000.14.2.180.

44. Obermeyer C. Risk, uncertainty, and agency: culture and safe motherhood in Morocco. Med Anthropol. 2000;19(2):173-201. 10.1080/01459740.2000.9966175.

45. Sefrioui O, Aboulfalah A, Taarji H, Matar N, el Mansouri A. [Current profile of obstetrical vesicovaginal fistulas at the maternity unit of the University of Casablanca]. Profil actuel des fistules vésicovaginales obstétricales à la maternité universitaire de Casablanca. Ann Urol (Paris). 2001;35(5):276-9. 10.1016/s0003-4401(01)00044-4.

46. Sefrioui O, Benabbes Taarji H, Azyez M, Aboulfalah A, el Karroumi M, Matar N, et al. [Vesico-uterine fistula of obstetrical origin. Report of 3 cases]. Les fistules vésico-utérines d'origine obstétricale. A propos de 3 cas. Ann Urol (Paris). 2002;36(6):376-80. 10.1016/s0003-4401(02)00129-8.

47. Hamada H, Zaki A, Nejjar H, Filali A, Chraibi C, Bezad R, et al. [Pregnancy and delivery in adolescents: characteristics and profile of 311 cases]. Grossesse et accouchement chez l'adolescente: caractéristiques et profil. À propos de 311 cas. J Gynecol Obstet Biol Reprod (Paris). 2004;33(7):607-14. 10.1016/s0368-2315(04)96601-x.

48. Paxton A, Bailey P, Lobis S, Fry D. Global patterns in availability of emergency obstetric care. Int J Gynaecol Obstet. 2006;93(3):300-7. 10.1016/j.ijgo.2006.01.030.

49. Leone T, Padmadas S, Matthews Z. Community factors affecting rising caesarean section rates in developing countries: an analysis of six countries. Soc Sci Med. 2008;67(8):1236-46. 10.1016/j.socscimed.2008.06.032.

50. Khawaja M, Choueiry N, Jurdi R. Hospital-based caesarean section in the Arab region: An overview. East Mediterr Health J. 2009;15(2):458-69. 10.26719/2009.15.2.458.

51. Capelli I. Risk and safety in context: medical pluralism and agency in childbirth in an eastern Moroccan oasis. Midwifery. 2011;27(6):781-5. 10.1016/j.midw.2010.06.020.

52. Kyu H, Shannon H, Georgiades K, Boyle M. Caesarean delivery and neonatal mortality rates in 46 low- and middle-income countries: A propensity-score matching and meta-analysis of demographic and health survey data. Int J Epidemiol. 2013;42(3):781-91. 10.1093/ije/dyt081.

53. Boutayeb W, Lamlili M, Boutayeb A. Health equity and access to health facility delivery and Caesarean sections in Morocco. Trop Med Int Health. 2015;20(11):1588-9. 10.1111/tmi.12571.

54. Cresswell J, Assarag B, Meski F, Filippi V, Ronsmans C. Trends in health facility deliveries and caesarean sections by wealth quintile in Morocco between 1987 and 2012. Trop Med Int Health. 2015;20(5):607-16. 10.1111/tmi.12466.

55. Elghanmi A, Seffar H. Puerperal hematoma: A cause of post partum hemorrhage after a normal vaginal delivery. Pan Afr Med J. 2015;20:365. 10.11604/pamj.2015.20.365.6478.

56. Sabiri N, Kabiri M, Razine R, Barkat A. Risk factors leading to preterm births in Morocco: A prospective study at the maternity Souissi in Rabat. Pan Afr Med J. 2015;22:21. 10.11604/pamj.2015.22.21.5100.

57. Benzouina S, Boubkraoui Mel M, Mrabet M, Chahid N, Kharbach A, El-Hassani A, et al. Fetal outcome in emergency versus elective cesarean sections at Souissi Maternity Hospital, Rabat, Morocco. Pan Afr Med J. 2016;23:197. 10.11604/pamj.2016.23.197.7401.

58. Benkirane S, Saadi H, Mimouni A. [Epidemiological profile of maternal complications related to cesarean section at the Al Farabi Hospital in Oujda]. Le profil épidémiologique des complications maternelles de la césarienne au CHR EL Farabi Oujda. Pan Afr Med J. 2017;27:108. 10.11604/pamj.2017.27.108.10036.

59. Xu B, Cheng H, Li X, Mouna N. Cesarean section rates and clinical indications at a large North African Hospital. J Reprod Med. 2017;62(5-6):291-4.

60. Hua Z, El Oualja F. Indicators for mode of delivery in pregnant women with uteruses scarred by prior caesarean section: a retrospective study of 679 pregnant women. BMC Pregnancy Childbirth. 2019;19(1):445. 10.1186/s12884-019-2604-0.

61. Zaidi H, Lamalmi N, Lahlou L, Slaoui M, Barkat A, Alamrani S, et al. Clinical predictive factors of histological chorioamnionitis: Case-control study. Heliyon. 2020;6(12):e05698. 10.1016/j.heliyon.2020.e05698.

**Breastfeeding** (17)

62. Hassani A, Barkat A, Souilmi F, Lyaghfouri A, Kabiri M, Karboubi L, et al. [Practices of breastfeeding. Study of 211 cases in the Maternity Hospital Souissi Rabat]. La conduite de l'allaitement maternel. Étude prospective de 211 cas à la maternité Souissi de Rabat. J de Pediatrie et de Pueric. 2005;18(7):343-8. 10.1016/j.jpp.2005.04.014.

63. Roida S, Hassia A, Maoulaininea F, Aboussada A. [Convenience of breastfeeding in the maternity of Marrakech]. Les pratiques de l’allaitement maternel à la maternité universitaire de Marrakech (Maroc). J de Pediatrie et de Pueric. 2010;23(2):70-5. 10.1016/j.jpp.2009.12.005.

64. Thimou Izgua A, Zouhair L, Mdaghri Alaoui A. [Breastfeeding premature infants after discharge from neonatal units: Prevalence and associated factors]. L’allaitement maternel chez les enfants prématurés après sortie de néonatologie : prévalence et facteurs associés. J de Pediatrie et de Pueric. 2012;25(2):73-80. 10.1016/j.jpp.2012.02.001.

65. Adarmouch L, Abourrahouat A, Sebbani M, Amine M, Sbihi M. [Weaning before the age of 6 months in Marrakech: associated factors and prevalence]. Sevrage des nourrissons avant six mois à Marrakech: facteurs associés et prévalence. Rev Epidemiol Sante Publique. 2013;61(5):429-35. 10.1016/j.respe.2013.05.023.

66. Bouhouch R, Bouhouch S, Cherkaoui M, Aboussad A, Stinca S, Haldimann M, et al. Direct iodine supplementation of infants versus supplementation of their breastfeeding mothers: A double-blind, randomised, placebo-controlled trial. Lancet Diabetes Endocrinol. 2014;2(3):197-209. 10.1016/S2213-8587%2813%2970155-4.

67. Berrani H, Mdaghri A, A, Kasouati J, Alaoui K, Thimou Izgua A. [Breastfeeding preterm infants at 6 months in Morocco: Prevalence and associated factors]. Allaitement maternel chez le nouveau-né prématuré à l'âge de six mois au Maroc : prévalence et facteurs associés. Arch Pediatr. 2015;22(2):141-5. 10.1016/j.arcped.2014.11.019.

68. Dold S, Zimmermann M, Aboussad A, Cherkaoui M, Jia Q, Jukic T, et al. Breast milk iodine concentration is a more accurate biomarker of iodine status than urinary iodine concentration in exclusively breastfeeding women. J Nutr. 2017;147(4):528-37. 10.3945/jn.116.242560.

69. Hamada H, Chala S, Barkat A, Lakhdar A. [Assessment of employment's impact on breastfeeding practices]. Évaluation de l’effet du travail sur la pratique de l’allaitement maternel. Arch Pediatr. 2017;24(8):720-7. 10.1016/j.arcped.2017.05.003.

70. Habibi M, Laamiri F, Aguenaou H, Doukkali L, Mrabet M, Barkat A. The impact of maternal socio-demographic characteristics on breastfeeding knowledge and practices: An experience from Casablanca, Morocco. Int J Pediatr Adolesc Med. 2018;5(2):39-48. 10.1016/j.ijpam.2018.01.003.

71. Jasny E, Amor H, Baali A. Mothers' knowledge and intentions of breastfeeding in Marrakech, Morocco. Arch Pediatr. 2019;26(5):285-9. 10.1016/j.arcped.2019.05.007.

72. Laamiri F, Barich F, Bennis A, Redouani M, Azzaoui S, Bentahila N, et al. [Knowledge and practice of women regarding breastfeeding maternal and impact of post-natal education on exclusive breastfeeding duration: A Morocco multicenter study]. Connaissances et pratiques des mères en matière d’allaitement maternel. J de Pediatrie et de Pueric. 2019;32(3):128-39. 10.1016/j.jpp.2019.02.006.

73. Ennasser H, Raoudi J, Taheri H, Saadi H, Mimouni A. [Idiopathic granulomatous mastitis: 4 case-reports and literature review]. La mastite granulomateuse idiopathique: à propos de 4 cas et revue de littérature. Pan Afr Med J. 2020;37:128. 10.11604/pamj.2020.37.128.25301.

74. Hamada H, Zaki A, Chala S. Female hospital workers in Morocco: factors influencing breastfeeding duration with self-reported experiences. Health Care Women Int. 2020;41(8):928-48. 10.1080/07399332.2020.1798964.

75. Mekaoui N, Belkouchi F, Chajai I, Dakhama B, Kerboubi L. How are newborns fed in their first day of life in Morocco? a survey. Pan Afr Med J. 2020;37:375. 10.11604/pamj.2020.37.375.25250.

76. Mulol H, Coutsoudis A, Amoussa Hounkpatin W, Urio E, Kenguela Wabolou P, Sissinto Y, et al. Is exclusive breastfeeding an option or a necessity in Africa? A pooled study using the deuterium oxide dose-to-mother technique. J Public Health Afr. 2020;11(1):932. 10.4081/jphia.2020.932.

77. El Moussaoui S, Kaoutar K, Chetoui A, El Kardoudi A, Chigr F, Borrous M, et al. Prevalence and determinant factors of exclusive breastfeeding practices among mothers in Marrakesh province, Morocco: A cross-sectional survey. Mediterr J Nutr Metab. 2021;14(3):265-75. 10.3233/MNM-200517.

78. Rabi B, Benjeddou K, Idrissi M, Rami A, Mekkaoui B, El Hamdouchi A, et al. Effects of breastfeeding on maternal body composition in Moroccan lactating women during twelve months after birth using stable isotopic dilution technique. Nutrients. 2021;13(1):146. 10.3390/nu13010146.

**Cancer** (6)

79. Soliman A, Allen K, Lo A, Banerjee M, Hablas A, Benider A, et al. Differences in reliability of reproductive history recall among women in North Africa. Int Electron J Health Educ. 2009;12(1):150-61.

80. Tissir R, Lamchahab M, Benhassou M, Quachouh M, Rachid M, Benchakroun S, et al. [Difficulty of the management of acute leukemia during pregnancy in Morocco]. Difficulté de la prise en charge de la leucémie aiguë au cours de la grossesse au Maroc. Pan Afr Med J. 2012;13:4. 10.11604/pamj.2012.13.4.1389.

81. Boufettal H, Khalkane L, Noun M, Hermas S, Samouh N. Gestational choriocarcinoma at Ibn Rochd Hospital, Casablanca, 2004-2010. East Mediterr Health J. 2014;19:S208-12.

82. Affdal A, Grynberg M, Hessissen L, Ravitsky V. Impact of legislation and public funding on oncofertility: a survey of Canadian, French and Moroccan pediatric hematologists/oncologists. BMC Med Ethics. 2020;21(1):25. 10.1186/s12910-020-00466-6.

83. Driouich Y, Haraj N, El Aziz S, Chadli A. Impact of pregnancy on papillary thyroid carcinoma prognosis. Pan Afr Med J. 2021;38:261. 10.11604/pamj.2021.38.261.22762.

84. Reynaud D, Abi Nahed R, Lemaitre N, Bolze P, Traboulsi W, Sergent F, et al. NLRP7 promotes choriocarcinoma growth and progression through the establishment of an immunosuppressive microenvironment. Cancers (Basel). 2021;13(12):2999. 10.3390/cancers13122999.

**Diabetes** (8)

85. Bouhsain S, Dami A, Elannaz H, Guelzim K, Baba H, Elhassani M, et al. [A critical study of the screening practices of gestational diabetes of a service of gynecology and obstetrics]. Etude critique des pratiques de dépistage du diabète gestationnel d'un service de gynécologie obstétrique. Ann Biol Clin (Paris). 2009;67(2):159-62. 10.1684/abc.2009.0309.

86. Bouhsain S, El Kochri S, Babahabib M, Hafidi M, Bouaiti E, Moussaoui M. [Comparing two screening policies of gestational diabetes mellitus: The Mohammed V Training Military Hospital of Rabat (Morocco)]. Comparaison de deux politiques de dépistage du diabète gestationnel: expérience de l'hôpital militaire d'instruction Mohammed V de Rabat (Maroc). Gynecol Obstet Fertil. 2014;42(5):317-21. 10.1016/j.gyobfe.2013.09.006.

87. Utz B, Assarag B, Essolbi A, Barkat A, Benkaddour Y, De Brouwere V. Diagnosis a posteriori? Assessing gestational diabetes screening and management in Morocco. Glob Health Action. 2016;9:32511. 10.3402/gha.v9.32511.

88. Utz B, Assarag B, Essolbi A, Barkat A, El Ansari N, Fakhir B, et al. Improving detection and initial management of gestational diabetes through the primary level of care in Morocco: Protocol for a cluster randomized controlled trial. Reprod Health. 2017;14(1):75. 10.1186/s12978-017-0336-z.

89. Utz B, Assarag B, Essolbi A, Barkat A, Delamou A, De Brouwere V. Knowledge and practice related to gestational diabetes among primary health care providers in Morocco: Potential for a defragmentation of care? Prim Care Diabetes. 2017;11(4):389-96. 10.1016/j.pcd.2017.04.005.

90. Utz B, Assarag B, Smekens T, Ennassiri H, Lekhal T, El Ansari N, et al. Detection and initial management of gestational diabetes through primary health care services in Morocco: An effectiveness-implementation trial. PLoS One. 2018;13(12):e0209322. 10.1371/journal.pone.0209322.

91. Chamlal H, Mziwira M, Ayachi M, Belahsen R. Prevalence of gestational diabetes and associated risk factors in the population of Safi Province in Morocco. Pan Afr Med J. 2020;37:281. 10.11604/pamj.2020.37.281.21798.

92. Utz B, Assarag B, Lekhal T, Van Damme W, De Brouwere V. Implementation of a new program of gestational diabetes screening and management in Morocco: A qualitative exploration of health workers' perceptions. BMC Pregnancy Childbirth. 2020;20(1):315. 10.1186/s12884-020-02979-9.

**Environment** (9)

93. Souad C, Farida Z, Nadra L, François B, Bougle D, Azeddine S. Trace element level in infant hair and diet, and in the local environment of the Moroccan city of Marrakech. Sci Total Environ. 2006;370(2-3):337-42. 10.1016/j.scitotenv.2006.06.020.

94. Zaida F, Chadrame S, Sedki A, Lekouch N, Bureau F, Arhan P, et al. Lead and aluminium levels in infants' hair, diet, and the local environment in the Moroccan city of Marrakech. Sci Total Environ. 2007;377(2-3):152-8. 10.1016/j.scitotenv.2006.10.017.

95. El Koraichi A, Ghannam A, Talha M, Chmitah O, Al Haddoury M, El Kettani S. [Acute percutaneous organophosphate poisoning: about a pediatric case]. Intoxication aiguë transcutanée par organophosphorés: à propos d'une observation pédiatrique. Presse Med. 2011;40(11):1083-5. 10.1016/j.lpm.2011.03.008.

96. Cherkani-Hassani A, Ghanname I, Zinedine A, Sefrioui H, Qmichou Z, Mouane N. Aflatoxin M1 prevalence in breast milk in Morocco: Associated factors and health risk assessment of newborns CONTAMILK study. Toxicon. 2020;187:203-8. 10.1016/j.toxicon.2020.09.008.

97. Cherkani-Hassani A, Slaoui M, Ghanname I, Mojemmi B, Belhaj A, Kers B, et al. Cadmium contamination in breast milk of Moroccan lactating women and the associated factors: CONTAMILK Study. Biol Trace Elem Res. 2020;196(1):47-59. 10.1007/s12011-019-01912-2.

98. Cherkani-Hassani A, Slaoui M, Ghanname I, Mojemmi B, Belhaj A, Kers B, et al. Levels of mercury in Moroccan breast milk and the affecting factors: CONTAMILK study. Regul Toxicol Pharmacol. 2021;127:105065. 10.1016/j.yrtph.2021.105065.

99. Cherkani-Hassani A, Slaoui M, Ghanname I, Mojemmi B, Eljaoudi R, Belhaj A, et al. Lead concentrations in breast milk of Moroccan nursing mothers and associated factors of exposure: CONTAMILK STUDY. Environ Toxicol Pharmacol. 2021;85:103629. 10.1016/j.etap.2021.103629.

100. Jeddi Z, Gryech I, Ghogho M, El Hammoumi M, Mahraoui C. Machine learning for predicting the risk for childhood asthma using prenatal, perinatal, postnatal and environmental factors. Healthcare (Basel). 2021;9(11):1464. 10.3390/healthcare9111464.

101. Cherkani-Hassani A, Ghanname I, Zinedine A, Sefrioui H, Qmichou Z, Mouane N. Ochratoxin a in breast milk in Morocco: the affecting dietary habits of the lactating mothers and the degree of exposure of newborns CONTAMILK study. Drug Chem Toxicol. 2022;45(3):1081-7. 10.1080/01480545.2020.1808669.

**Family Planning** (13)

102. Crognier E, Baali A, Hilali M. Do helpers at the nest increase their parents' reproductive success? Am J Hum Biol. 2001;13(3):365-73. 10.1002/ajhb.1060.

103. Eltigani E. Childbearing in five Arab countries. Stud Fam Plann. 2001;32(1):17-24. 10.1111/j.1728-4465.2001.00017.x.

104. Crognier E. Reproductive success: Which meaning? Am J Hum Biol. 2003;15(3):352-60. 10.1002/ajhb.10153.

105. Agha S, Do M, Armand F. When donor support ends: The fate of social marketing products and the markets they help create. Social Marketing Quarterly. 2006;12(2):28-42. 10.1080/15245000600678489.

106. Crognier E, Baali A, Hilali M, Villena M, Vargas E. Preference for sons and sex ratio in two non-western societies. Am J Hum Biol. 2006;18(3):325-34. 10.1002/ajhb.20499.

107. Sullivan T, Bertrand J, Rice J, Shelton J. Skewed contraceptive method mix: why it happens, why it matters. J Biosoc Sci. 2006;38(4):501-21. 10.1017/S0021932005026647.

108. Agha S, Do M. Does an expansion in private sector contraceptive supply increase inequality in modern contraceptive use? Health Policy Plan. 2008;23(6):465-75. 10.1093/heapol/czn035.

109. Ali M, Cleland J. Oral contraceptive discontinuation and its aftermath in 19 developing countries. Contraception. 2010;81(1):22-9. 10.1016/j.contraception.2009.06.009.

110. Hughes CL. The amazing fertility decline: Islam, economics, and reproductive decision making among working-class Moroccan women. Med Anthropol Q. 2011;25(4):417-35. 10.1111/j.1548-1387.2011.01178.x.

111. Ali M, Park M, Ngo T. Levels and determinants of switching following intrauterine device discontinuation in 14 developing countries. Contraception. 2014;90(1):47-53. 10.1016/j.contraception.2014.03.008.

112. Rinker CH. Creating neoliberal citizens in Morocco: Reproductive health, development policy, and popular Islamic beliefs. Med Anthropol. 2015;34(3):226-42. 10.1080/01459740.2014.922082.

113. Benbella A, Aboulmakarim S, Hardizi H, Zaidouni A, Bezad R. Infertility in the Moroccan population: an etiological study in the reproductive health centre in Rabat. Pan Afr Med J. 2018;30:204. 10.11604/pamj.2018.30.204.13498.

114. Benbella A, Aboulmakarim S, Hardizi H, Zaidouni A, Bezad R. Infertility in the Moroccan population: major risk factors encountered in the reproductive health centre in Rabat. Pan Afr Med J. 2018;30:195. 10.11604/pamj.2018.30.195.13849.

**Genetics** (23)

115. Jaouad I, Ouldim K, Ali Ou Alla S, Kriouile Y, Villa A, Sefiani A. Omenn syndrome with mutation in RAG1 gene. Indian J Pediatr. 2008;75(9):944-6. 10.1007/s12098-008-0197-0.

116. Zakaria A, Kabiri M, Kabbaj M, Barkat A, Lamdouar Bouazzaoui N. [Diffuse arterial calcified elastopathy. A case report]. Élastopathie calcifiante artérielle diffuse. À propos d’un cas. Arch Pediatr. 2009;16(11):1474-6. 10.1016/j.arcped.2009.08.006.

117. Ratbi I, Elalaoui S, Sefiani A. Two infants with Beckwith-Wiedemann Syndrome. Balkan Journal of Medical Genetics. 2010;13(1):49-53. 10.2478/v10034-010-0019-3.

118. Achargui S, Tijane M, Benchemsi N. [Fetal RHD genotyping by PCR using plasma from D negative pregnant women]. Génotypage RHD fœtal par PCR dans le plasma de femmes enceintes D négatif. Transfus Clin Biol. 2011;18(1):13-9. 10.1016/j.tracli.2010.10.002.

119. Sbiti A, Ratbi I, Kriouile Y, Sefiani A. [Spinal muscular atrophy: A frequent cause of congenital hypotonia in Morocco]. L’amyotrophie spinale infantile : cause fréquente des hypotonies congénitales au Maroc. Arch Pediatr. 2011;18(12):1261-4. 10.1016/j.arcped.2011.09.025.

120. Doubaj Y, Laarabi F, Elalaoui S, Barkat A, Sefiani A. Carrier frequency of the recurrent mutation c.1643_1644delTG in the XPC gene and birth prevalence of the xeroderma pigmentosum in Morocco. J Dermatol. 2012;39(4):382-4. 10.1111/j.1346-8138.2011.01453.x.

121. Lamzouri A, Ratbi I, Laarabi F, Barkat A, Sefiani A. Low prevalence of p.g352fsdelg mutation in phenylketonuria patients from Morocco. Genet Test Mol Biomarkers. 2012;16(8):996-8. 10.1089/gtmb.2012.0011.

122. Lamzouri A, Natiq A, Tajir M, Sendid M, Sefiani A. [Prenatal diagnosis of trisomy 21 by fluorescence in situ hybridization (FISH): about the first tests in Morocco]. Le diagnostic anténatal de la trisomie 21 par l'hybridation in situ en fluorescence (FISH): À propos des premiers tests réalisés au Maroc. Pan Afr Med J. 2012;13:38.

123. Lyahyai J, Sbiti A, Barkat A, Ratbi I, Sefiani A. Spinal muscular atrophy carrier frequency and estimated prevalence of the disease in Moroccan newborns. Genet Test Mol Biomarkers. 2012;16(3):215-8. 10.1089/gtmb.2011.0149.

124. Tajir M, Elmachad M, Kabbaj N, Laarabi F, Barkat A, Amrani N, et al. Frequency of IL28B rs12979860 single-nucleotide polymorphism alleles in newborn infants and in patients with chronic hepatitis C in Morocco. Genet Test Mol Biomarkers. 2012;16(8):981-3. 10.1089/gtmb.2011.0353.

125. Doubaj Y, Pingault V, Elalaoui S, Ratbi I, Azouz M, Zerhouni H, et al. A novel mutation in the endothelin B receptor gene in a Moroccan family with Shah-Waardenburg syndrome. Mol Syndromol. 2015;6(1):44-9. 10.1159/000371590.

126. Es-Seddiki A, Rkain M, Ayyad A, Nkhili H, Amrani R, Benajiba N. [Partial facial duplication (a rare diprosopus): Case report and review of the literature]. Duplication faciale partielle (un diprosope rare): à propos d'un cas et revue de littérature. Rev Stomatol Chir Maxillofac Chir Orale. 2015;116(6):376-9. 10.1016/j.revsto.2015.09.004.

127. Zerkaoui M, Ratbi I, Castellotti B, Lyahyai J, Sefiani A, Castellotti B, et al. Clinical and molecular report of novel GALC mutations in Moroccan patient with Krabbe disease: Case report. BMC Pediatr. 2015;15(1):182. 10.1186/s12887-015-0490-9.

128. Jouali F, Marchoudi N, Rouissi A, Fekkak J. Identification of a novel nonsense variant in the SCN1A gene that causes Febrile Seizure Disorder. Pediatr Neurol. 2018;16(4):236-8. 10.1055/s-0037-1607995.

129. Elkarhat Z, Kindil Z, Zarouf L, Razoki L, Aboulfaraj J, Elbakay C, et al. Chromosomal abnormalities in couples with recurrent spontaneous miscarriage: a 21-year retrospective study, a report of a novel insertion, and a literature review. J Assist Reprod Genet. 2019;36(3):499-507. 10.1007/s10815-018-1373-4.

130. Laghmich A, Alaoui Ismaili F, Barakat A, Ghailani Nourouti N, Khattab M, Bennani Mechita M. Alpha-Thalassemia in North Morocco: Prevalence and molecular spectrum. Biomed Res Int. 2019;13(2019):2080352. 10.1155/2019/2080352.

131. Zahir H, Chakour M, Mouhib H, Yahyaoui H, Ait Ameur M. [Epidemiological, clinico-biological, therapeutic and evolutionary aspects of beta-thalassemia in Morocco]. Aspect épidémiologique, clinico-biologique, thérapeutique et évolutif de la ?-thalassémie au Maroc. Ann Biol Clin (Paris). 2019;77(2):169-73. 10.1684/abc.2019.1433.

132. El Moussaoui S, Bennaoui F, El Idrissi Slitine N, Houcar O, Maoulainine F. Biotinidase deficiency in a newborn. J Neonatal Perinatal Med. 2020;13(1):139-41. 10.3233/NPM-180130.

133. Lehlimi M, El Korchi Z, Chemsi M, Badre A, Habzi A, Benomar S. [Maternal-fetal incompatibility in the ABO system]. L’incompatibilité fœto-maternelle dans le système ABO. J de Pediatrie et de Pueric. 2020;33(3):151-7. 10.1016/j.jpp.2019.11.002.

134. Outtaleb F, Errahli R, Imelloul N, Jabrane G, Serbati N, Dehbi H. [Trisomy 18 or postnatal Edward's syndrome: descriptive study conducted at the University Hospital Center of Casablanca and literature review]. La trisomie 18 ou syndrome d'Edwards en post-natal: étude descriptive au Centre Hospitalier Universitaire de Casablanca et revue de littérature. Pan Afr Med J. 2020;37:309. 10.11604/pamj.2020.37.309.26205.

135. Benhsaien I, Essadssi S, Elkhattabi L, Bakhchane A, Abdelghaffar H, Bousfiha A, et al. Omenn syndrome caused by a novel homozygous mutation in recombination activating gene 1. Immunobiology. 2021;226(3):152090. 10.1016/j.imbio.2021.152090.

136. Meiouet F, Kabbaj S, Debray F, Boemer F. [Diagnosis and monitoring of phenylketonuria by LC-MS-MS in Morocco]. Diagnostic et suivi de la phénylcétonurie par LC-MS-MS au Maroc. Ann Biol Clin (Paris). 2021;79(1):49-55. 10.1684/abc.2021.1619.

137. van Wegberg A, Trefz F, Gizewska M, Ahmed S, Chabraoui L, Zaki M, et al. Undiagnosed phenylketonuria can exist everywhere: Results from an international survey. J Pediatr. 2021;239:231-. 10.1016/j.jpeds.2021.08.070.

**Gynecology** (3)

138. Zouhair K, El Ouazzani T, El Omari K, El Fajri S, Lakhdar H. [Vulvar pathology]. La pathologie vulvaire. East Mediterr Health J. 2002;8(6):812-8.

139. Kadi N, Tahiri L, Maziane M, Mernissi F, Harzy T. Proximal symphalangism and premature ovarian failure. Joint Bone Spine. 2012;79(1):83-4. 10.1016/j.jbspin.2011.05.029.

140. Lamrissi A, Mabengui A, Mourabbih M, Jalal M, Fichtali K, Bouhya S. Acquired uterine arterio-venous malformation post molar pregnancy suction-curettage: 2 case reports. Int J Surg Case Rep. 2022;94:107105. 10.1016/j.ijscr.2022.107105.

**Infant Morbidity** (20)

141. Bourrous M, Elmjati H, Amine M, El Omari J, Bouskraoui M. [Diarrheal disease in the region of Marrakech, Morocco]. Enquête sur la prise en charge de la maladie diarrhéique dans la région de Marrakech (Maroc). Med Trop. 2010;70(2):145-8.

142. Arsalane L, Zouhair S, Lahlou Amine I, Louzi L, Bouskraoui M. [Urinary tract infection in infants (376 cases) in a Moroccan hospital (2009-2010) - etiologic frequency and prevalence of resistance]. Infection urinaire du nourrisson (376 cas) dans un hôpital marocain (2009-2010)--fréquence étiologique et prévalence de la résistance. Pathol Biol. 2012;60(6):e90-e1. 10.1016/j.patbio.2012.01.004.

143. Dibi A, Jabourik F, Bentahila A. [Psoriasis in infants about five cases]. Le psoriasis chez le nourrisson à propos de cinq cas. Arch Pediatr. 2012;19(2):220-3. 10.1016/j.arcped.2011.12.003.

144. Rami M, Mahmoudi A, ElMadi A, Khalid, Khattala, Afifi A, et al. [Cervical teratoma: report of 2 cases]. Le tératome cervical: à propos de 2 cas. Pan Afr Med J. 2012;12:91.

145. Elmachtani Idrissi S, El Omri N, El Jaoudi R, Chibani F, Biaze A, Dami A, et al. [Maternal vitamin B12 deficiency: What consequences for the newborn? About Moroccan case]. La carence maternelle en vitamine B12 : quelles conséquences pour le nouveau-né ? À propos d’un cas marocain. Immuno-anal Biol Spe. 2013;28(5-6):362-5. 10.1016/j.immbio.2013.06.002.

146. Sabiri N, Kabiri M, Kharbach A, Berrada R, Barkat A, Razine R. Congenital malformations risk factors: Prospective study of Rabat Souissi maternity in Morocco. Facteurs de risque des malformations congénitales : étude prospective à la maternité Souissi de Rabat au Maroc. J de Pediatrie et de Pueric. 2013;26(4):198-203. 10.1016/j.jpp.2013.05.001.

147. Baba L, Ailal F, El Hafidi N, Hubeau M, Jabot-Hanin F, Benajiba N, et al. Chronic granulomatous disease in Morocco: genetic, immunological, and clinical features of 12 patients from 10 kindreds. J Clin Immunol. 2014;34(4):452-8. 10.1007/s10875-014-9997-3.

148. Maleb A, Nya F, Amahzoune B, Lemnouer A, Elouennass M. Postoperative mediastinitis due to Candida tropicalis: First reported case in Morocco. Médiastinite post-chirurgicale à Candida tropicalis : premier cas rapporté au Maroc. J Mycol Med. 2014;24(3):225-8. 10.1016/j.mycmed.2014.03.006.

149. Radouani M, Chahid N, Benmiloud L, Elammari L, Lahlou K, Barkat A. [Epidemiology and risk factors of the closing neural tube defects: Moroccan data]. Épidémiologie et facteurs de risque des anomalies de fermeture du tube neural: données marocaines. Pan Afr Med J. 2015;22:43. 10.11604/pamj.2015.22.43.5158.

150. El Fakiri K, Bourouhouat A, Ait Sab I, Sbihi M. [Cholestasis neonatal and infant: Marrakech University Hospital experience]. Les cholestases du nouveau-né et du nourrisson : expérience du CHU de Marrakech. J de Pediatrie et de Pueric. 2016;29(3):139-43. 10.1016/j.jpp.2016.02.004.

151. El Amraoui W, Bentalha A, Hamri H, Es-Chrif El Kettani S, El Koraichi A. Congenital cystic adenomatoid malformation - dangers of misdiagnosis: a case report. J Med Case Rep. 2017;11(1):212. 10.1186/s13256-017-1349-5.

152. Es Seddiki A, Messaouidi S, Amrani R. [The role of fenugreek in the occurrence of an anomaly of neural tube closure: Warning signal from Morocco]. Le rôle du fenugrec dans la survenue d’anomalie de fermeture du tube neural : un signal d’alerte depuis le Maroc. Phytothérapie. 2017;15(3):155-8. 10.1007/s10298-017-1128-x.

153. Indrio F, Miqdady M, Al Aql F, Haddad J, Karima B, Khatami K, et al. Knowledge, attitudes, and practices of pediatricians on infantile colic in the Middle East and North Africa region. BMC Pediatr. 2017;17(1):187. 10.1186/s12887-017-0939-0.

154. Ouladsaiad M, Aballa N, Kamili E, Fouraiji K. Unusual Ileal Anomalies Associated with Omphalomesenteric Duct Remnants. Afr J Paediatr Surg. 2017;14(2):32-3. 10.4103/ajps.AJPS_67_16.

155. Vandenplas Y, AlFrayh A, Al Mutairi B, Elhalik M, Green R, Haddad J, et al. Physician practice in food allergy prevention in the Middle East and North Africa. BMC Pediatr. 2017;17:118. 10.1186/s12887-017-0871-3.

156. Lahrichi A, Hali F, Baline K, Fatoiki F, Chiheb S, Khadir K. Effects of propranolol therapy in Moroccan children with infantile hemangioma. Arch Pediatr. 2018;25(8):449-51. 10.1016/j.arcped.2018.09.002.

157. Elghanmi A, Razine R, Jou M, Berrada R. Congenital malformations among newborns in Morocco: A retrospective study. Pediatr Rep. 2020;12(1):7405. 10.4081/pr.2020.7405.

158. Forci K, Alami M, Bouaiti E, Slaoui M, Mdaghri Alaoui A, Thimou Izgua A. Prevalence of congenital malformations at the les Orangers maternity and reproductive health Hospital of Rabat: descriptive study of 470 anomalies. BMC Pediatr. 2020;20(1):272. 10.1186/s12887-020-02179-6.

159. Forci K, Bouaiti E, Alami M, Mdaghri Alaoui A, Thimou Izgua A. Incidence of neural tube defects and their risk factors within a cohort of Moroccan newborn infants. BMC Pediatr. 2021;21(1):124. 10.1186/s12887-021-02584-5.

160. Saiad M. A wave-like anastomosis, a new technique of anastomosis to prevent stricture after oesophageal atresia repair. Afr J Paediatr Surg. 2021;18(4):179-81. 10.4103/ajps.AJPS_123_20.

**Infant Mortality** (4)

161. El Masnaoui N, Barkat A, Hatou F, Kabiri M, Lamdouar Bouazzaoui N. [Perinatal mortality outcome in high-order multiple pregnancy]. Facteurs impliqués dans la mortalité périnatale des nouveau-nés issus de grossesses multiples de haut rang. J de Pediatrie et de Pueric. 2009;22(4-5):193-6. 10.1016/j.jpp.2009.05.005.

162. Sabiri N, Kabiri M, Karboubi L, Bouziane A, Barkat A. Risk factors for perinatal mortality at Souissi Maternity Hospital, Rabat, Morocco. Int J Gynaecol Obstet. 2012;119(3):285-6. 10.1016/j.ijgo.2012.07.004.

163. Ouahid H, Adarmouch L, Belouali R, Mouwafaq S, Soummani A. [Factors associated with intrapartum and very early neonatal mortality at the materniry of University hospital Mohamed VI, Marrakech, Morocco. Case-control study]. Les facteurs associés à la mortalité intrapartum et néonatale très précoce au niveau de la maternité du centre hospitalier universitaire Mohammed VI, Marrakech, Maroc. Étude cas témoin. Rev Epidemiol Sante Publique. 2019;67(4):233-8. 10.1016/j.respe.2019.03.122.

164. Nya S, Abouzahir H, Belhouss A, Benyaich H. Unexpected death of an infant suffocated in the course of breastfeeding when the mother fell asleep. Med Leg J. 2021;89(2):139-42. 10.1177/0025817220980677.

**Infant Near-Miss** (1)

165. Ronsmans C, Cresswell J, Goufodji S, Agbla S, Ganaba R, Assarag B, et al. Characteristics of neonatal near miss in hospitals in Benin, Burkina Faso and Morocco in 2012-2013. Trop Med Int Health. 2016;21(4):535-45. 10.1111/tmi.12682.

**Legal** (1)

166. Newman J. There Is a Big Question Mark: Managing ambiguity in a Moroccan maternity ward. Med Anthropol Q. 2019;33(3):386-402. 10.1111/maq.12510.

**Maternal Morbidity** (24)

167. Hachim K, Badahi K, Benghanem M, Fatihi E, Zahiri K, Ramdani B, et al. [Obstetrical acute renal failure. Experience of the nephrology department, Central University Hospital ibn Rochd, Casablanca]. Insuffisance rénale aiguë obstétricale. L'expérience du Service de néphrologie, Chu ibn Rochd, Casablanca. Nephrologie. 2001;22(1):29-31.

168. Fellat I, Oukerraj L, Doghmi N, Bennani R, Fellat N, Elhaitem N, et al. [Percutaneous mitral valvuloplasty in the pregnant woman Moroccan experience]. Valvuloplastie mitrale percutanée chez la femme enceinte: expérience marocaine. Ann Cardiol Angeiol (Paris). 2003;52(3):139-42. 10.1016/s0003-3928(03)00060-x.

169. Agoub M, Moussaoui D, Battas O. Prevalence of postpartum depression in a Moroccan sample. Arch Womens Ment Health. 2005;8(1):37-43. 10.1007/s00737-005-0069-9.

170. Alami K, Kadri N, Berrada S. Prevalence and psychosocial correlates of depressed mood during pregnancy and after childbirth in a Moroccan sample. Arch Womens Ment Health. 2006;9(6):343-6. 10.1007/s00737-006-0154-8.

171. El Bouazzaoui A, Labib S, Derkaoui A, Adnane Berdai M, Bendadi A, Harandou M. Dislocation of temporo-mandibular joint - An uncommon circumstance of occurrence: Vaginal delivery. Pan Afr Med J. 2010;5:23.

172. Bentata Y, Housni B, Mimouni A, Abouqal R. Obstetric acute renal failure in an intensive care unit in Morocco. Int J Gynaecol Obstet. 2011;115(2):196-8. 10.1016/j.ijgo.2011.05.029.

173. Miguil M, Salmi S, Moussaid I, Benyounes R. [Acute renal failure requiring haemodialysis in obstetrics]. Insuffisance rénale aiguë hémodialysée en obstétrique. Nephrol Ther. 2011;7(3):178-81. 10.1016/j.nephro.2010.11.005.

174. Bentata Y, Housni B, Mimouni A, Abouqal R. Admissions of women in the third trimester of pregnancy to an intensive care unit in Morocco over a 4-year period. Int J Gynaecol Obstet. 2012;116(3):260-1. 10.1016/j.ijgo.2011.10.021.

175. Bentata Y, Housni B, Mimouni A, Azzouzi A, Abouqal R. Acute kidney injury related to pregnancy in developing countries: etiology and risk factors in an intensive care unit. J Nephrol. 2012;25(5):764-75. 10.5301/jn.5000058.

176. Arrayhani M, El Youbi R, Sqalli T. Pregnancy-related acute kidney injury: experience of the nephrology unit at the university hospital of Fez, Morocco. ISRN Nephrol. 2013;2013:109034. 10.5402/2013/109034.

177. Assarag B, Dubourg D, Maaroufi A, Dujardin B, De Brouwere V. Maternal postpartum morbidity in Marrakech: What women feel what doctors diagnose? BMC Pregnancy Childbirth. 2013;13:225. 10.1186/1471-2393-13-225.

178. Sasbou T, Tachinante R, Tazi Saoud A, Ksayr R, Ben Rais Aouad N. [Place of scintigraphy in the diagnosis of pulmonary embolism in pregnant women-About 17 cases]. Place de la scintigraphie dans le diagnostic de l’embolie pulmonaire chez la femme enceinte – À propos de 17 cas. Med Nucleaire. 2013;37(10-11):439-45. 10.1016/j.mednuc.2013.09.006.

179. Assarag B, Dujardin B, Essolbi A, Cherkaoui I, De Brouwere V. Consequences of severe obstetric complications on women's health in Morocco: please, listen to me! Trop Med Int Health. 2015;20(11):1406-14. 10.1111/tmi.12586.

180. Elghanmi A, Mohamed J, Khabouz S. Spontaneous splenic rupture in pregnancy. Pan Afr Med J. 2015;21:312. 10.11604/pamj.2015.21.312.6878.

181. Elkhoudri N, Amor H, Baali A. Self-reported postpartum morbidity: Prevalence and determinants among women in Marrakesh, Morocco. Reprod Health. 2015;12(1):75. 10.1186/s12978-015-0066-z.

182. Kabbali N, Tachfouti N, Arrayhani M, Harandou M, Tagnaouti M, Bentata Y, et al. Outcome assessment of pregnancy-related acute kidney injury in Morocco: A national prospective study. Saudi J Kidney Dis Transpl. 2015;26(3):619-24. 10.4103/1319-2442.157426.

183. Benkirane S, Saadi H, Serji B, Mimouni A. Uterine necrosis following a combination of uterine compression sutures and vascular ligation during a postpartum hemorrhage: A case report. Int J Surg Case Rep. 2017;38:5-7. 10.1016/j.ijscr.2017.07.005.

184. Ait Addi R, Benksim A, Zouini M, Cherkaoui M. A cross-sectional study of socio-demographic characteristics of pregnant women on the dental and periodontal health. Asian J Epidemiol. 2018;11(1):14-9. 10.3923/aje.2018.14.19.

185. Ait Addi R, Benksim A, Bahije L, Cherkaoui M. Sociodemographic disparities and self-reported oral health problems associated with pregnancy: A case-control study in Morocco. Electron J Gen Med. 2020;17(5):em249. 10.29333/ejgm/8238.

186. Moussaoui K, Zraidi N, Baidada A, Kharbach A. [Uterine necrosis following uterine compressions in combination with vascular ligation during postpartum hemorrhage: A case report]. Nécrose utérine suite à une combinaison de compressions utérines et de ligature vasculaire lors d'une hémorragie post-partum: à propos d’un cas. Pan Afr Med J. 2020;37:279. 10.11604/pamj.2020.37.279.26788.

187. Bouhaddoune Y, Hbali A, Aissaoui H, Mrabet A, Ismaili N, El Ouafi N. Peripartum cardiomyopathy: alluring challenge - case series and review of literature. Pan Afr Med J. 2021;40:119. 10.11604/pamj.2021.40.119.29168.

188. Jabi R, Elmir S, Saoud K, Ali H, Nasri S, Skiker I, et al. Strangled gravidic uterus, an exceptional complication of umbilical hernia during pregnancy, a case report. Ann Med Surg (Lond). 2021;72:103143. 10.1016/j.amsu.2021.103143.

189. Hassine A, Ould dadda M, Yassyn S, Bezad R, Alami M, Filali A, et al. [Pregnancy in a triple scarred septate uterus brought to term: About a case and review of the literature]. Grossesse sur utérus cloisonné triplement cicatriciel menée à terme: à propos d'un cas et revue de la litérature. International Journal of Advances in Surgery. 2022;6(4):1-2. 10.34300/26630451.

190. Manoussi A, Baali A, Amor H, Ouzennou N. [Maternal morbidity: situation and determinants between 2020 and 2021 in the province of Essaouira, Morocco]. La morbidité maternelle: situation et déterminants entre 2020 et 2021 dans la province d'Essaouira, Maroc. Sante Publique. 2022;34(4):581-90. 10.3917/spub.224.0581.

**Maternal Mortality** (9)

191. Abouchadi S, Alaoui A, Meski F, Bezad R, De Brouwere V. Preventable maternal mortality in Morocco: the role of hospitals. Trop Med Int Health. 2013;18(4):444-50. 10.1111/tmi.12065.

192. Abouchadi S, Belghiti Alaoui A, Meski F, De Brouwere V. Implementation and outcomes of a national maternal mortality monitoring system in Morocco 2008-2009. Trop Med Int Health. 2013;18(3):357-65. 10.1111/tmi.12053.

193. Abouchadi S, Belghiti Alaoui A, Meski F, De Brouwere V. Implementing a maternal mortality surveillance system in Morocco - challenges and opportunities. Trop Med Int Health. 2013;18(3):357-65. 10.1111/tmi.12053.

194. Collaborators GMM. Global, regional, and national levels of maternal mortality, 1990-2015: a systematic analysis for the Global Burden of Disease Study 2015. Obstet Synecol Surv. 2016;72(1):11-3. 10.1097/01.ogx.0000511935.64476.66.

195. Abouchadi S, Zhang W, De Brouwere V. Underreporting of deaths in the maternal deaths surveillance system in one region of Morocco. PLoS One. 2018;13(1):e0188070. 10.1371/journal.pone.0188070.

196. Nieto-Calvache A, Palacios-Jaraquemada J, Osanan G, Cortes-Charry R, Aryananda R, Bangal V, et al. Lack of experience is a main cause of maternal death in placenta accreta spectrum patients. Acta Obstet Gynecol Scand. 2021;100(8):1445-53. 10.1111/aogs.14163.

197. Abouchadi S, Godin I, Zhang W, De Brouwere V. Eight-year experience of maternal death surveillance in Morocco: qualitative study of stakeholders' views at a subnational level. BMC Public Health. 2022;22(1):2111. 10.1186/s12889-022-14556-0.

198. Nichols E, Pettrone K, Vickers B, Gebrehiwet H, Surek-Clark C, Leitao J, et al. Mixed-methods analysis of select issues reported in the 2016 World Health Organization verbal autopsy questionnaire. PLoS One. 2022;17(10):e0274304. 10.1371/journal.pone.0274304.

199. Okafor O, Roos N, Abdosh A, Adesina O, Alaoui Z, Romero W, et al. International virtual confidential reviews of infection-related maternal deaths and near-miss in 11 low- and middle-income countries --case report series and suggested actions. BMC Pregnancy Childbirth. 2022;22(1):44575. 10.1186/s12884-022-04731-x.

**Maternal Near-Miss** (5)

200. Sahel A, Brouwere V, Lardi M, Lerberghe W, Ronsmans C, Filippi V. [Obstetric catastrophes barely just avoided: Near misses in Moroccan hospitals]. Des catastrophes obstétricales évitées de justesse: les near miss dans les hôpitaux marocains. Sante. 2001;11(4):229-35.

201. Richard F, Filali H, Lardi M, de Brouwere V. [Hospital deliveries in Morocco or how to reconcile different logics]. Accouchement à l'hôpital au Maroc ou comment concilier des logiques différentes. Rev Epidemiol Sante Publique. 2003;51(1 Pt 1):39-54.

202. Filippi V, Brugha R, Browne E, Gohou V, Bacci A, De Brouwere V, et al. Obstetric audit in resource-poor settings: Lessons from a multi-country project auditing 'near miss' obstetrical emergencies. Health Policy Plan. 2004;19(1):57-66. 10.1093/heapol/czh007.

203. Filippi V, Ronsmans C, Gohou V, Goufodji S, Lardi M, Sahel A, et al. Maternity wards or emergency obstetric rooms? Incidence of near-miss events in African hospitals. Acta Obstet Gynecol Scand. 2005;84(1):11-6. 10.1111/j.0001-6349.2005.00636.x.

204. Assarag B, Dujardin B, Delamou A, Meski F, De Brouwere V. Determinants of maternal near-miss in Morocco: Too late, too far, too sloppy? PLoS One. 2015;10(1):e0116675. 10.1371/journal.pone.0116675.

**Midwifery** (10)

205. Temmar F, Vissandjee B, Kerouac S. Strengthening midwifery practices in Morocco: a gender perspective. International Midwifery. 2005;18(1).

206. Temmar F, Vissandjée B, Hatem M, Apale A, Kobluk D. Midwives in Morocco: Seeking recognition as skilled partners in women-centred maternity care. Reprod Health Matters. 2006;14(27):83-90. 10.1016/S0968-8080(06)27245-8.

207. Van Lerberghe W, Matthews Z, Achadi E, Ancona C, Campbell J, Channon A, et al. Country experience with strengthening of health systems and deployment of midwives in countries with high maternal mortality. Lancet. 2014;384(9949):1215-25. 10.1016/S0140-6736%2814%2960919-3.

208. Abou Malham S, Hatem M, Leduc N. A case study evaluation of an intervention aiming to strengthen the midwifery professional role in Morocco: Anticipated barriers to reaching outcomes. J Multidiscip Healthc. 2015;8:419-32. 10.2147/JMDH.S86920.

209. Abou-Malham S, Hatem M, Leduc N. Analyzing barriers and facilitators to the implementation of an action plan to strengthen the midwifery professional role: a Moroccan case study. BMC Health Serv Res. 2015;15:382. 10.1186/s12913-015-1037-3.

210. Martínez-Linares J, Linares-Abad M, Calero-García M, López-Entrambasaguas O. Is it possible to become a midwife in a refugee camp? Midwifery. 2019;75:12-5. 10.1016/j.midw.2019.04.002.

211. Cadée F, Nieuwenhuijze M, Lagro-Janssen A, de Vries R. Paving the way for successful twinning: Using grounded theory to understand the contribution of twin pairs in twinning collaborations. Women Birth. 2021;34(1):14-21. 10.1016/j.wombi.2020.01.013.

212. Ghafili A, Gantare A, Lobet-Maris C, Gourdin M. Towards the Elaboration of a Non-Technical Skills Development Model for Midwives in Morocco. Healthcare (Basel). 2022;10(9):1683. 10.3390/healthcare10091683.

213. Louazi A, Frías-Osuna A, López-Martínez C, Moreno-Cámara S. Perceptions, Motivations, and Empowerment Strategies of Midwives in Rural and Remote Areas of Northern Morocco. Int J Environ Res Public Health. 2022;19(22):14992. 10.3390/ijerph192214992.

214. Rouahi N, Boucetta N, Boussaa S. Exploratory study of an e-mentoring professional coaching model of novice midwives in Morocco. Pan Afr Med J. 2022;41:253. 10.11604/pamj.2022.41.253.29226.

**Newborn/Neonatal Health** (28)

215. Khadir K, Benharbit B, Habibeddine S, Benchikhi H, Lakhdar H. [Treatment outcome for collodion babies: the experience of the dermatology department of Ibn Rochd Teaching Hospital, Casablanca]. Devenir des bébés collodions : expérience du service de dermatologie du CHU Ibn Rochd de Casablanca. Ann Dermatol Venereol. 2009;136(10):731-2. 10.1016/j.annder.2009.05.006.

216. Lahmiti S, Elhoudzi J, Aboussad A, Lahmiti S, Elhoudzi J, Aboussad A. Congenital chylothorax. Sci World J. 2009;9:431-4. 10.1100/tsw.2009.62.

217. Kabiri M, Barkat A, El Ajaje H, Allali N, Dafiri R, Lamdouar-Bouazzaoui N. Neonatal epididymo-orchitis caused by Pseudomonas aeruginosa. Cases J. 2010;3:44. 10.1186/1757-1626-3-44.

218. Maoulainine F, Lamrani A, Elidrissi N, Sorra N, Chabaa L, Jalal H, et al. [Impact of umbilical venous complications of catheterisation in the newborn]. Incidence des complications du cathétérisme veineux ombilical chez le nouveau-né. J de Pediatrie et de Pueric. 2012;25(6):316-21. 10.1016/j.jpp.2012.08.005.

219. Mekaoui N, Issef I, Kabiri M, Barkat A. Analgesic effect of 30% glucose, milk and non-nutritive sucking in neonates. J Pain Res. 2012;5:573-7. 10.2147/JPR.S30665.

220. Touhami Elouazzani F, Kabiri M, Karboubi L, Barkat A, Keswati J, Mrabet M. [Macrosomia: About 255 cases]. La macrosomie : à propos de 255 cas. J de Pediatrie et de Pueric. 2012;25(97-101). 10.1016/j.jpp.2011.12.003.

221. Younous S, Zarrouki Y, Boutbaoucht M, Mouaffak Y, El Idrissi K, Aboussair N, et al. Prune belly syndrome associated with full spectrum of VACTERL in a newborn. J Clin Neonatol. 2012;1(1):49-51. 10.4103/2249-4847.92234.

222. Chemsi M, Chahid I, Lehlimi M, Aalloula O, Zerouali K, Habzi A, et al. [Incidence of nosocomial bacterial infections in a neonatal intensive care unit: Analysis of risk factors. Children's hospital Abderrahim Harouchi, University Hospital Ibn Rochd, Casablanca, Morocco]. Incidence des infections bactériennes nosocomiales. Hôpital d’enfants Abderrahim Harouchi, CHU Ibn Rochd, Casablanca, Maroc. J de Pediatrie et de Pueric. 2013;26(1):11-8. 10.1016/j.jpp.2012.11.001.

223. Elguazzar S, Alaoui A, Izgua A. [Evaluation of the practice of transfusion in the anemia in preterm infants]. Évaluation de la pratique transfusionnelle dans l'anémie du prématuré. Rev Med Brux. 2013;34(1):4-11.

224. Oulmaati A, Hmami F, Bouharrou A. [The contribution of quinolones in neonates (about 21 cases)]. L’apport des quinolones en néonatologie (à propos de 21 cas). J de Pediatrie et de Pueric. 2013;26(4):193-7. 10.1016/j.jpp.2013.05.002.

225. Chahid N, Boudana S, Kabiri M, Mrabet M, Knouni H, Kharbach A, et al. [Fetal and neonatal impact of pregnancy-induced hypertension: Moroccan data]. Retentissement fœtal et néonatal de l’hypertension artérielle gravidique : données marocaines. J de Pediatrie et de Pueric. 2014;27(3):111-6. 10.1016/j.jpp.2014.04.005.

226. Fadil F, Lehlimi M, Chemsi M, Habzi A, Benomar S. [Neonatal adrenal hematoma: various modes of presentation]. Hématome surrénalien néonatal : diversité des modes de révélation. Arch Pediatr. 2014;21(9):990-4. 10.1016/j.arcped.2014.06.004.

227. Ratbi I, Fejjal N, Legendre M, Collot N, Amselem S, Sefiani A. Clinical and molecular findings in a Moroccan patient with popliteal pterygium syndrome: a case report. J Med Case Rep. 2014;8:471. 10.1186/1752-1947-8-471.

228. Noureddine E, Abdellatif B. Prevalence and determinants of low birth weight: A case-control study in Marrakesh (Morocco). Iran J Public Health. 2015;44(3):422-4.

229. Hassoune S, Bassel S, Nani S, Elbouri H, Zine K, Maaroufi A. [Maternal factors associated with low birth weight: case-control study in a Moroccan public hospital]. Les facteurs maternels associés au faible poids de naissance: étude cas-témoins dans un hôpital public marocain. Pan Afr Med J. 2015;20:303. 10.11604/pamj.2015.20.303.2659.

230. Hassoune S, Bassel S, Nani S, Maaroufi A. [Prevalence and associated factors of low birth weight in the provincial hospital of Mohammedia--Morocco]. Prévalence et facteurs associés au faible poids de naissance à la maternité de l'hôpital provincial de Mohammedia--Maroc. Tunis Med. 2015;93(7):440-4.

231. Boubkraoui M, Aguenaou H, Mrabet M, Barkat A. [Perinatal morbidity and mortality in twin pregnancies in a Moroccan level-3 maternity ward]. Morbimortalité périnatale dans les grossesses gémellaires dans une maternité marocaine de niveau 3. Pan Afr Med J. 2016;23:80. 10.11604/pamj.2016.23.80.8789.

232. El Hasbaoui B, Karboubi L, Benjelloun B. Newborn haemorrhagic disorders: About 30 cases. Pan Afr Med J. 2017;28:150. 10.11604/pamj.2017.28.150.13159.

233. Maoulainine F, Elbaz M, Elfaiq S, Boufrioua G, Elalouani F, Barkane M, et al. Therapeutic Hypothermia in asphyxiated neonates: Experience from neonatal intensive care unit of University Hospital of Marrakech. Int J Pediatr. 2017;2017:3674140. 10.1155/2017/3674140.

234. Saiad M, Kamili E, Aballa N. Treatment of Preduodenal Portal Vein. Iran J Pediatr. 2017;27(2):e5791. 10.5812/ijp.5791.

235. Radouani M, Barkat A, Youness T, Benkirane H, Aguenaou H, Mrabet M. Ambulatory management of uncomplicated jaundice for fullterm neonates in Morocco. 2017;175(11):1651. 10.1007/s00431-016-2785-8.

236. Abdellaoui H, Mahmoudi A, Tazi Charki M, Bouabdallah Y. Caecal duplication cyst: a rare cause of neonatal intestinal obstruction. BMJ Case Rep. 2018;2018:bcr2017222225. 10.1136/bcr-2017-222225.

237. Barkat A, Kharbach A, Barkat A. [Study of the relationship between childbirth conditions and the severity of perinatal asphyxia. National Reference Center of Rabat for Neonatology and Nutrition]. Étude de l’association entre les conditions de l’accouchement et la gravité de l’asphyxie périnatale. Centre national de référence pour la néonatalogie et la nutrition de Rabat. J de Pediatrie et de Pueric. 2019;32(2):90-8. 10.1016/j.jpp.2019.01.001.

238. El Qadiry R, Ouayad A, Nassih H, Bourrahouat A, Ait Sab I. Neonatal Cholestasis: A rare and unusual presentation of Pituitary Stalk Interruption Syndrome. Case Rep Endocrinol. 2021;2021:6161508. 10.1155/2021/6161508.

239. Elfane H, El-Jamal S, Sahel K, Mziwira M, El Ayachi M, Belahsen R. Study of the association of the nutritional profile of pregnant women with the birth weight of newborns in Morocco. Rocz Panstw Zakl Hig. 2021;72(4):427-34. 10.32394/rpzh.2021.0189.

240. Elaabsi M, Loukid M, Lamtali S. Socio-economic and cultural determinants of mothers and fathers for low birth weight newborns in the region of Marrakech (Morocco): A case-control study. PLoS One. 2022;17(6):e0269832. 10.1371/journal.pone.0269832.

241. Elfane H, El-Jamal S, Mziwira M, Barakat I, Sahel K, El Ayachi M, et al. Risk factors for low birth weight in El Jadida province, Morocco. Case-control study. Rocz Panstw Zakl Hig. 2022;73(2):209-14. 10.32394/rpzh.2022.0206.

242. Imad N, El Idrissi Slitine N, Alaoui S, Zalle I, Boumzebra D, Bennaoui F, et al. Thrombosis of the abdominal aorta in newborn: About two cases. J Neonatal Perinatal Med. 2022;15(1):187-93. 10.3233/NPM-200678.

**Nutrition** (16)

243. Belgnaoui S, Belahsen R. Nutrient intake and food consumption among pregnant women from an agricultural region of Morocco. Int J Food Sci Nutr. 2006;57(1-2):19-27. 10.1080/09637480500465261.

244. Zaida F, Sedki A, Laroche D, Lekouch N, Bouglé D. High cord blood TSH in Morocco: Iatrogenic hypothyroidism? Ann Endocrinol (Paris). 2006;67(1):39-41. 10.1016/s0003-4266%2806%2972538-0.

245. Zaida F, Bureau F, Guyot S, Sedki A, Lekouch N, Arhan P, et al. Iron availability and consumption of tea, vervain and mint during weaning in Morocco. Ann Nutr Metab. 2006;50(3):237-41. 10.1159/000091680.

246. El Hamdouchi A, El Kari K, Rjimati E, El Mzibri M, Mokhtar N, Aguenaou H. Does flour fortification with electrolytic elemental iron improve the prevalence of iron deficiency anaemia among women in childbearing age and preschool children in Morocco? Mediterr J Nutr Metab. 2013;6(1):73-8. 10.1007/s12349-012-0113-z.

247. Mochhoury L, R R, Kasouati J, Kabiri M, Barkat A. Body mass index, gestational weight gain, and obstetric complications in Moroccan population. J Pregnancy. 2013;2013:379461. 10.1155/2013/379461.

248. Karamanos B, Thanopoulou A, Anastasiou E, Assaad-Khalil S, Albache N, Bachaoui M, et al. Relation of the Mediterranean diet with the incidence of gestational diabetes. Eur J Clin Nutr. 2014;68(1):8-13. 10.1038/ejcn.2013.177.

249. Coullin P, Diatta A, Boufettal H, Feingold J, Leguern E, Candelier J. The involvement of the trans-generational effect in the high incidence of the hydatidiform mole in Africa. Placenta. 2015;36(1):48-51. 10.1016/j.placenta.2014.10.017.

250. Loudyi F, Kassouati J, Kabiri M, Chahid N, Kharbach A, Aguenaou H, et al. Vitamin D status in Moroccan pregnant women and newborns: Reports of 102 cases. Pan Afr Med J. 2016;24:170. 10.11604/pamj.2016.24.170.4782.

251. Stinca SA, M; Herter-Aeberli, I; Chabaa, L; Cherkaoui, M; El Ansari, N; Aboussad, A; Weibel, S; Zimmermann, MB. Moderate-to-Severe Iodine Deficiency in the "First 1000 Days" Causes More Thyroid Hypofunction in Infants Than in Pregnant or Lactating Women. J Nutr. 2017;147(4):589-95. 10.3945/jn.116.244665.

252. Lifschitz C, Miqdady M, Indrio F, Haddad J, Tawfik E, AbdelHak A, et al. Practices of introduction of complementary feeding and iron deficiency prevention in the Middle East and North Africa. J Pediatr Gastroenterol Nutr. 2018;67(4):538-42. 10.1097/MPG.0000000000002059.

253. Ouzennou N, Tikert K, Belkedim G, Jarhmouti F, Baali A. [Prevalence and social determinants of anemia in pregnant women in Essaouira Province, Morocco]. Prévalence et déterminants sociaux de l'anémie chez les femmes enceintes dans la Province d'Essaouira, Maroc. Sante Publique. 2018;30(5):737-45. 10.3917/spub.186.0737.

254. Ouzennou N, Amor H, Baali A. Socio-economic, cultural and demographic profile of a group of Moroccan anaemic pregnant women. Afr Health Sci. 2019;19(3):2654-9. 10.4314/ahs.v19i3.41.

255. Atalhi N, El Hamdouchi A, Barkat A, Elkari K, Hamrani A, El Mzibri M, et al. Combined consumption of a single high-dose vitamin A supplement with provision of vitamin A fortified oil to households maintains adequate milk retinol concentrations for 6 months in lactating Moroccan women. Appl Physiol Nutr Metab. 2020;45(3):275-82. 10.1139/apnm-2019-0116.

256. Kancherla V, Chadha M, Rowe L, Thompson A, Jain S, Walters D, et al. Reducing the burden of anemia and neural tube defects in low-and middle-income countries: An analysis to identify countries with an immediate potential to benefit from large-scale mandatory fortification of wheat flour and rice. Nutrients. 2021;13(1):244. 10.3390/nu13010244.

257. Taoudi F, Laamiri F, Barich F, Hasswane N, Aguenaou H, Barkat A. Study of the prevalence of obesity and its association with maternal and neonatal characteristics and morbidity profile in a population of Moroccan pregnant women. J Nutr Metab. 2021;2021:6188847. 10.1155/2021/6188847.

258. Rami A, Saeid N, El Mzibri M, El Kari K, Idrissi M, Lahmam H, et al. Prevalence of iodine deficiency among Moroccan women of reproductive age. Arch Public Health. 2022;80(1):147. 10.1186/s13690-022-00901-7.

**Other Non-Respiratory Viruses** (2)

259. Bentama I, Soussi I, Ghanimi Z, Riane S, Tligui H, Mdaghri Alaoui A, et al. [Epidemic of nosocomial infection by rotavirus in a neonatology service]. Épidémie d'infection nosocomiale à rotavirus au sein d'un service de néonatologie. Rev Med Brux. 2012;33(6):519-24.

260. Mansouri S, Mai S, Hassam B, Benzekri L. Bullous varicella in an immunocompetent infant. BMJ Case Rep. 2019;12(3):e229025. 10.1136/bcr-2018-229025.

**Parasitic Disease** (16)

261. Laghzaoui Boukaidi M, Bouhya S, Soummani A, Hermas S, Bennan O, Sefrioui O, et al. [Pelvic hydatid cyst: A report of eight cases]. Kystes hydatiques pelviens : à propos de huit cas. Gynecol Obstet Fertil. 2001;29(5):354-7. 10.1016/S1297-9589(01)00145-X.

262. El Mansouri B, Rhajaoui M, Sebti F, Amarir F, Laboudi M, Bchitou R, et al. [Seroprevalence of toxoplasmosis in pregnant women in Rabat, Morocco]. Séroprévalence de la toxoplasmose chez la femme enceinte dans la ville de Rabat au Maroc. Bull Soc Pathol Exot. 2007;100(4):289-90.

263. Laboudi M, El Mansouri B, Sebti F, Amarir F, Coppieters Y, Rhajaoui M. [Risk factors of a positive serological test for toxoplasmosis in a pregnant women in Morocco]. Facteurs de risque d’une sérologie toxoplasmique positive chez la femme enceinte au Maroc. Parasite. 2009;16(1):71-2. 10.1051/parasite/2009161071.

264. Rachad M, Fdili F, Slimani O, Chaara H, Bouguern H, Melhouf M. [Intraperitoneal rupture of a hydatid cyst during pregnancy: report of a rare case]. La rupture intra péritonéale d'un kyste hydatique au cours de la grossesse: à propos d'un cas rare. Pan Afr Med J. 2012;11:1. 10.11604/pamj.2012.11.1.531.

265. Laboudi M, Sadak A. Serodiagnosis of Toxoplasmosis: The effect of measurement of IgG avidity in pregnant women in Rabat in Morocco. Acta Trop. 2017;172:139-42. 10.1016/j.actatropica.2017.04.008.

266. Tlamcani Z, Yahyaoui G, Mahmoud M. Prevalence of immunity to toxoplasmosis among pregnant women in University Hospital Center Hassan II of FEZ city (Morocco). Acta Med Int. 2017;4(1):43-5.

267. Mouttaki T, Maksouri H, El Mabrouki J, Merino-Espinosa G, Fellah H, Itri M, Martin-Sanchez, J, et al. Concomitant visceral and localized cutaneous leishmaniasis in two Moroccan infants. Infect Dis Poverty. 2018;7(1):32. 10.1186/s40249-018-0413-8.

268. Ouzennou N, Boussaa S, Ben Alla S, Boumezzough A. Observational study to assess pregnant women's knowledge and behaviour related to toxoplasmosis in Essaouira province, Morocco. Asian Pac J Trop Med. 2019;12(2):87-90. 10.4103/1995-7645.250842.

269. Tazi S, Ouknane H, Lyagoubi M, Aoufi S. [Visceral leishmaniasis leading to macrophage activation syndrome]. Leishmaniose viscérale à l’origine d’un syndrome d’activation macrophagique. Med Mal Infect. 2019;49(4):289-91. 10.1016/j.medmal.2019.01.003.

270. Hoummadi L, Berrouch S, Amraouza Y, Adel A, Mriouch M, Soraa N, et al. Seroprevalence of toxoplasmosis in pregnant women of the Marrakech-Safi region, Morocco. Afr Health Sci. 2020;20(1):59-63. 10.4314/ahs.v20i1.10.

271. Laboudi M, Ait Hamou S, Mansour I, Hilmi I, Sadak A. The first report of the evaluation of the knowledge regarding toxoplasmosis among health professionals in public health centers in Rabat, Morocco. Trop Med Health. 2020;48:17. 10.1186/s41182-020-00208-9.

272. Ait Hamou S, Laboudi M. An analytical study on the awareness and practice relating toxoplasmosis among pregnant women in Casablanca, Morocco. BMC Public Health. 2021;21(1):507. 10.1186/s12889-021-10474-9.

273. Laboudi M, Taghy Z, Duieb O, Peyron F, Sadak A. Toxoplasma gondii seroprevalence among pregnant women in Rabat, Morocco. Trop Med Health. 2021;49(1):21. 10.1186/s41182-021-00311-5.

274. Felín M, Wang K, Moreira A, Grose A, Leahy K, Zhou Y, et al. Building Programs to Eradicate Toxoplasmosis Part I: Introduction and Overview. Curr Pediatr Rep. 2022;10(3):57-92. 10.1007/s40124-022-00269-w.

275. Felín M, Wang K, Moreira A, Grose A, Leahy K, Zhou Y, et al. Building Programs to Eradicate Toxoplasmosis Part IV: Understanding and Development of Public Health Strategies and Advances “Take a Village”. Curr Pediatr Rep. 2022;10(3):125-54.

276. Hattoufi K, Bissati K, Adlaoui E, Aguenaou H, Kharbach A, Barkat A. Awareness of toxoplasmosis among postpartum women: a cross-sectional study in Morocco. Pan Afr Med J. 2022;Apr 7(41):282. 10.11604/pamj.2022.41.282.31049.

**Preeclampsia/Eclampsia** (10)

277. El Youssoufi S, Nsiri A, Salmi S, Miguil M. [Liver rupture in peripartum: about 8 cases]. Rupture du foie en péripartum: à propos de huit cas. J Gynecol Obstet Biol Reprod (Paris). 2007;36(1):57-61. 10.1016/j.jgyn.2006.09.003.

278. Sabiri B, Moussalit A, Salmi S, El Youssoufi S, Miguil M. [Post-partum eclampsia: Epidemiology and prognosis]. L'éclampsie du post-partum : épidémiologie et pronostic. J Gynecol Obstet Biol Reprod (Paris). 2007;36(3):276-80. 10.1016/j.jgyn.2006.12.025.

279. Araqi-Houssaini A, Salmi S, Moussaid I, Guennoun M, Elyoussoufi S, Miguil M, et al. [Posterior reversible encephalopathy syndrome and eclampsia: A descriptive study of 13 cases in Morocco]. Syndrome d’encéphalopathie postérieure réversible et éclampsie : étude descriptive de 13 cas au Maroc. Rev Neurol (Paris). 2011;167(11):812-9. 10.1016/j.neurol.2011.03.007.

280. Mamouni N, Bougern H, Derkaoui A, Bendahou K, Fakir S, Bouchikhi C, et al. HELLP syndrome: report of 61 cases and literature review. Le HELLP syndrome: à propos de 61 cas et revue de la littérature. Pan Afr Med J. 2012;11:30.

281. Zidouh S, Belyamani L, Kouach J, Drissi Kamili N. Subcapsular hepatic hematoma revealed by hemorrhagic shock in a preeclamptic patient. J Emerg Med. 2012;42(5):585-6. 10.1016/j.jemermed.2010.05.091.

282. Djoubairou B, Onen J, Doleagbenou A, El Fatemi N, Maaqili M. Chronic subdural haematoma associated with pre-eclampsia: case report and review of the literature. Neurochirurgie. 2014;60(1-2):48-50. 10.1016/j.neuchi.2013.11.002.

283. Handor H, Daoudi R. Images in clinical medicine. Hypertensive retinopathy associated with preeclampsia. N Engl J Med. 2014;370(8):752-. 10.1056/NEJMicm1306891.

284. Bentata Y, Madani H, Berkhli H, Saadi H, Mimouni A, Housni B. Complications and maternal mortality from severe pre-eclampsia during the first 48 hours in an intensive care unit in Morocco. Int J Gynaecol Obstet. 2015;129(2):175-6. 10.1016/j.ijgo.2014.11.013.

285. Benfateh M, Cissoko S, Boufettal H, Feige J, Samouh N, Aboussaouira T, et al. Risk factors and poor prognostic factors of preeclampsia in Ibn Rochd University Hospital of Casablanca: About 401 preeclamptic cases. Pan Afr Med J. 2018;31:225. 10.11604/pamj.2018.31.225.14401.

286. Rebahi H, Elizabeth Still M, Faouzi Y, Rhassane El Adib A. Risk factors for eclampsia in pregnant women with preeclampsia and positive neurosensory signs. Turk J Obstet Gynecol. 2018;15(4):227-34. 10.4274/TJOD.22308.

**Pregnancy** (24)

287. Soumani A, Salah-Eddine A, Bouhya S, Hermas S, Samouh N, Himmi A, et al. [Premature rupture of fetal membranes. Its management is still disputed!]. La rupture prématurée des membranes. Une prise en charge encore controversée! Tunis Med. 2000;78(2):90-100.

288. Berrada R, Bouguerne H, Ettayebi Z, Ouzeddoun N, Balafrej L, Benchekroun A, et al. [Renal transplantation and pregnancy. About one case]. Transplantation rénale et grossesse. À propos d'un cas. Ann Urol (Paris). 2001;35(4):193-7. 10.1016/S0003-4401%2801%2900035-3.

289. Benchikhi H, Razoki H, Lakhdar H. [Sunscreens: Use in pregnant women at Casablanca]. Photoprotecteurs externes. Utilisation chez la femme enceinte à Casablanca. Ann Dermatol Venereol. 2002;129(4):387-90.

290. Kadri N, Berrada S, Alami K, Manoudi F, Rachidi L, Maftouh S, et al. Mental health of Moroccan women, a sexual perspective. J Affect Disord. 2007;102(1-3):199-207. 10.1016/j.jad.2006.09.028.

291. Ezzouine H, Charra B, Benslama A, Motaouakkil S, Sodqi M. [A case of tuberculous meningitis in pregnancy]. Un cas de méningite tuberculeuse et grossesse. Med Mal Infect. 2008;38(1):36-7. 10.1016/j.medmal.2007.11.002.

292. Boufettal H, Majdi F, Belhouss A, Mahdaoui S, Noun M, Hermas S, et al. [Domestic violence during pregnancy]. Violence conjugale chez les femmes enceintes. Rev de Med Leg. 2012;3(2):72-8. 10.1016/j.medleg.2012.03.001.

293. Boufettal H, Obaid B, Belhouss A, Hermas S, Noun M, Samouh N. [Physical violence during pregnancy in Morocco]. Sévices physiques durant la grossesse au Maroc. J Gynecol Obstet Biol Reprod (Paris). 2012;41(1):76-82. 10.1016/j.jgyn.2011.06.017.

294. Oukkache B, El Graoui O, Zafad S. Combined factor V and VIII deficiency and pregnancy. Int J Hematol. 2012;96(6):786-8. 10.1007/s12185-012-1201-z.

295. El Hamdani F, Vimard P, Baali A, Zouini M, Cherkaoui M. [Prenatal care in the city of Marrakech]. Soins prénatals dans la ville de Marrakech. Med Sante Trop. 2013;23(2):162-7. 10.1684/mst.2013.0185.

296. Hicham S, Ihsane M, Abderahim el B, Brahim B, Labib S, Mustapha H, et al. Multivisceral organ failure related to leptospirosis in pregnant patient. Indian J Crit Care Med. 2013;17(1):43-5. 10.4103/0972-5229.112143.

297. Mansouri S, Abourazzak F, Almoubakir H, Rabhi H, Tahiri L, Banani A, et al. Reflex sympathetic dystrophy involving the hip in pregnancy: A case report and a review of the literature. Egypt Rheumatol. 2013;35(4):245-7. 10.1016/j.ejr.2013.03.002.

298. Slimani O, Jayi S, Fdili Alaoui F, Bouguern H, Chaara H, Fikri G, et al. An aggressive vertebral hemangioma in pregnancy: a case report. J Med Case Rep. 2014;8(1):207-. 10.1186/1752-1947-8-207.

299. El Ammouri A, Sabir M, Ahid S, Toufiq J, El Omari F. [Non-drug psycho-active substance use and pregnancy]. Grossesse et usage de substances psycho-actives non médicamenteuses. Encephale. 2015;41(4):302-8. 10.1016/j.encep.2014.10.014.

300. Samali M, Elkoundi A, Tahri A, Bensghir M, Haimeur C. Anesthetic management of spontaneous cervical epidural hematoma during pregnancy: A case report. J Med Case Rep. 2017;11(1):171. 10.1186/s13256-017-1335-y.

301. Yasmina A, Barakat A. [Prelabour rupture of membranes (PROM) at term: Prognostic factors and neonatal consequences]. Rupture prématurée des membranes à terme: facteurs pronostiques et conséquences néonatales. Pan Afr Med J. 2017;26:68. 10.11604/pamj.2017.26.68.11568.

302. Bakrim S, Motiaa Y, Ouarour A, Masrar A. Hematological parameters of the blood count in a healthy population of pregnant women in the northwest of Morocco (Tetouan-M'diq-Fnideq provinces). Pan Afr Med J. 2018;29:205. 10.11604/pamj.2018.29.205.13043.

303. Hassane M, Benkirane S, Motiaa Y, Dahmani F, Elkhorassani M, Masrar A. Profile of Von Willebrand factor antigen in pregnancy: Descriptive study of 390 pregnant women in Morocco. Pan Afr Med J. 2018;31:232. 10.11604/pamj.2018.31.232.13138.

304. El Jaouhari S, Doghmi N, Najout H, El Hamouni M, Kabiri E, Bekkali H, et al. Acute respiratory failure secondary to a cervical goitre in a pregnant woman: A case report. BMC Emerg Med. 2019;19(1):18. 10.1186/s12873-019-0231-8.

305. Guennoun S, Guennoun A, Krimou Y, Gounain F, Mamouni N, Errarhay S, et al. Adnexal torsion and pregnancy: About 3 cases. Eur J Obstet Gynecol. 2019;234:e208. 10.1016/j.ejogrb.2018.08.089.

306. Slaoui A, Talib S, Nah A, Moussaoui K, Benzina I, Zeraidi N, et al. Placenta accreta in the department of gynaecology and obstetrics in Rabat, Morocco: Case series and review of the literature. Pan Afr Med J. 2019;33:86. 10.11604/pamj.2019.33.86.17700.

307. Zaidi H, Lamalmi N, Malihi A, Barkat A, Alhamany Z. [Histopathological lesions of the placenta associated with severe intrauterine growth retardation: About a clinical case]. Les lésions histopathologiques du placenta au cours du retard de croissance intra utérine sévère: à propos d'un cas clinique. Pan Afr Med J. 2019;34:56. 10.11604/pamj.2019.34.56.15160.

308. Elkoundi A, Mounir K, Atmani W, Balkhi H. Management of a patient with Shapiro syndrome variant during pregnancy. Int J Obstet Anesth. 2020;43:87-8. 10.1016/j.ijoa.2020.03.004.

309. Soule H, Jayi S, Madi T, Conte A, Alaoui F, Chaara H, et al. Decidualization of an ovarian endometrioma complicated by a sigmoid fistula during pregnancy: a case report. J Med Case Rep. 2020;14(1):198. 10.1186/s13256-020-02513-7.

310. Chergaoui S, Changuiti O, Marfak A, Saad E, Hilali A, Youlyouz Marfak I. Modern drug self-medication and associated factors among pregnant women at Settat city, Morocco. Front Pharmacol. 2022;Aug 16(13):812060. 10.3389/fphar.2022.812060.

**Pregnancy--ectopic** (4)

311. Mahi M, Boumdin H, Chaouir S, Salaheddine T, Attioui D, Amil T, et al. [A new case of abdominal pregnancy]. Un nouveau cas de grossesse abdominale. J Radiol. 2002;83(7-8):989-92.

312. Siati A, Berrada T, Baidada A, Kharbach A. Abdominal pregnancy with a healthy newborn: A new case. Pan Afr Med J. 2019;34:35. 10.11604/pamj.2019.34.35.20169.

313. Elmiski F, Ouafidi B, Elazzouzi E, Elquasseh R, Lamrissi A, Fichtali K, et al. Abdominal pregnancy diagnosed by ultrasonography and treated successfully by laparotomy: Two cases report. Int J Surg Case Rep. 2021;83:105952. 10.1016/j.ijscr.2021.105952.

314. Bouab M, Touimi A, Jalal M, Lamrissi A, Fichtali K, Bouhya S. Diagnosis and management of ectopic ovarian pregnancy: a rare case report. Int J Surg Case Rep. 2022;91:106742. 10.1016/j.ijscr.2021.106742.

**Pregnancy--heterotopic** (3)

315. Laghzaoui Boukaidi M, Bouhya S, Sefrioui O, Bennani O, Hermas S, Aderdour M. [Heterotopic pregnancies: 8 cases]. Grossesses hétérotopiques : à propos de huit cas. Gynecol Obstet Fertil. 2002;30(3):218-23. 10.1016/s1297-9589(02)00298-9.

316. Guennoun A, Mamouni N, Errarhay S, Bouchikhi C, Banani A. [Spontaneous heterotopic pregnancy: about 2 cases]. La grossesse hétérotopique spontanée: à propos de deux cas. Pan Afr Med J. 2017;28:306. 10.11604/pamj.2017.28.306.13696.

317. Ouafidi B, Kiram H, Benaguida H, Lamrissi A, Fichtali K, Bouhya S. Diagnosis and management of a spontaneous heterotopic pregnancy: Rare case report. Int J Surg Case Rep. 2021;84:106184. 10.1016/j.ijscr.2021.106184.

**Pregnancy--hydatiform mole** (6)

318. Boufettal H, Coullin P, Mahdaoui S, Noun M, Hermas S, Samouh N. [Complete hydatiforme mole in Morocco: Epidemiological and clinical study]. Les môles hydatiformes complètes au Maroc : étude épidémiologique et clinique. J Gynecol Obstet Biol Reprod (Paris). 2011;40(5):419-29. 10.1016/j.jgyn.2011.02.008.

319. Boufettal H, Coullin P, Mahdaoui S, Noun M, Hermas S, Samouh N. [Complete hydatiforme mole in Morocco: Epidemiological and clinical study]. Les môles hydatiformes partielles au Maroc : étude épidémiologique et clinique. East Mediterr Health J. 2012;18(7):755-61. 10.26719/2012.18.7.755.

320. Bousfiha N, Erarhay S, Louba A, Saadi H, Bouchikhi C, Banani A, et al. Ectopic molar pregnancy: a case report. Pan Afr Med J. 2012;11:63.

321. Zohoun A, Hounmenou K, Mechtani Sel I, Razine R, Dami A, Filali A, et al. [Utility of hCG dosage in the management of gestational trophoblastic diseases]. Intérêt du dosage de l'hormone chorionique gonadotrope dans les maladies trophoblastiques gestationnelles. Ann Biol Clin (Paris). 2013;71(6):639-43. 10.1684/abc.2013.0908.

322. El Miski F, Benjelloun A, Bouab M, Lamrissi A, Fichtali K, Bouhya S. Spontaneous uterine rupture during the first trimester of a partial molar pregnancy in a scar uterus: A rare case report. Int J Surg Case Rep. 2021;85:106229. 10.1016/j.ijscr.2021.106229.

323. Slaoui A, Kharbach A, Baydada A. Hydatidiform mole in a 39-year-old woman. CMAJ. 2022;194(35):E1215. 10.1503/cmaj.220185.

**Public Information/Literacy** (2)

324. Gueddari W, Tazi A, Ouardi A, Nani S, Zineddine A. [Evaluation of parental knowledge on acute bronchiolitis]. Évaluation des connaissances des parents sur les bronchiolites aiguës. Pan Afr Med J. 2014;17:310. 10.11604/pamj.2014.17.310.2717.

325. Ouasmani F, Engeltjes B, Haddou Rahou B, Belayachi O, Verhoeven C. Knowledge of hypertensive disorders in pregnancy of Moroccan women in Morocco and in the Netherlands: A qualitative interview study. BMC Pregnancy Childbirth. 2018;18(1):344. 10.1186/s12884-018-1980-1.

**Respiratory Virus** (7)

326. Adnane Berdai M, S L, Harandou M. [Severe forms of influenza A (H1N1) 2009 in pregnant women: experience of the University Hospital of Fez, Morocco and literature review]. Les formes graves de la grippe A (H1N1) 2009 chez la femme enceinte: expérience du centre hospitalier universitaire de Fès, Maroc et revue de la littérature. Pan Afr Med J. 2012;11:36.

327. Ait Addi R, Benksim A, Cherkaoui M. Pregnancy and COVID-19: What we need to know. Electron J Gen Med. 2020;17(6):em228. 10.29333/ejgm/7891.

328. Hattoufi K, Tligui H, Obtel M, El Ftouh S, Kharbach A, Barkat A. Molecular diagnosis of pneumonia using multiplex real-time PCR assay RespiFinder SMART 22 FAST in a group of Moroccan infants. Adv Virol. 2020;2020:6212643. 10.1155/2020/6212643.

329. Katfy K, Diawara I, Maaloum F, Aziz S, Guiso N, Fellah H, et al. Pertussis in infants, in their mothers and other contacts in Casablanca, Morocco. BMC Infect Dis. 2020;20(1):43. 10.1186/s12879-019-4680-1.

330. Aasfara J, Hajjij A, Bensouda H, Ouhabi H, Benariba F. A unique association of bifacial weakness, paresthesia and vestibulocochlear neuritis as post-covid-19 manifestation in pregnant women: A case report. Pan Afr Med J. 2021;38:30. 10.11604/pamj.2021.38.30.27646.

331. Benlghazi A, Benali S, Bouhtouri Y, Belouad M, Massoudi H, Kouach J. [Sars-cov-2 infection in pregnant women; epidemiological, clinical, biological and evolutionary profile in 16 cases: The covid-19 experience in the Moroccan military hospital in Benslimane]. Infection SARS-CoV-2 chez la femme enceinte; profil épidémiologique, clinique, biologique et évolutifs, à propos de 16 cas: expérience de l’Hôpital Militaire Marocain COVID-19 de Benslimane. Pan Afr Med J. 2021;38:384. 10.11604/pamj.2021.38.384.28695.

332. Ouahid H, Adarmouch L, Soummani A, Cherkaoui M, Sebbani M, Amine M. [Reorganization of a third-level maternity ward during the COVID-19 pandemic: maternity ward experience at the University Hospital of Marrakech]. Expérience de réorganisation d'une maternité de troisième niveau face à la pandémie de la COVID-19: étude du cas de la maternité de l´hôpital universitaire de Marrakech. Pan Afr Med J. 2022;41:38. 10.11604/pamj.2022.41.38.26186.

**Rural/Amazigh** (5)

333. Zouini M, Cherkaoui M, Baali A, Amor H, Hilali M, El Hamdani F, et al. [Obstetric care: Supply and demand care in three valleys of the western High Atlas region of Morocco (Anougal, Azgour and Imnane)]. Demande et offre de soins obstétricaux dans le Haut Atlas occidental. Med Sante Trop. 2010;29(1):110-1. 10.1684/san.2010.0213.

334. Sebbani M, Adarmouch L, Azzahiri I, Quiddi W, Cherkaoui M, Amine M. [Knowledge and attitudes towards reproductive health: Survey among Moroccans living in rural areas]. Connaissances et comportements au regard de la santé reproductive: enquête chez les marocains en zone rurale. Pan Afr Med J. 2016;25:186. 10.11604/pamj.2016.25.186.9940.

335. Sebbani M, Adarmouch L, Amine M, Cherkaoui M. [Community mobilization for the improvement of monitoring in pregnant women living in rural areas in Morocco]. Mobilisation communautaire pour l'amélioration de la surveillance de la grossesse en milieu rural au Maroc. Pan Afr Med J. 2020;35:73. 10.11604/pamj.2020.35.73.18328.

336. Sebbani M, Adarmouch L, Amine M, Cherkaoui M. Determinants of maternal health services use among vulnerable women in a rural community in the Moroccan High Atlas. Afr J Reprod Health. 2020;24(1):97-105. 10.29063/ajrh2020/v24i1.10.

337. Baayd J, Simonsen S, Stanford J, Willis S, Frost C. Identifying barriers to accessing skilled maternal health care in rural Morocco. Afr J Reprod Health. 2021;25(1):20-8. 10.29063/ajrh2021/v25i1.3.

**Screening for Newborns** (7)

338. Belahcen A, Taloubi M, Chala S, Izgua A, Alaoui A. Mother's awareness and attitudes towards prenatal screening for Down Syndrome in Muslim Moroccans. Prenat Diagn. 2014;34(9):821-30. 10.1002/pd.4373.

339. Hamzi K, Itto A, Itri M, Nadifi S. Prenatal diagnosis of BMD in Morocco: evolution and limits. J Mol Neurosci. 2014;52(4):459-60. 10.1007/s12031-013-0106-5.

340. Oulmaati A, Hmami F, Hida M, Bouharrou A. [High prevalence of hypothyroidism among children hospitalized in a neonatal intensive care unit in Morocco]. L'hypothyroïdie congénitale est une cause fréquente d'hospitalisation en réanimation néonatale au Maroc. Arch Pediatr. 2016;23(1):105-6. 10.1016/j.arcped.2015.10.010.

341. Maniar S, Amor C, Bijjou A. [Screening of congenital hypothyroidism in Morocco: A pilot study]. Dépistage de l’hypothyroïdie congénitale au Maroc : étude pilote. East Mediterr Health J. 2018;24(11):1066-73.

342. El Idrissi Slitine N, Bennaoui F, Sable C, Martin G, Hom L, Fadel A, et al. Pulse oximetry and congenital heart disease screening: Results of the first pilot study in Morocco. Int J Neonatal Screen. 2020;6(3):53. 10.3390/IJNS6030053.

343. Hom L, Martin G. Newborn critical congenital heart disease screening using pulse oximetry: Value and unique challenges in developing regions. Int J Neonatal Screen. 2020;6(3):74. 10.3390/IJNS6030074.

344. Therrell BJ, Lloyd-Puryear M, Ohene-Frempong K, Ware R, Padilla C, Ambrose E, et al. Empowering newborn screening programs in African countries through establishment of an international collaborative effort. J Community Genet. 2020;11(3):253-68. 10.1007/s12687-020-00463-7.

**Social Determinants of Health** (5)

345. Abdesslam B. Social Determinants of Reproductive Health in Morocco. Afr J Reprod Health. 2011;15(2):57-66.

346. Boutayeb W, Lamlili M, Maamri A, Ben El Mostafa S, Boutayeb A. Actions on social determinants and interventions in primary health to improve mother and child health and health equity in Morocco. Int J Equity Health. 2016;15:19. 10.1186/s12939-016-0309-9.

347. Akseer N, Kamali M, Bakhache N, Mirza M, Mehta S, Al-Gashm S, et al. Status and drivers of maternal, newborn, child and adolescent health in the Islamic world: a comparative analysis. Lancet. 2018;391(10129):1493-512. 10.1016/S0140-6736%2818%2930183-1.

348. Rinker C. Problematizing neoliberalism and development: Creating citizens (and future citizens) through reproduction and childrearing in Morocco. Hespéris-Tamuda. 2020;4:151-72.

349. Drioui C, Bakass F. Gender inequalitities and fertility in Morocco: Measuring Women's empowerment and impact on the ideal number of children. J Popul Soc Stud. 2021;29:325-50. 10.25133/JPSSv292021.021.

**Technology** (7)

350. Chandani Y, Breton G. Contraceptive security, information flow, and local adaptations: Family planning Morocco. Afr Health Sci. 2001;1(2):73-82.

351. Bachiri M, Idri A, Fernández-Alemán J, Toval A. Evaluating the Privacy Policies of Mobile Personal Health Records for Pregnancy Monitoring. J Med Syst. 2018;42(8):144. 10.1007/s10916-018-1002-x.

352. El Hasbaoui B, Karboubi L, Benjelloun B. The role of abdominal ultrasound in the management of excessive crying in infants. Pan Afr Med J. 2018;30:68. 10.11604/pamj.2018.30.68.12058.

353. Lykins J, Li X, Levigne P, Zhou Y, El Bissati K, Clouser F, et al. Rapid, inexpensive, fingerstick, whole-blood, sensitive, specific, point-of-care test for anti-Toxoplasma antibodies. PLoS Negl Trop Dis. 2018;12(8):e0006536. 10.1371/journal.pntd.0006536.

354. Ouhenach M, Zrhidri A, Jaouad I, Smaili W, Sefiani A. Application of next generation sequencing in genetic counseling a case of a couple at risk of cystinosis. BMC Med Genet. 2020;21(1):240. 10.1186/s12881-020-01167-y.

355. Kharbouch M, Idri A, Rachad T, Alami H, Redman L, Stelate Y. Mobile technology for improved contraceptive care in Morocco. J Med Syst. 2021;45(2):16. 10.1007/s10916-020-01684-6.

356. Sabir S, Rokhssi H, Famma N, Azhari M, Bentahar O. Prefabricated Trays Stock for Impression of Cleft lip and Palate Orthopedic Appliances: A Three-Dimensional Computational Analysis of Maxillary Jaws. Cleft Palate Craniofac J. 2022;Aug 9:Online ahead of print. 10.1177/10556656221119075.

**The Healthcare system** (20)

357. Indicators AWGo. Program note: Using UN process indicators to assess needs in emergency obstetric services: Morocco, Nicaragua and Sri Lanka. Int J Gynaecol Obstet. 2003;80(2):222-30. 10.1016/S0020-7292%2802%2900390-9.

358. Hotchkiss D, Krasovec K, El-Idrissi M, Eckert E, Karim A. The role of user charges and structural attributes of quality on the use of maternal health services in Morocco. Int J Health Plann Manage. 2005;20(2):113-35. 10.1002/hpm.802.

359. Fauveau V, Donnay F. Can the process indicators for emergency obstetric care assess the progress of maternal mortality reduction programs? An examination of UNFPA Projects 2000-2004. Int J Gynaecol Obstet. 2006;93(3):308-16. 10.1016/j.ijgo.2006.01.031.

360. Couillet M, Serhier Z, Tachfouti N, Elrhazi K, Nejjari C, Perez F. The use of antenatal services in health centres of Fes, Morocco. J Obstet Gynaecol. 2007;27(7):688-94. 10.1080/01443610701629080.

361. Muffler N, Trabelssi Mel H, De Brouwere V. Scaling up clinical audits of obstetric cases in Morocco. Trop Med Int Health. 2007;12(10):1248-57. 10.1111/j.1365-3156.2007.01911.x.

362. Fernandez M, Coeytaux F, Ponce de León R, Harrison D. Assessing the global availability of misoprostol. Int J Gynaecol Obstet. 2009;105(2):180-6. 10.1016/j.ijgo.2008.12.016.

363. Bennis I, De Brouwere V. Fee exemption for caesarean section in Morocco. Arch Public Health. 2012;70(1):3. 10.1186/0778-7367-70-3.

364. Marchal B, Van Belle S, De Brouwere V, Witter S. Studying complex interventions: reflections from the FEMHealth project on evaluating fee exemption policies in West Africa and Morocco. BMC Health Serv Res. 2013;13:469. 10.1186/1472-6963-13-469.

365. Bartlett L, Weissman E, Gubin R, Patton-Molitors R, Friberg I. The impact and cost of scaling up midwifery and obstetrics in 58 low- and middle-income countries. PLoS One. 2014;9(6):e98550. 10.1371/journal.pone.0098550.

366. Boukhalfa C, Abouchadi S, Cunden N, Witter S. The free delivery and caesarean policy in Morocco: how much do households still pay? Trop Med Int Health. 2016;21(2):245-52. 10.1111/tmi.12638.

367. Witter S, Boukhalfa C, Cresswell J, Daou Z, Filippi V, Ganaba R, et al. Cost and impact of policies to remove and reduce fees for obstetric care in Benin, Burkina Faso, Mali and Morocco. Int J Equity Health. 2016;15(1):123. 10.1186/s12939-016-0412-y.

368. Elkhoudri N, Baali A, Amor H. Postnatal Care: Levels and Determinants in Morocco. Iran J Public Health. 2017;46(2):242-8.

369. Witter S, Ilboudo P, Cunden N, Boukhalfa C, Makoutode P, Daou Z. Delivery fee exemption and subsidy policies: how have they affected health staff? Findings from a four-country evaluation. Health Policy Plan. 2017;32(2):236-47. 10.1093/heapol/czw116.

370. Van der Veken K, Dkhimi F, Marchal B, Decat P. They are after quantity, not quality: Health providers' perceptions of fee exemption policies in Morocco. Int J Health Policy Manag. 2018;7(12):1110-9. 10.15171/ijhpm.2018.76.

371. Ouakhzan B, Boukhalfa C. Application of the by the activity-based costing (ABC) method in a clinical department: example of the maternity unit of the Tiznit Provincial Hospital Center. Med Sante Trop. 2019;29(3):322-6. 10.1684/mst.2019.0921.

372. Assarag B, Sanae E, Rachid B. Priorities for sexual and reproductive health in Morocco as part of universal health coverage: maternal health as a national priority. Sex Reprod Health Matters. 2020;28(2):1845426. 10.1080/26410397.2020.1845426.

373. Hasan M, Magalhaes R, Ahmed S, Ahmed S, Biswas T, Fatima Y, et al. Meeting the global target in reproductive, maternal, newborn, and child health care services in low- and middle-income countries. Glob Health Sci Pract. 2020;8(4):654-65. 10.9745/GHSP-D-20-00097.

374. Kabakian-Khasholian T, Quezada-Yamamoto H, Ali A, Sahbani S, Afifi M, Rawaf S, et al. Integration of sexual and reproductive health services in the provision of primary health care in the Arab States: status and a way forward. Sex Reprod Health Matters. 2020;28(2):1773693. 10.1080/26410397.2020.1773693.

375. Ammerdorffer A, Laws M, Awiligwe A, Erb F, Im-Amornphong W, Gülmezoglu A, et al. Regulatory standards and processes for over-the-counter availability of hormonal contraception and drugs for medical abortion in five countries in the Eastern Mediterranean Region. Health Res Policy Syst. 2021;19(Suppl 1):51. 10.1186/s12961-020-00661-2.

376. Bezad R, Omrani S, Benbella A, Assarag B. Access to infertility care services towards Universal Health Coverage is a right and not an option. BMC Health Serv Res. 2022;22(1):1089. 10.1186/s12913-022-08456-7.

**Traditional medicine** (20)

377. Lekouch N, Sedki A, Nejmeddine A, Gamon S. Lead and traditional Moroccan pharmacopoeia. Sci Total Environ. 2001;280(1-3):39-43. 10.1016/S0048-9697(01)00801-4.

378. Khalki L, M'hamed S, Bennis M, Chait A, Sokar Z. Evaluation of the developmental toxicity of the aqueous extract from Trigonella foenum-graecum (L.) in mice. J Ethnopharmacol. 2010;131(2):321-5. 10.1016/j.jep.2010.06.033.

379. Achour S, Abourazzak S, Mokhtari A, Soulaymani A, Soulaymani R, Hida M. Juniper tar (cade oil) poisoning in newborn after a cutaneous application. BMJ Case Rep. 2011;2011:bcr0720114427. 10.1136/bcr.07.2011.4427.

380. Achour S, Rhalem N, Khattabi A, Lofti H, Mokhtari A, Soulaymani A, et al. [Peganum harmala L. poisoning in Morocco: about 200 cases]. L'intoxication au Peganum harmala L. au Maroc : à propos de 200 cas. Therapie. 2012;67(1):53-8. 10.2515/therapie/2012003.

381. Achour S, Saadi H, Turcant A, Banani A, Mokhtari A, Soulaymani A, et al. [Peganum harmala L. poisoning and pregnancy: two cases in Morocco]. Intoxication au Peganum harmala L. et grossesse : deux observations marocaines. Med Sante Trop. 2012;22(1):84-6. 10.1684/mst.2012.0013.

382. Khalki L, Bennis M, Sokar Z, Ba-M'hamed S. The developmental neurobehavioral effects of fenugreek seeds on prenatally exposed mice. J Ethnopharmacol. 2012;139(2):672-7. 10.1016/j.jep.2011.12.011.

383. Said A, Derfoufi S, Benmoussa A, Sbai I. Ethnopharmacological survey of traditional medicinal plants used for the treatment of infantile colic in Morocco. J Chem Pharm Res. 2015;7(7):664-71.

384. Elkhoudri N, Baali A, Amor H. Maternal morbidity and the use of medicinal herbs in the city of Marrakech, Morocco. Indian J Tradit Knowl. 2016;15(1):79-85.

385. Oulmaati A, Hmami F, Achour S, Bouharrou A. [Severe poisoning by traditional medication in the newborn]. Intoxications graves par médication traditionnelle chez le nouveau-né. Arch Pediatr. 2017;24(9):833-6. 10.1016/j.arcped.2017.06.005.

386. Teixidor-Toneu I, Martin G, Puri R, Ouhammou A, Hawkins J. Treating infants with frigg: linking disease aetiologies, medicinal plant use and care-seeking behaviour in southern Morocco. J Ethnobiol Ethnomed. 2017;13(1):4. 10.1186/s13002-016-0129-4.

387. El Kamari F, Taroq A, Atki Y, Aouam I, Lyoussi B, Abdellaoui A. Chemical composition of essential oils from vitex agnus-castus L. Growing in Morocco and its in vitro antibacterial activity against clinical bacteria responsible for nosocomial infections. Asian J Pharm Clin Res. 2018;11(10):365-8. 10.22159/ajpcr.2018.v11i10.27307.

388. El Kamari F, Taroq A, El Atki Y, Aouam I, Oumokhtar B, Lyoussi B, et al. Cymbopogon nardus L. Essential oil: Phytochemical screening and its antibacterial activity against clinical bacteria responsible for nosocomial infections in neonatal intensive care. Int J Pharm Sci. 2018;50(1):14-7.

389. Laadraoui J, Aboufatima R, El Gabbas Z, Ferehan H, Bezza K, Ait Laaradia M, et al. Effect of Artemisia herba-alba consumption during pregnancy on fertility, morphological and behaviors of mice offspring. J Ethnopharmacol. 2018;226:105-10. 10.1016/j.jep.2018.08.017.

390. Taroq A, Kamari F, Oumokhtar B, Aouam I, Atki Y, Lyoussi B, et al. Phytochemical Screening of the Essential Oil of Syzygium aromaticum and Antibacterial Activity against Nosocomial Infections in Neonatal Intensive Care. Int J Pharm Sci Rev Res. 2018;48(1):58-61.

391. Hoummani H, Sahli M, Chebat A, Hida M, Achour S. [Four fatal child Juniper tar (cade oil) poisoning]. Intoxication infantile au juniperus oxycedrus (huile de cade) : à propos de 4 décès. Toxicol. 2019;31(2):129-33. 10.1016/j.toxac.2019.02.003.

392. Lakhdar K, Berdai M, Benlamkadem S, Labib S, Harandou M. Cade oil poisoning: about a case. Med Sante Trop. 2019;29(1):110-1. 10.1684/mst.2018.0843.

393. Taroq A, El Kamari F, Aouam I, El Atki Y, Lyoussi B, Abdellaoui A. Phytochemical screening, polyphenols content and a novel source of antibacterial and antioxidant activities of essential oil of Laurus nobilis from Morocco. Int J Pharm Sci. 2019;10(8):3770-6. 10.13040/IJPSR.0975-8232.10%288%29.3770-76.

394. Eddouks M, Hebi M, Ajebli M. Medicinal plants and gyneco-obstetric disorders among women in the South East of Morocco. Curr Womens Health Rev. 2020;16(1):2-17. 10.2174/1573404815666191206112518.

395. Slighoua M, Mahdi I, Amrati F, Boukhira S, Youbi A, Bari A, et al. Ethnopharmacological survey of medicinal plants used in the traditional treatment of female infertility in Fez Region, Morocco. Enquête ethnopharmacologique des plantes médicinales utilisées dans le traitement traditionnel de l’infertilité féminine dans la région de Fès, Maroc. Phytothérapie. 2020;18(5):321-39. 10.3166/phyto-2019-0194.

396. Kamel N, El Boullani R, Cherrah Y. Use of Medicinal Plants during Pregnancy, Childbirth and Postpartum in Southern Morocco. Healthcare (Basel). 2022;10(11):2327. 10.3390/healthcare10112327.

**Vaccination** (6)

397. Caidi H, Bloom S, Azilmaat M, Benjouad A, Reef S, El Aouad R. Rubella seroprevalence among women aged 15-39 years in Morocco. East Mediterr Health J. 2009;15(3):526-31.

398. Belefquih B, Kasouati J, Doblali T, Touil N, Tagajdid M, Kabbaj H, et al. Rubella seroprevalence in pregnant women at the military teaching hospital, Rabat, Morocco. Int J Gynaecol Obstet. 2013;120(2):191-2. 10.1016/j.ijgo.2012.08.026.

399. Lohiniva A, Barakat A, Dueger E, Restrepo S, El Aouad R. A qualitative study of vaccine acceptability and decision making among pregnant women in Morocco during the a (h1n1) pdm09 pandemic. PLoS One. 2014;9(10):e96244. 10.1371/journal.pone.0096244.

400. Sbiti M, Khalki H, Benbella I, Louzi L. [Seroprevalence of HBsAg in pregnant women in central Morocco]. Séroprévalence de l’AgHBs chez la femme enceinte dans le centre du Maroc. Pan Afr Med J. 2016;24:187. 10.11604/pamj.2016.24.187.9849.

401. Messaoudi S, Azizi M, Ayyad A, Amrani R. [Delayed vaccination in premature newborns formerly hospitalized in the neonatology and neonatal resuscitation department, CHU Mohammed VI Oujda, Morocco]. Le retard de la vaccination chez les nouveau-nés prématurés anciennement hospitalisés au service de néonatologie et de réanimation néonatale, CHU Mohammed VI Oujda, Maroc. J de Pediatrie et de Pueric. 2020;33(5):239-43. 10.1016/j.jpp.2020.06.004.

402. Zahir H, Arsalane L, Elghouat G, Mouhib H, Elkamouni Y, Zouhair S. Seroprevalence of rubella in pregnant women in Southern Morocco. Pan Afr Med J. 2020;35(Suppl 1):10. 10.11604/pamj.supp.2020.35.1.18496.
