# Supplementary material for: Mapping maternal and infant health in Morocco: A global scoping review of themes, gaps, and the "unseen" in the published health research literature, 2000–2022
Source: PLOS Glob Public Health. 2024 Jul 18;4(7):e0003488. doi: 10.1371/journal.pgph.0003488 (PMC11257357; doi:10.1371/journal.pgph.0003488)
Supplement: Attachment — Submitted filename: Amsteretal_responsetoreview.docx [file pgph.0003488.s017.docx]

Thank you for your thoughtful comments, please see our response below.

For clarity, we have created a table (see below) with the reviewer comments (*italics*), as well as our replies (bolded), and the locations of changes made to the manuscript itself.

Please note that the page numbers of changes listed here in this table will align only with the Tracked Changes version of the manuscript, specifically when ‘All Markup’ view is selected from the Review > Tracking pane in Microsoft Word.

**Reviewer Comments—Response and Edits:**

|  | **Reviewer Comment** | **Authors’ Response** | **Location of edits made in manuscript (track-changes version), listed by heading, sub-heading, and page number** |
| --- | --- | --- | --- |
| **Reviewer 1** | ***Note 1.*** *In introduction, the authors emphasize that gender equities are not represented in MIH. How can this be better explained when maternal health is all about adolescent and pregnant women?* | **As Reviewer 1 says, maternal health is all about women. Our intent was to highlight that women-centered work is important, because gender inequities are known to impact health outcomes (e.g., family gender dynamics as a barrier to care access, etc.) We here describe the calls of some recent MIH researchers for more women-centered work.**  **We have redrafted this section of the introduction to clarify the argument and provide greater clarity and emphasis to this point.** | **Introduction:**   - Page 5   **Discussion > Summary of Overall Findings—What is Known:**   - Page 29 |
| **Reviewer 1** | ***Note 2.*** *If the intention was to include only post-2000 articles, why was the search run from inception to current time.* | **We have added language to the Methods section to clarify and emphasize the rationale for the search to be run as it was. The rationale is as follows:**  **We ran the search expansively at first, to capture the entire evolution of MIH in Morocco. However, after reviewing all the articles, we decided that pre-2000 belongs to history and is less relevant for current health issues. The post-2000 period was deemed more relevant to the objectives of this review, because it reflects contemporary health challenges in Morocco and the Moroccan national policy priorities following the MDG declaration.**  **2000-2011 and 2012-2022 reflected current WHO policies and Moroccan experiences with healthcare, so we decided to focus there. Pre-2000 will be the subject of a future historical bibliography and article.**  **The PRISMA diagram reflects the original search and alerts the reader to the volume of research that exists pre-2000. We have added a note to the PRISMA diagram to let the reader know these pre-2000 articles will be used for a future historical project.** | **Methods > Scoping Review:**   - Page 6   **Results > Primary MIH Articles > Figure 1:**   - Page 10, PRISMA diagram |
| **Reviewer 1** | ***Note 3.*** *The following statement needs more clarity – “Five open-source datasets were used for map generation and geographical analyses of data [30-34].” What were the nature of these datasets and what sort of geographical analyses were planned?* | **We used open-source GIS mapping layers to create several maps, to visualize extracted location data/spatial data from full text articles.**  **We also used these open-source GIS mapping layers to create a “heat map” of health facility density in Morocco, to give readers an idea of the concentration and distribution of health facilities in Morocco. (Figure S4).**  **We have revised the language in the manuscript for greater clarity.** | **Methods > Analysis:**   - Page 8 |
| **Reviewer 1** | ***Note 4.*** *The authors have combined a qualitative approach to summarizing the content of articles identified within a scoping review framework. Although this a novel approach, it is undermining the strong methodological framework of pure qualitative research. For e.g., a conceptual or theoretical framework was not predefined, it is not clear whether inductive or deductive analysis was planned before the data extraction began, whether any saturation limit was set for theme identification, whether any pre-defined themes were not at all found in the literature.* | **We have revised the methods section to provide more detail about the qualitative methodologies we used for thematic coding of articles and to compose summaries of articles within a given theme.**    **For inductive thematic analysis of full-text articles, we used Braun and Clarke (2006, 2012, 2022). Full-text articles were first classified according to preliminary themes developed inductively from reading abstracts, then articles were organized into chronological bibliographies and subject to deep thematic content analysis by EA. Themes were revised and articles recategorized through inductive analysis until thematic saturation was reached; i.e., when authors EA and CR agreed that the 34 identified themes proved coherent and meaningful. There were no pre-defined themes.**    **For the qualitative summaries of articles under each theme presented in Table 2, EA used Sandelowski’s Qualitative Description to identify and synthesize the principal ideas, research questions, methods, and topics of articles within a theme into a summary paragraph.** | **Abstract:**   - Page 2   **Methods > Scoping Review:**   - Pages 7-8   **Results > Primary MIH Articles > Thematic Analysis:**   - Page 15 |
| **Reviewer 1** | ***Note 5.*** *It is not clear whether, the authors have included any grey literature sources such academic student research work, government reports, NGO reports and so on. If not, it is important to justify why and how this could affect the results. In one place they state that they used grey literature but in the limitations they state that they did not search for unpublished literature and so one is confused about which statement could be the correct one.* | **This is a terminology issue—we used an incorrect term and have fixed it. We did an informal “hand search” of citations** **in included full-text articles to find relevant articles meeting our inclusion criteria which were not identified by our database search.**  **There is no established term for this, but it is not a “grey search”. This “hand search” is accepted as a way to capture relevant research articles that may not be indexed in databases.**  **We did not conduct a grey search or consult grey literature. The exclusion of grey literature is mentioned in the inclusion criteria, but we have added an explicit note in the limitations section to enhance clarity.** | **Methods > Scoping Review:**   - Pages 6-7   **Results > Primary MIH Articles > Figure 1:**   - Page 10, PRISMA diagram   **Results > Primary MIH Articles:**   - Page 9   **Discussion > Strengths & Limitations:**   - Page 37 |
| **Reviewer 1** | ***Note 6.*** *How many of the studies were systematic reviews?* | **Systematic reviews were not included in this review, which is mentioned as part of the exclusion criteria.**  **We used the ambiguous word “review” in Table 1 which may lead to confusion. To clarify, table 1 now provides an accurate description, “desk review.”** | **Methods > Scoping Review:**   - Page 6   **Results > Primary MIH Articles > Table 1:**   - Page 11, Design   **Discussion > Strengths & Limitations:**   - Page 37 |
| **Reviewer 1** | ***Note 7.*** *Type of hospital is not reported in a single axis: both level of care and management style have been mixed up. For eg., Tertiary and government hospital are not mutually exclusive. It would be better to separate both these axis and present in Table 1. Similarly, focus of the data mainly talks about study population and the categories are not mutually exclusive, causing confusion in interpretation, for eg., patients can be population too, a more appropriate word would be community.* | **We have clarified the ambiguous language in Table 1 that gave rise to these two key comments. We have also amended the language in the results section to provide greater clarity.**  **See Table 1. Three Table headings have been revised to provide greater specificity and clarity. Please see Table 1 for edits.**  **See Results section, language has been clarified here as well.** | **Results > Primary MIH Articles > Table 1:**   - Page 11, Health facility based vs. community-based sample - Page 11-12, Type of health facility where study conducted - Page 12, Focus of data collected (level)   **Results > Primary MIH Articles > The Research Enterprise:**   - Page 13-14 |
| **Reviewer 1** | ***Note 8.*** *How did the authors manage non-English article data extraction and how was it standardised? Were there any Arabic literature?* | **We have revised the text to respond to this question and clarify these methods for the reader.**  **Data extraction, coding, and summary were done identically in French and English, as EA is bilingual French-English. However, standardization of data extraction for French articles was not possible, as none of the other authors speak French.**  **No Arabic language publications were found. If we had found Arabic articles, EA speaks Arabic and they would have been treated like the French language articles. We have revised Table 1 and the Results section to reflect n=0 Arabic articles found.** | **Methods > Scoping Review:**   - Page 7   **Results > Primary MIH Articles > Table 1:**   - Page 10, Language   **Results > Primary MIH Articles > The Research Enterprise:**   - Page 12   **Discussion > Strengths & Limitations:**   - Page 37 |
| **Reviewer 1** | ***Note 9.*** *The authors began with the premise that officially reported statistics may be different from realities on the ground and set the stage for clarification of this issue. However, neither the results nor the discussion addressed this in any way. The results highlight important areas for knowledge gap and unseen populations but that has nothing to do with discrepancies in reported and actual data. This needs to be addressed in some way in the analysis, perhaps a trend of MMR from the identified articles or an article(s) which highlights this issue. How do the authors substantiate this statement/conclusion – “Three research solutions emerged to broaden the research perspective and reconcile official and local Moroccan MIH realities: increase geographic breadth, address missing topics and populations, and embrace interdisciplinary methods.”* | **Thank you for this comment. The authors’ intent was not to claim that officially reported statistics are different than what is happening on the ground. This claim is beyond the scope of our review and would be more suited to a systematic review methodology.**  **Rather, we meant to show that indicators do not tell the whole story, and that MIH research is needed to provide a fine-grained picture of MIH in Morocco and help us to improve health for mothers and infants in Morocco.**  **We have revised the language to reflect our true focus, which is to describe the current research landscape, highlight research lacunae and blind spots, and suggest directions for future work.**  **We have revised the language to make our original premise clearer and to clarify our argument throughout the article.** | **Abstract:**   - Page 2   **Introduction:**   - Page 3 - Pages 4-5   **Discussion:**   - Pages 27-28   **Discussion > Gender, Health Equity, and Methodology:**   - Page 31   **Conclusion:**   - Page 37 |
| **Reviewer 1** | ***Note 10****. This line in abstract could be written clearly “Maternal health predominated, often >50% more than infant health.”* | **We have revised this sentence for more clarity.** | **Abstract:**   - Page 2 |
| **Reviewer 1** | ***Note 11.*** *The last date of literature search was Dec 2022 and one year has elapsed since. Therefore, it might be necessary to run the search for 2023 and see if any new articles have come up in the gap areas.* | **This recommendation is beyond the scope of the intended study and capacity of the team. We wished to provide a retrospective study over 22 years, 2000 up to 2022, and a resource for researchers, and to show the evolution of the field over time. By dividing the period equally into two 11 year periods, we could compare them in the analysis. Adding 2023 would unbalance the two groups and invalidate the comparison over time.** | **N/A** |
| **Reviewer 2** | ***Note 1.*** *There was a mention about disconnect between official statistics and some local Moroccan health realities. How was the data extraction and analysis handled from the studies during these period.* | **The intent of this review was not to compare official statistics with those reported in research studies. Therefore, this was not part of our data extraction process.**  **Our goal was to add context to indicators by summarizing the MIH research done over the last 22 years, and to provide a resource for researchers and government to help guide future work by identifying knowledge gaps. We have revised the language in the abstract, introduction, discussion and conclusions to clarify this point. Please see also the response to Reviewer 1, Comment 9.**  **Our data extraction process is outlined in the methods section. Briefly, we created data extraction sheets with drop down menus. Publication dates were extracted as part of this process. Data analysis was done grouping data according to the two time periods (2000-2011 and 2012-2022). Visual representations of proportions were presented to allow comparison between the two time periods.** | **Methods > Scoping Review:**   - Page 7   **See also:**  **Abstract:**   - Page 2   **Introduction:**   - Page 3 - Pages 4-5   **Discussion:**   - Pages 27-28   **Discussion > Gender, Health Equity, and Methodology:**   - Page 31   **Conclusion:**  Page 37 |
| **Reviewer 2** | ***Note 2.*** *Had the analysis been conducted with similar type of studies, proper statistical analysis could have been applied rigorously. Why was it not done?* | **For this study, we aimed to answer a broad research question about the overall state of MIH research in Morocco over the last 22 years. Our overall goal was to help map the types of studies and their focus to help find gaps in the literature for future work.**  **Broad questions like this are best addressed using a scoping review methodology due to the heterogeneous nature of the studies included. The broad nature of our review was to help provide a resource for researchers to help guide future work in MIH in Morocco.**  **A systematic review, as the reviewer suggests, would not have fit with our objective. Systematic review methodology would not be appropriate because systematic reviews address narrowly-focused questions. Also, as the reviewer notes, systematic reviews look only at similar types of studies. This again would not be appropriate for our study, which aims to provide the big picture overview of MIH research in Morocco.** | **N/A** |
| **Reviewer 2** | ***Note 3.*** *The title says this paper is a scoping review, then how could some data of network meta-analysis been performed.* | **We did not perform a meta-analysis. Rather, we assigned themes (primary, secondary, etc.) to each publication using qualitative research methods. We then utilized a network analysis software (Gephi) to visualize the relationships between these themes across the articles in this review. The resulting network thematic analysis visualization is Figure 6.**    **To clarify language, we changed the name of Figure 6 to “Thematic network visualization” to prevent any confusion.**  **The authors developed a thematic coding structure inductively, from a close and iterative reading of all articles (see revised methodology section). The themes were thus generated from our own analysis, and not part of the reported results of the included studies. The methodology section has been revised to clarify the qualitative methodologies used.** | **Methods > Scoping Review:**   - Pages 7-8   **Methods > Analysis:**   - Page 9   **Results > Primary MIH Articles > Thematic Analysis > Figure 6:**   - Page 27 |
| **Reviewer 2** | ***Note 4.*** *There is no clear data / tabulation on performance indicators of the policies adopted by the Moroccan health system like MMR, etc.* | **We thank the reviewer for this recommendation.**  **As the reviewer suggests researchers would find an overview of indicator metrics useful, we have taken WHO statistics and created an additional supplemental table of Moroccan MIH performance indicators, representing the two time periods, of 2000-2011 and 2012- 2022.** | **Newly created Supplemental Table 1 (Table S1):**   - Page 1, Table Supplement   **Introduction:**   - Page 3 |
| **Reviewer 2** | ***Note 5.*** *The themes of the research handled only women and infants. Geriatrics or men also form a part of their population. Data on these population may exhibit varied results on the outcomes* | **The topic of the review is maternal and infant health. The MeSH terms search strategy we used covered all prominent topics in that area (Table S2).**  **Men and geriatrics were not excluded, but they were not represented as MIH populations in the articles captured by the search. Women and infants were the only patient populations reported in the maternal and infant health articles. Men and geriatric persons were not represented as patients in MIH articles.**  **We do describe the very few studies that discuss husbands/men and geriatric persons’ knowledge and opinions about MIH:**   - **Men’s knowledge about MIH is mentioned in table 2 (Rural/Amazigh theme).** - **Men are also mentioned in the Discussion, in Table 3, under the rubric “Government Action.”** - **Elderly women are mentioned as *ferraga* healers in Traditional Medicine theme (Table 2)** - **Intergenerational transmission of traditional medical knowledge is also mentioned in Traditional Medicine theme (Table 2).** | **Please see:**  **Results > Primary MIH Articles >** **Thematic Analysis >** **Table 2:**   - Page 24, Rural/Amazigh - Page 26, Traditional Medicine   **Results > Primary MIH Articles > Application—Researcher Recommendations for Change > Table 3:**   - Page 36, Government Action   **Table S2:**   - Pages 2-4 Table Supplement |
| **Reviewer 2** | ***Note 6.*** *Has the protocol of scoping review been published anywhere ?* | **No, the protocol of the scoping review has not been published.** | **N/A** |
